# Escape from Pluripotency via Inhibition of TGF-β/BMP and Activation of Wnt Signaling Accelerates Differentiation and Aging in hPSC Progeny Cells

**DOI:** 10.1016/j.stemcr.2017.09.024

**Published:** 2017-10-26

**Authors:** Koki Fujimori, Takuya Matsumoto, Fumihiko Kisa, Nobutaka Hattori, Hideyuki Okano, Wado Akamatsu

**Affiliations:** 1 Department of Physiology, Keio University School of Medicine, Shinjuku-ku, Tokyo 160-8582, Japan; 2 Research Fellow of Japan Society for the Promotion of Science, Chiyoda-ku, Tokyo 102-0083, Japan; 3 Department of Neurology, Juntendo University School of Medicine, Bunkyo-ku, Tokyo 113-8431, Japan; 4 Center for Genomic and Regenerative Medicine, Juntendo University School of Medicine, Bunkyo-ku, Tokyo 113-8431, Japan

**Keywords:** induced pluripotent stem cells, stem cell differentiation, stem cell biotechnology, disease model, differentiation, pluripotency, aging

## Abstract

Human pluripotent stem cells (hPSCs) represent a potentially valuable cell source for applications in cell replacement therapy, drug development, and disease modeling. For all these uses, it is necessary to develop reproducible and robust protocols for differentiation into desired cell types. However, differentiation protocols remain unstable and inefficient, which makes minimizing the differentiation variance among hPSC lines and obtaining purified terminally differentiated cells extremely time consuming. Here, we report a simple treatment with three small molecules—SB431542, dorsomorphine, and CHIR99021—that enhanced hPSC differentiation into three germ layers with a chemically transitional embryoid-body-like state (CTraS). Induction of CTraS reduced the innate differentiation propensities of hPSCs (even unfavorably differentiated hPSCs) and shifted their differentiation into terminally differentiated cells, particularly neurons. In addition, CTraS induction accelerated *in vitro* pathological expression concurrently with neural maturation. Thus, CTraS can promote the latent potential of hPSCs for differentiation and potentially expand the utility and applicability of hPSCs.

## Highlights

• CTraS induction enhances hPSC differentiation into three germ layers without bias
• CTraS induction is applicable to a wide range of hPSCs even without colony selection
• Developing a robust neural induction protocol via CTraS for hPSC disease modeling
• CTraS induction promotes *in vitro* pathological expression with maturation and aging

## Graphical Abstract

**Figure:**
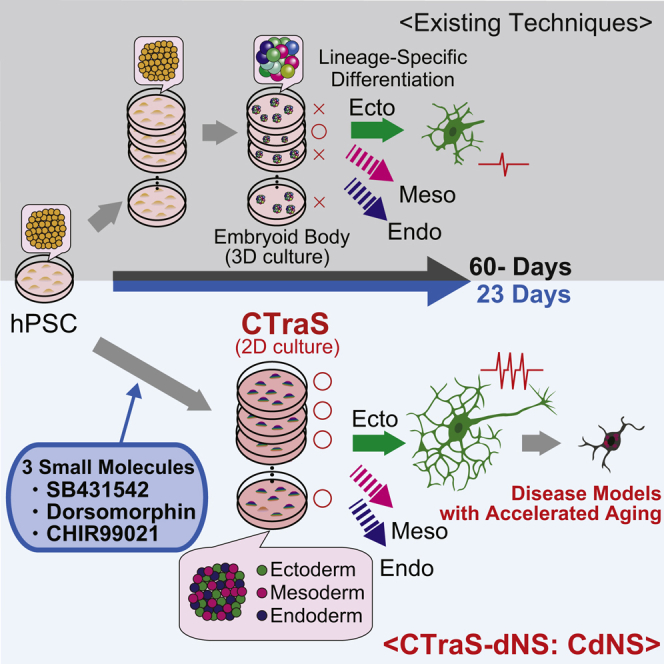

## Teaser

Simple treatment with three small molecules enhanced hPSC differentiation into three germ layers, namely CTraS. CTraS reduced the innate differentiation propensities of hPSCs and shifted them into terminal differentiations. CTraS induction accelerated *in vitro* pathological expression with maturation and aging. Thus, CTraS can bring out the latent potential of hPSCs.

## Introduction

Human pluripotent stem cells (hPSCs) have been in development for applications in cell replacement therapy (Okano et al., 2013, Tabar and Studer, 2014), drug discovery (Imamura et al., 2017), and hPSC disease modeling (Ichiyanagi et al., 2016, Imaizumi et al., 2015, Matsumoto et al., 2016) using patient-derived induced pluripotent stem cells (iPSCs). Although the development of reproducible and robust protocols for the differentiation into desired cell types will accelerate progress in these fields, differentiation protocols for several types of neural cells remain unstable and inefficient to obtain terminally differentiated cells without any specialized purification techniques (Matsumoto et al., 2016). In addition, individual hPSC lines are predisposed to differentiating into specific cell lineages, which may be influenced by the source cell type, donor, and reprogramming method (Kim et al., 2010, Kim et al., 2011, Osafune et al., 2008, Polo et al., 2010). To overcome this concern in neural disease modeling, we recently developed the direct neurosphere (dNS) conversion method (Fujimori et al., 2016, Matsumoto et al., 2016). Previous studies of early *Xenopus* development showed that dissociation of animal cap cells results in neuralization, presumably due to the loss of extracellular factors (Wilson and Hemmati-Brivanlou, 1995). BMP-4 and other extracellular signaling molecules present in the animal cap are known to repress neural development (Piccolo et al., 1996). Relevant to this, in our dNS method, PSCs are placed in a low-density floating culture to exclude all exogenous signals, including BMPs, to achieve efficient neural differentiation consistent with the default mechanism in neural fate specification exhibited by mouse embryonic stem cells (ESCs) (Nori et al., 2011), *Xenopus*, and iPSCs derived from T cells (Fujimori et al., 2016, Matsumoto et al., 2016).

Although we used a neural differentiation protocol involving embryoid body (EB) formation to prepare neural cells from hPSCs for use in pre-clinical studies in spinal cord injury(Kobayashi et al., 2012, Nori et al., 2011), the EB-based protocol was time consuming (∼2 months to induce neurospheres) and was unable to efficiently differentiate T cell-derived iPSCs (TiPSCs) (Matsumoto et al., 2016). Upon implementing EB-based neural differentiation protocols, several differentiation-resistant hPSC lines exhibited low-level expression of ectodermal markers at the EB stage and poor neurosphere (NS) formation from the dissociated EBs (Matsumoto et al., 2016). However, during the process of lineage-specific differentiation from PSCs, EB formation has been widely used to initiate spontaneous differentiation toward the three germ lineages, as it is a relatively simple method for obtaining lineage-committed cells in the mesodermal and endodermal lineages (Ng et al., 2005, Ogawa et al., 2013, Yang et al., 2008). During the EB formation process in high-density floating cultures in the presence of serum, non-committed PSCs receive various signals under 3D-culture conditions. We hypothesized that the presence of diverse extracellular signals interferes with efficient neural differentiation and that the various differentiation propensities of the PSC clones leads to differences in the cell distribution within the EBs. However, EBs provide a highly suitable environment for the maturation of committed cells.

Compared with cells cultured in 3D *in vitro* conditions, monolayer 2D-cultured cells are directly and homogeneously affected by exogenous factors in the culture medium. It has been reported that several small molecules can enhance and accelerate lineage-specific differentiation from hPSCs (Li et al., 2013). In the present study, we focused on the effects of SB431542 (SB), dorsomorphin (DM), and CHIR99021 (CHIR). SB has been implicated in efficient neural conversion of human ESCs (hESCs) and hiPSCs via inhibition of SMAD signaling in combination with Noggin activity (Chambers et al., 2012). Noggin, an inhibitor of BMP signaling, can be replaced by DM, which only enhances neural induction (Di-Gregorio et al., 2007). CHIR is an inhibitor of glycogen synthase kinase 3 (GSK3) and activates the canonical Wnt signaling pathway (Ring et al., 2003). Although the precise mechanism of Wnt signaling remains controversial, CHIR is often used to drive the induction of endodermal and/or mesendodermal specification, especially during early development (Clevers et al., 2014). Since these small molecules enhance different forms of germ-layer-specific differentiation through their effects on each pathway, we hypothesized that induced differentiation in a 2D culture environment by a defined combination of chemicals could give rise to cells at the transitional differentiation state that would be committed to all three germ layers in an unbiased manner.

In this study, we evaluated the effect of three small molecules on 2D cultures of undifferentiated hPSCs to induce intermediate progenitor cells. In addition, by differentiating these chemically induced cells (chemically transitional EB-like state [CTraS]) using conventional differentiation protocols, we demonstrated the potential of CTraS cells as core precursor cells for lineage-specific differentiation and as models of disease, particularly neurological disorders. In addition, CTraS induction is applicable to a wide range of hPSCs in that nearly all types of hPSCs can be induced to differentiate into neuronal cells without hPSC colony selection. Thus, CTraS could serve as a core intermediate progenitor to induce the differentiation of hPSCs irrespective of their innate differentiation propensities.

## Results

### Evaluation of Small Molecules to Accelerate the Differentiation of All Three Germ Layers from hPSCs

**Figure 1:**
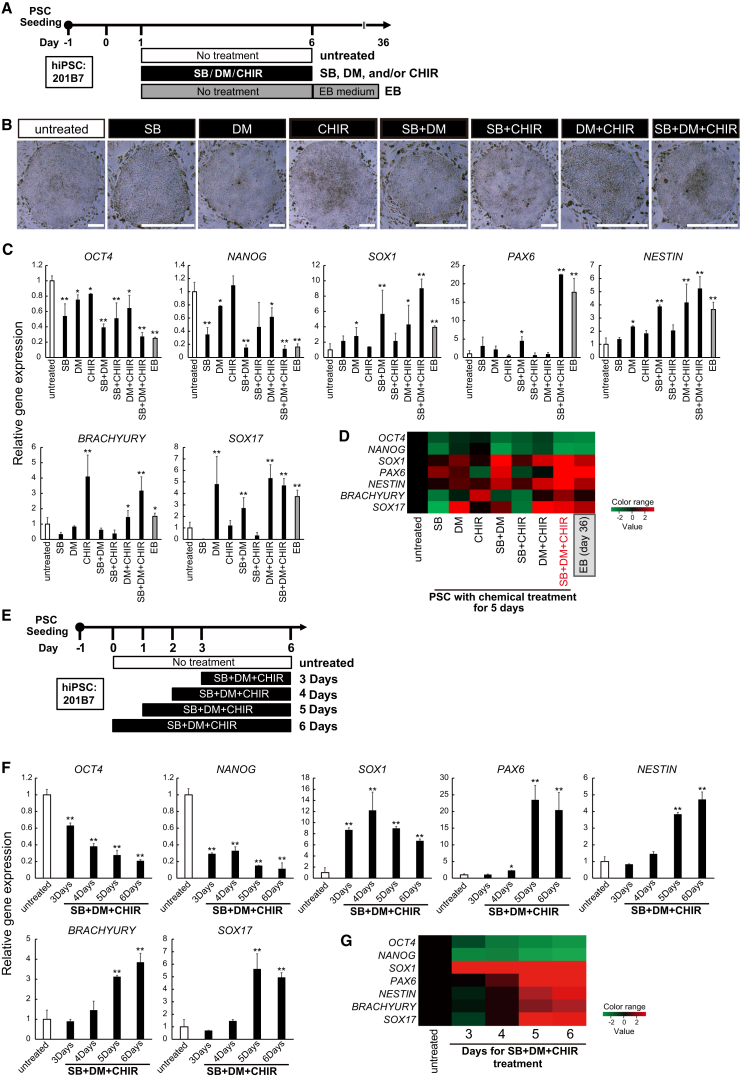
Evaluation of Small Molecules for Enhancing hPSC Differentiation (A) Schematic representation of experiments for screening combinations of hPSC differentiation enhancers. (B) Representative images of SB-, DM-, and/or CHIR-treated hPSCs. Scale bars, 200 μm. (C) qPCR analysis of the indicated genes in hiPSCs cultured under the indicated conditions for 5 days compared with 36-day EB cultures (n = 3 independent experiments; mean ± SEM; ^∗^p < 0.05, ^∗∗^p < 0.01; versus untreated; Dunnett’s test). (D) Heatmap summary of the qPCR analysis shown in (C). (E) Schematic of experiments for the time course of the treatment with the three small molecules (SB, DM, and CHIR). (F) qPCR analysis for the indicated genes in hiPSCs cultured with SB, DM, and CHIR for the indicated days (n = 3 independent experiments; mean ± SEM; ^∗^p < 0.05, ^∗∗^p < 0.01; versus untreated; Dunnett’s test). (G) Heatmap summary of the qPCR analysis shown in (F). hPSC line used: 201B7. See also Figures S1 and S2.

Optimal concentrations for each SB, DM, and CHIR treatment to hPSCs were determined based on the results of PSC colony morphologies and the expression of each germ-layer marker (Figures S1A and S1B). Undifferentiated hPSCs were treated with SB, DM, CHIR or a combination of the three compounds for 5 days on a feeder in the presence of fibroblast growth factor 2 (FGF-2) as shown in Figure 1A. These small molecules clearly affected the morphology of the hPSC colonies, and the diameter of colonies was significantly smaller than that of the untreated group, especially the SB + DM + CHIR group (Figures 1B and S1C–S1E). Combined treatment with all three inhibitors induced a significant decrease of the expression of pluripotent markers and an increase in the expression of germ-layer markers as well as the number of floating EBs (Figures 1C and 1D). These results indicate that treating hPSCs with SB, DM, and CHIR can cause the differentiation of these PSC colonies into EB-like 2D colonies on feeder layers.

To determine the optimal treatment duration with these small molecules, we next evaluated the hPSC morphology and the changes in the expression of pluripotent markers and germ-layer markers (Figures 1E–1G and S1H–S1J) and concluded that a 5-day treatment with the small molecules was best suited for inducting the differentiated state of hPSCs. The other two hPSC lines also showed similar changes after a 5-day treatment (Figures S2A and S2B). In addition, the differentiation-promoting effect of these three agents was more significant at the PSC stage compared with the later differentiation stage (Figures S2C–S2F). These results indicate that 5-day administration of SB, DM, and CHIR efficiently converts undifferentiated hPSCs into an EB-like state of differentiation, hereafter referred to as CTraS.

### Synergistic Inhibition of the GSK3, TGF-β, and BMP Signaling Pathways Enhanced the Endodermal, Mesodermal, and Ectodermal Differentiation of hPSCs

**Figure 2:**
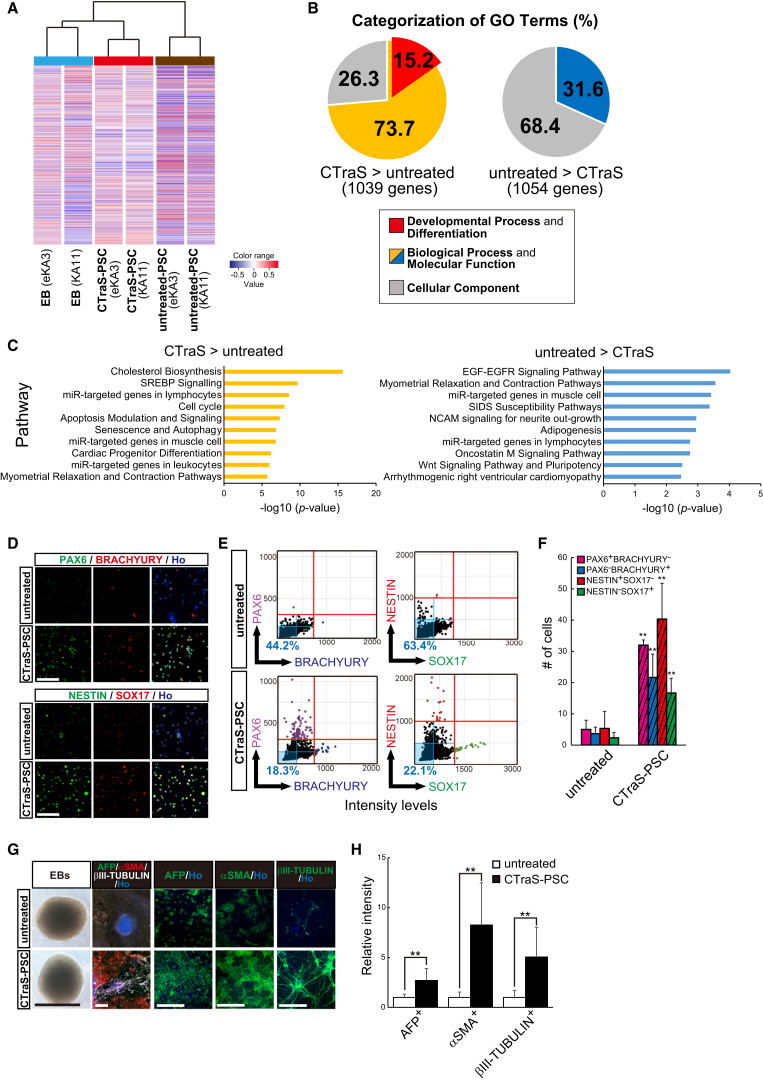
Synergistic Inhibition of the GSK3, TGF-β, and BMP Signaling Pathways Enhanced the Differentiation State of the hPSCs (A) Hierarchical clustering analysis of the global gene expression profiles of untreated PSCs, CTraS PSCs, and EBs. (B) Gene Ontology analysis of transcripts that were up- and downregulated in CTraS PSCs compared with the transcript levels in untreated PSCs (a fold change difference of ±2.0). (C) Top 20 pathways associated with the genes that were differentially expressed in CTraS PSCs compared with those in untreated PSCs (a fold change difference of ±2.0). hPSC lines used, KA11 and eKA3. (D) Immunostaining of single-cell dissociated untreated PSCs and SB + DM + CHIR-treated PSCs for the indicated tridermal lineage markers. Scale bars, 100 μm. (E and F) Cell population and analysis using single-cell dissociated untreated PSCs and CTraS PSCs stained for the indicated markers (n = 3 independent experiments; mean ± SEM; ^∗∗^p < 0.01; Student's t test). Intensity of markers (E) and numbers of cells (F) are shown. (G) Immunocytochemistry for the *in vitro* germ-layer assay and a representative image of EBs derived from untreated and CTraS PSCs. Scale bars, 200 μm. (H) Relative intensity of the indicated tridermal lineage markers in EBs induced from untreated or CTraS PSCs (n = 3 independent experiments; mean ± SEM; ^∗∗^p < 0.01; Student's t test). hPSC lines used: 201B7, WD39, and KhES1. See also Figures S2 and S3.

To explore differences in the signaling pathways affected by CTraS induction, we evaluated the global gene expression profiles in CTraS PSCs, untreated PSCs, and EBs. Hierarchical clustering analysis revealed that CTraS PSCs were grouped more closely with EBs than with untreated hPSCs (Figure 2A). Based on the hierarchical clustering, we extracted the gene set with different expression pattern in CTraS PSCs compared with EB and untreated PSCs. Pathway analysis using these gene sets demonstrated that cholesterol biosynthesis and its related pathways were remarkably promoted in CTraS induction (Figure S3). Next, we prepared a list of CTraS-regulated genes by selecting gene expression with fold changes >2.0. We analyzed the Gene Ontology (GO) terms of the genes (Table S1) and grouped them into three major classes; Biological Process, Molecular Function, and Cellular Component. We focused on the “Developmental Process and Differentiation” terms within in the “Biological Process” and “Molecular Function” groups to evaluate the effects of CTraS induction. Although there were no terms related to “Developmental Process and Differentiation” in the downregulated group, 15.2% of genes in the upregulated group contained development-related terms (Figure 2B). In addition, most terms in the upregulated group were not related to lineage specification (Table S1). These results also suggest that CTraS induction differentiates hPSCs into three germ layers. Pathway analysis identified multiple signaling pathways related to CTraS induction, including “cell cycle,” “apoptosis modulation and signaling,” and “senescence and autophagy,” indicating the enhancing effects on aging-related signaling at least in certain cell subpopulations (Figure 2C).

To evaluate how individual cells within CTraS-PSC colonies were altered by inhibitor treatment, we stained either colonies or dissociated single cells from untreated PSCs and CTraS PSCs for pluripotent markers and markers for all three germ layers. The intensities and frequencies of the pluripotent markers TRA-1-60 and SSEA4 were significantly decreased in CTraS colonies (Figures S1F and S1G). In addition, CTraS PSCs showed significant expression of markers representing all three germ layers (Figure 2D). Cell population analysis also revealed that CTraS induction decreased the number of undifferentiated cells and increased the number of differentiated cells in all the germ layers (Figures 2E and 2F). Next, to evaluate the effect of CTraS on differentiation ability, we formed EBs from CTraS PSCs and untreated PSCs. Although the morphologies seemed similar in both CTraS EBs and untreated EBs, the frequencies of the differentiation markers were significantly increased in CTraS-EB-derived cells (Figures 2G and 2H). These data indicate that the addition of SB, DM, and CHIR to hPSCs strongly enhances lineage-specific differentiation, resulting in the generation of cell clusters containing endodermal, mesodermal, and ectodermal cells on the feeder layers.

### CTraS Induction Accelerated Subsequent Differentiation with Lineage Specificity

**Figure 3:**
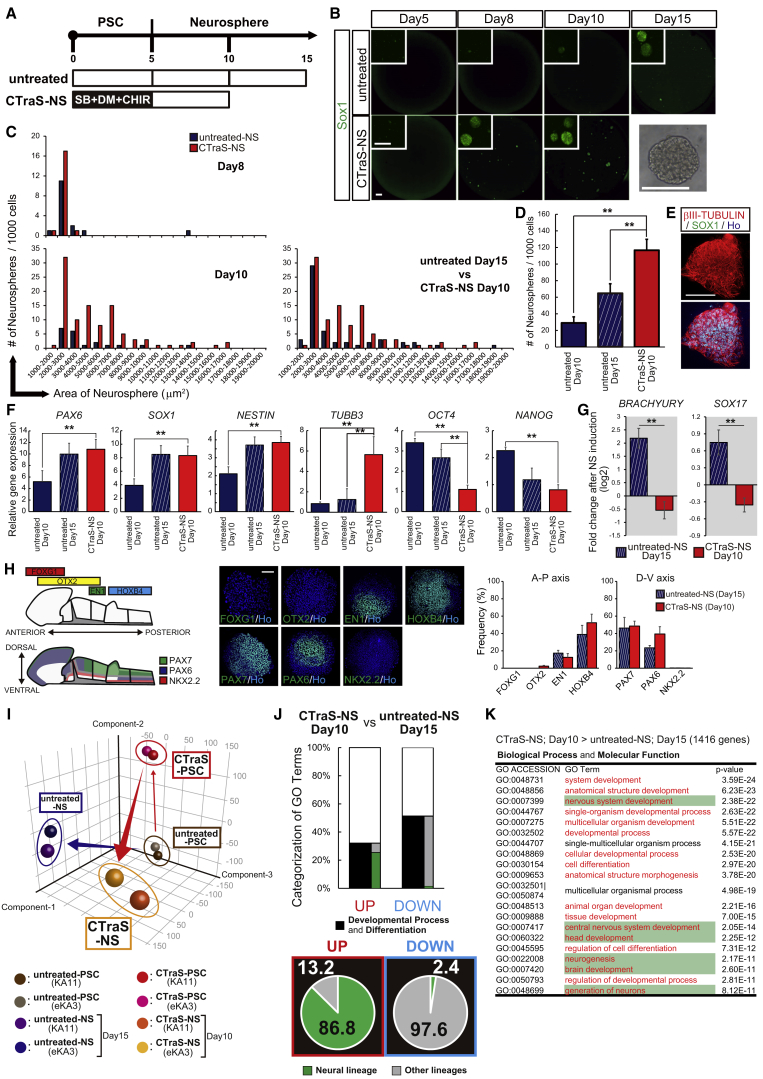
hPSCs Were Rapidly Differentiated toward the Neural Cell Lineage via CTraS Induction (A) Overview of the culture protocol in this experiment. (B–D) Sphere formation analysis of NSs derived from untreated PSCs and CTraS PSCs; SOX1 expression (B), relationship between the number and size (C), and total number (D) of the NSs were analyzed on the indicated day (n = 3 independent experiments; mean ± SEM; ^∗∗^p < 0.01; Dunnett's test). Scale bar, 400 μm. (E) Representative images of NSs at day 10 derived from CTraS PSCs with antibodies targeting the indicated markers. Scale bar, 200 μm. (F) qPCR analysis of the indicated markers in untreated-PSC- and CTraS-PSC-derived NSs (n = 3 independent experiments; mean ± SEM; ^∗∗^p < 0.01; Dunnett's test). (G) Fold change in the endoderm and mesoderm gene expression levels of untreated-PSC- and CTraS-PSC-derived NSs (n = 3 independent experiments; mean ± SEM; ^∗∗^p < 0.01; Student's t test). (H) Immunocytochemical analysis of NSs for A-P and D-V markers. The frequency of NSs containing immunopositive cells is shown as the percentage of total neurospheres (n = 3 independent experiments, mean ± SEM). Scale bar, 100 μm. hPSC lines used: 201B7, WD39, and KhES1. (I) Comparison of global gene expression profiles of untreated PSCs, CTraS PSCs, untreated NSs, and CTraS NSs. Principal component analysis of the gene expression data. Brown, untreated PSCs; red, CTraS PSCs; blue, untreated NSs; orange, CTraS NSs. (J) GO analysis of transcripts that were up- and downregulated in CTraS NSs compared with the transcript levels in untreated NSs (a fold change difference of ±2.0). (K) Top 20 GO terms associated with the upregulated genes in CTraS NSs compared with the levels in untreated NSs (a fold change difference of ±2.0). Red, developmental process and differentiation; green, neural lineage differentiation. hPSC lines used: KA11 and eKA3. See also Figure S4.

Next, we evaluated the differentiation propensity of CTraS-derived cells using dNS, which can differentiate most hiPSC clones (even those derived from blood cells) (Matsumoto et al., 2016). To adjust the induction periods, control cells were cultured for an additional 5 days in the NS formation (Figure 3A). We observed that the number of SOX1^+^ NS were markedly increased in the CTraS group at day 10 after NS induction in all the fractions compared with the control cells at day 15 (Figures 3B–3D).

PSC-derived NSs typically contain heterogeneous cell progeny containing original neural stem cells (NSCs), including NSCs themselves and their progeny (Hawes and Pera, 2006). To evaluate the effects of CTraS on the cell population in NSs, we quantified the expression of pluripotent markers and markers for all three germ layers. Although CTraS induction represented the time-dependent upregulation of the markers for all three germ layers, the expression of mesoderm and endoderm markers were significantly decreased during NS formation (Figures 3F–3G, S4A, and S4B). In addition, NSs from CTraS PSCs at day 10 significantly increased the expression of the neural marker *TUBB3*, and also expressed βIII-TUBULIN in the protein level (Figures 3E and 3F). A cell population analysis also clarified that the ratio of cells committed to the ectodermal lineage was apparently increased in CTraS-derived NSs at all time points measured (Figure S4C). By immunocytochemical analysis of formed NSs using representative markers in the anteroposterior (A-P) axis and dorsoventral (D-V) axis, we clarified that the cell population constituting CTraS NSs retained the region specificity on the dorsal side around the midbrain/hindbrain without significant difference from untreated NSs (Figure 3H).

To explore the detailed differences in neural differentiation between CTraS PSCs and untreated PSCs, we evaluated the global transcriptional profiles in NSs derived from these cells. Principal component analysis demonstrated that the gene expression patterns of NSs were significantly affected by CTraS induction (Figure 3I). GO analysis showed that in the category of “Developmental Process and Differentiation,” most of the extracted GO terms in the CTraS upregulated group were related to neural lineage, while those in the CTraS downregulated group were related to other lineages (Figures 3J, 3K, and S4D). In addition, in the “Biological Process and Molecular Function” category, the top 20 GO terms associated with CTraS upregulation contained 18 development-related terms, one-third of which were related to neural development (Figure 3K). These data support our cell population analysis data and strongly suggest that escape from pluripotency via CTraS induction not only accelerates subsequent differentiation but also enhances the lineage-specific differentiation depending on the surrounding environment.

### Efficient Generation of Functional Neurons Using Direct Neurosphere Conversion via CTraS

**Figure 4:**
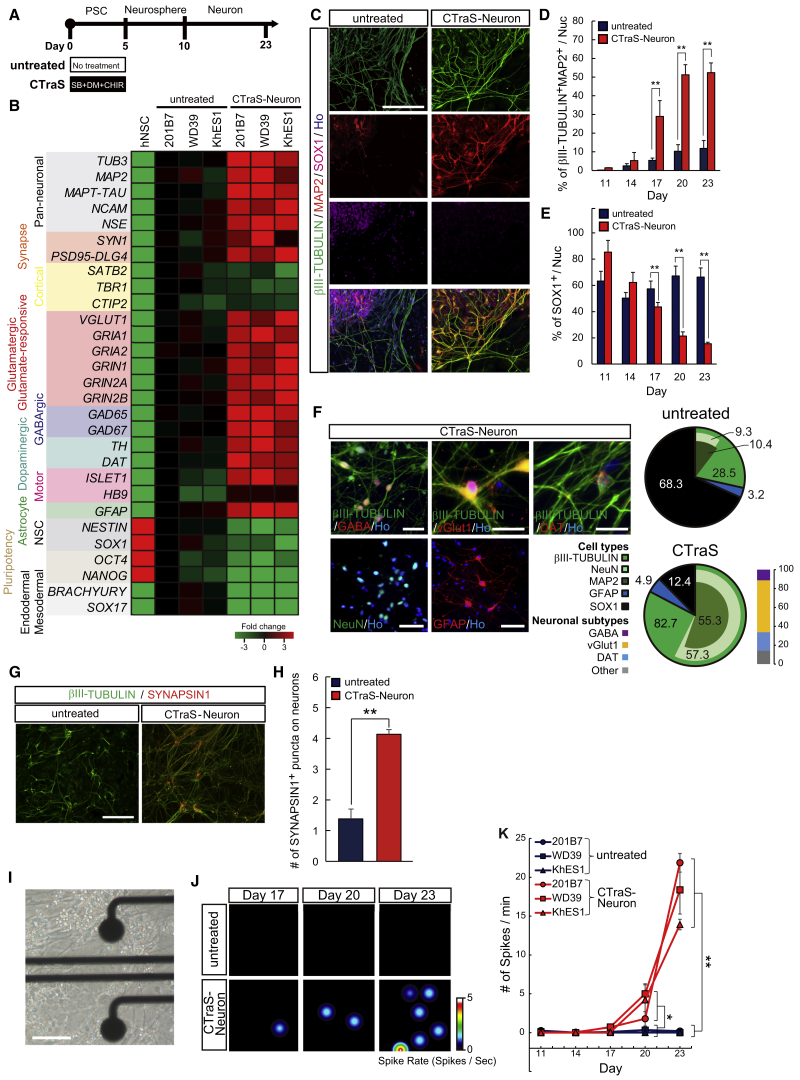
Efficient Generation of Functional Neurons Using Direct Neurosphere Conversion via CTraS (A) Overview of the culture protocol in this experiment. (B) Heatmap results derived from the qPCR analysis depicting the relative gene expression levels of the indicated markers. hNSC, hiPSC (201B7)-derived NSCs as described previously (Nori et al., 2011). (C) Representative images of terminally differentiated derivatives of NSs via CTraS PSCs or not with antibodies targeting the indicated markers. Scale bar, 100 μm. (D) Neural differentiation analysis quantifying the percentage of βIII-TUBULIN^+^MAP2^+^ cells (n = 3 independent experiments; mean ± SEM; ^∗∗^p < 0.01; Student's t test). (E) Residual neural stem cell analysis quantifying the percentage of SOX1^+^ cells (n = 3 independent experiments; mean ± SEM; ^∗∗^p < 0.01; Student's t test). (F) Cell population analysis of terminally differentiated derivatives of untreated PSCs and CTraS PSCs using dNS-based protocols. Scale bar, 50 μm. (G) SYNAPSIN1 expressions in CTraS and untreated neurons. Scale bar, 100 μm. (H) Neuronal maturation analysis indicated by the number of SYNAPSIN1^+^ puncta within βIII-TUBULIN^+^ neuronal cells at day 23 (n = 3 independent experiments; mean ± SEM; ^∗∗^p < 0.01; Student's t test). (I) Representative image of cultured neurons on the 64-electrode array on day 23. Scale bar, 100 μm. (J and K) Electrophysiological analysis of neurons derived from derived from NSs via CTraS-PSC or not using a microelectrode array (MEA) recording system (n = 3 independent experiments; mean ± SEM; ^∗^p < 0.05, ^∗∗^p < 0.01; Student's t test). hPSC lines used: 201B7, WD39, and KhES1. See also Figure S5.

We next examined whether CTraS NSs enhanced the terminal differentiation of neural cells (Figure 4A). After 13 days, differentiated cells from CTraS NSs showed increased expression of various neuronal and astrocyte markers and downregulated levels of NSC markers, pluripotent markers, mesoderm markers, and endoderm markers, while the expression of cortical neuron markers showed no significant difference between CTraS neurons and untreated neurons (Figures 4B, S5A, and S5B). Immunocytochemical analysis also revealed that differentiated CTraS NSs were mostly terminal differentiated cells, meanwhile those of untreated NSs were mostly NSCs with high proliferation potency (Figures 4C–4F and S5C–S5E). In addition, differentiation efficiency into astrocytes, which required differentiation and maturation at the NS stage, was also promoted by CTraS induction (Figures S5F–S5H). These data indicate that NSs derived from CTraS PSCs were rapidly differentiated into neuron and glia, resembling those found in the relatively posterior region between the midbrain and hindbrain and that the differentiated cell population had few residual stem cells.

We next sought to evaluate maturation of neural cells using the mature neuronal marker SYNAPSIN1 (Valtorta et al., 2011). Immunocytochemical analysis revealed that the number of SYNAPSIN1^+^ puncta on neurons was significantly increased in CTraS-NS-derived neurons (Figures 4G and 4H). Moreover, electrophysiological analysis using a microelectrode array (MEA) recording system demonstrated that CTraS-NS-derived neurons showed frequent spontaneous firing at 20 per day (Figures 4I–4K) while control cells never exhibited this activity. These data indicated that dNS-based neuronal differentiation protocols via CTraS (CTraS-dNS; CdNS) can efficiently accelerate neuronal differentiation into mature functional neurons.

### Neural Induction from Differentiation-Resistant ESC Lines and 30 Newly Established TiPSC Lines via CTraS

**Figure 5:**
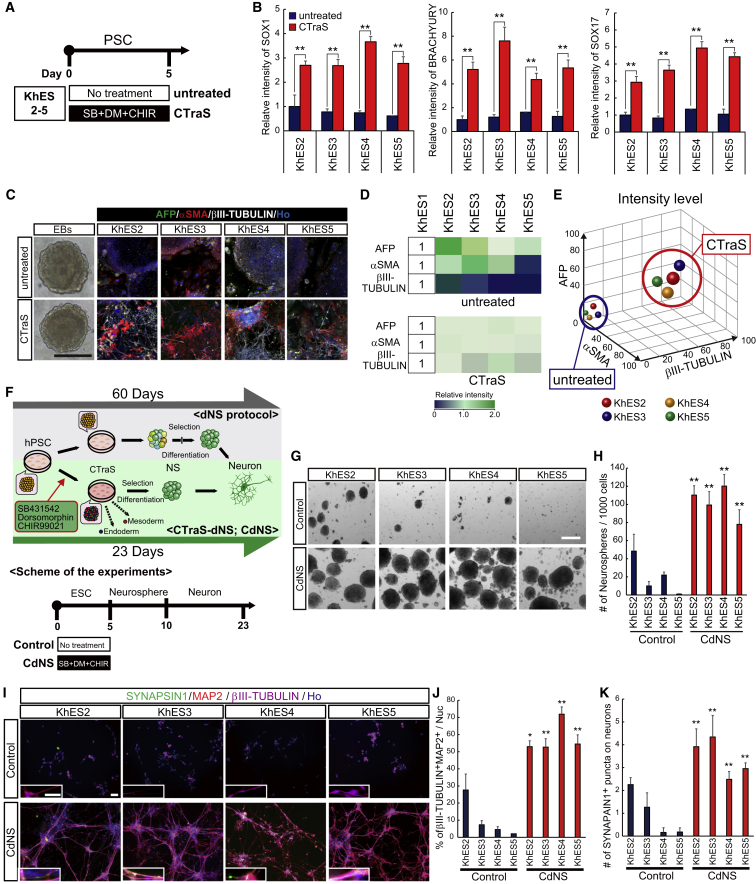
Neuronal Differentiation of Differentiation-Resistant hESC Lines via CTraS (A) Overview of the culture protocol for CTraS induction using KhESC lines. (B) Fluorescence intensities of the indicated tridermal lineage markers in untreated PSCs and CTraS PSCs (n = 3 independent experiments; mean ± SEM; ^∗∗^p < 0.01; Student's t test). (C) Representative image of EBs and immunocytochemistry based on the *in vitro* three germ-layer assay using KhESC lines. Scale bar, 200 μm. (D) Heatmap results derived from the fluorescence intensity analysis depicting the relative protein expression levels of the indicated markers. The fluorescence intensity levels were normalized to the mean level of each marker in KhES1 cells. (E) Fluorescence intensities of the indicated tridermal lineage markers in differentiated EBs induced from untreated PSCs and CTraS PSCs. (F) Schematic representation of dNS-based neuronal differentiation protocols via CTraS (CTraS-dNS; CdNS) and experimental scheme. (G) SYNAPSIN1 and MAP2 expressions in CTraS and untreated human ES-derived neurons. Scale bar, 200 μm. (H) Sphere formation analysis of NSs derived from KhESC lines as reflected by the quantification of the number and size of the NSs using the indicated methods (n = 3 independent experiments; mean ± SEM; ^∗∗^p < 0.01; Student's t test). (I) Immunostaining of KhESC-derived neurons with antibodies targeting the indicated markers. Scale bar, 100 μm. (J and K) Neuronal differentiation and maturation analysis as quantified by the percentage of βIII-TUBULIN^+^MAP2^+^ cells (J) and the number of SYNAPSIN1^+^ puncta in βIII-TUBULIN^+^ neuronal cells (K) (*n* = 3 independent experiments; mean ± SEM; ^∗^p < 0.05, ^∗∗^p < 0.01; Student's t test). hPSC cells used: KhES1, KhES2, KhES3, KhES4, and KhES5.

We sought to determine whether CTraS induction would improve the efficiency of neural differentiation of differentiation-resistant PSC clones. We evaluated four ESC lines (KhES 2–5), which exhibit insufficient neural differentiation via dNS. Using the CTraS induction protocol shown in Figure 5A, all four KhESC lines presented significantly increased expression of the markers of all three germ layers (Figure 5B). Although their differentiation patterns were slightly maintained in EBs even after CTraS induction, this treatment clearly reduced the differences among all four KhESC lines (Figures 5C and 5D) and accelerated their differentiation into all three germ layers (Figures 5C and 5E).

Then, to evaluate the effect of CTraS on NS formation and the subsequent neural differentiation of each KhESC line, we applied CdNS. Although all four KhESC lines tested showed poor NS formation and neural differentiation, CdNS significantly increased the number of NSs and showed highly efficient neural differentiation in 23 cultures from hPSCs (Figures 5F–5J). Immunocytochemical analysis revealed that the number of SYNAPSIN1^+^ puncta was also significantly increased in neurons derived from CTraS NSs in all four KhESC lines (Figure 5K).

**Figure 6:**
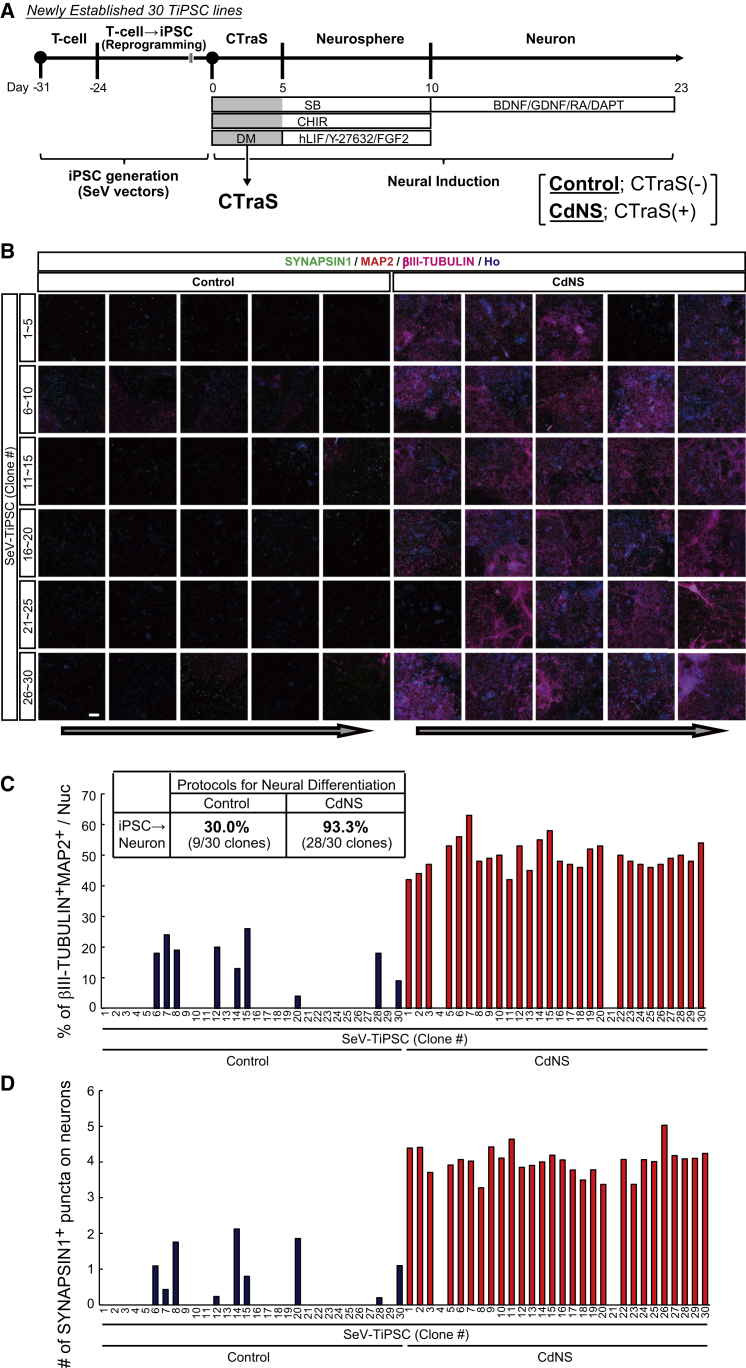
Efficient and Rapid Neuronal Differentiation of Newly Established TiPSCs without Colony Selection (A) An overview of the culture protocol in this experiment. (B) Immunostaining with antibodies targeting the indicated markers of neurons derived from 30 newly established TiPSC. Scale bar, 100 μm. (C) Neuronal differentiation analysis, quantification of the number of neuronal differentiated SeV-TiPSC lines, and their βIII-TUBULIN^+^MAP2^+^ cell ratios at day 23 (n = 1). (D) Neuronal maturation analysis of SYNAPSIN1^+^ puncta in βIII-TUBULIN^+^ neuronal cells at day 23 (n = 1). hPSC lines used: SeV-TiPSC (#1–#30, total 30 lines).

Next, we examined whether CTraS induction could improve the propensity for diverse differentiation of iPSC clones. Using Sendai virus, we established TiPSC clones from T cells obtained from a healthy donor. We randomly picked 30 independent colonies with typical hiPSC morphologies (SeV-TiPSC 1–30; 30 cell lines total). These clones were expanded and differentiated into neurons via NSCs using the CdNS protocol as shown in Figure 6A. Twenty-three days after neural differentiation, βIII-TUBULIN^+^ neurons were observed in nine SeV-TiPSC clones differentiated without CTraS induction. In contrast, 28 clones gave rise to βIII-TUBULIN^+^ neurons via CTraS induction over the same period (Figures 6B and 6C). The percentage of βIII-TUBULIN^+^ neurons apparently increased because of CTraS induction (Figure 6C). The number of SYNAPSIN1^+^ puncta was significantly higher in neurons derived from CTraS NSs in all the SeV-TiPSC lines (Figure 6D). These data indicate that most of the hiPSC clones efficiently differentiated into neurons following treatment by CdNS even without stringent clone selection.

### CTraS Induction Accelerated Disease-Specific Phenotypes in a Model of Neurodegenerative Disease

**Figure 7:**
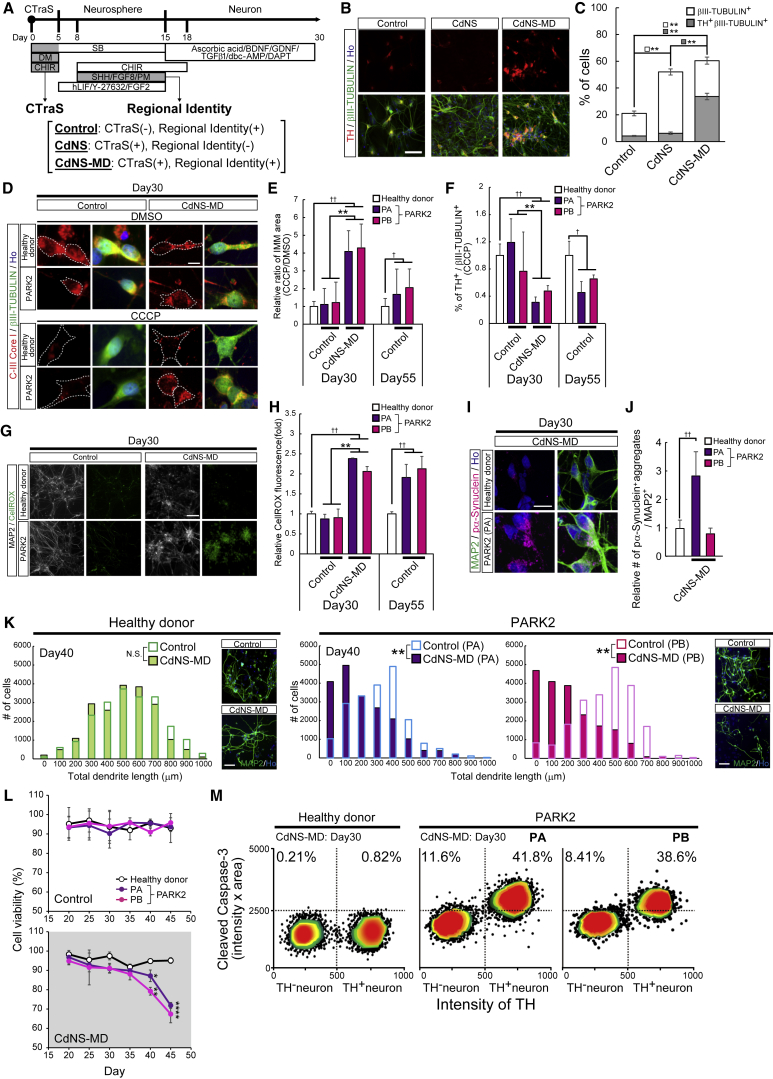
CTraS Induction Accelerates Age-Associated Changes and Disease-Specific Phenotypes in a Neurodegenerative Disease Model (A) Overview of the culture protocol in this experiment. (B) TH and βIII-TUBULIN expression in neurons from CdNS-MD, CdNS, and control NS. Scale bar, 100 μm. (C) Neuron and dopaminergic neuron differentiation analysis (n = 3 independent experiments; mean ± SEM; ^∗∗^p < 0.01; Tukey's test). (D) CIII-Core I and βIII-TUBULIN expressions in neurons from CdNS-MD and control NS. Scale bar, 50 μm. (E) Mitophagy analysis of the CCCP/DMSO ratio of the CIII-Core I in βIII-TUBULIN^+^ cells (n = 3 independent experiments; mean ± SEM; ^∗∗^p < 0.01 control versus CdNS-MD; †p < 0.05, ††p < 0.01 healthy donor versus PARK2; Student's t test). (F) Stress vulnerability analysis based on the ratio of TH^+^ neurons after CCCP treatment (n = 3 independent experiments; mean ± SEM; ^∗∗^p < 0.01 control versus CdNS-MD; †p < 0.05, ††p < 0.01 healthy donor versus PARK2; Student's t test). (G) MAP2 immunostaining and CellROX fluorescence in neurons from CdNS-MD and control NS. Scale bars, 500 μm. (H) Oxidative stress analysis of PARK2 neurons and neurons from a healthy donor (n = 3 independent experiments; mean ± SEM; ^∗∗^p < 0.01 control versus CdNS-MD; ††p < 0.01 healthy donor versus PARK2; Student's t test). (I) MAP2 and pα-syn expressions in neurons from CdNS-MD and control NS. Scale bar, 50 μm. (J) Quantitative analysis of pα-syn accumulation in neurons differentiated from iPSCs from a healthy donor and PARK2 iPSCs (n = 3 independent experiments; mean ± SEM; ††p < 0.01 healthy donor versus PA; Student's t test). (K) Dendrite length analysis of iPSC-derived neurons from a PARK2 patient compared with those from healthy donors (n = 3 independent experiments; mean ± SEM; ^∗∗^P < 0.01; Kolmogorov-Smirnov test). Scale bar = 50 μm. (L) Cell viability analysis of iPSC-derived neurons from a PARK2 patient compared with those from healthy donors (n = 3 independent experiments; mean ± SEM; ^∗^p < 0.05, ^∗∗^p < 0.01 healthy donor versus PARK2; Student's t test). (M) Apoptotic cell population analysis of iPSC-derived neurons from a healthy donor and iPSC-derived neurons from PARK2 patients as gated on their TH intensities. (D–H, L: n = 3 independent experiments; mean ± SEM; ^∗∗^p < 0.01 Control vs CdNS-MD; †p < 0.05, ††p < 0.01 healthy donor vs PARK2; Student's t-test). hPSC lines used: KA11, KA23, and eKA3 (healthy donor); PA1, PA9, and PA22 (PARK2-PA); PB2, PB18, and PB20 (PARK2-PB). See also Figures S6 and S7.

We examined whether CTraS induction could accelerate *in vitro* aging to efficiently detect phenotypes associated with a late-onset neurodegenerative disease model. We first used iPSC clones derived from a patient with autosomal recessive juvenile Parkinson's disease (PD) due to the loss of PARK2 activity. Since midbrain dopaminergic neurons (mDANs) are selectively damaged in PD, we first modified the CdNS protocol to provide the regional identity around the midbrain (CdNS-MD) (Figure 7A) using Sonic Hedgehog (SHH) and FGF-8 (Gale and Li, 2008). qPCR and immunocytochemical analysis showed increased expression of mDAN markers/populations in both NSs and neurons induced by CdNS-MD compared with the original unbiased CdNS protocol (Figures S6A–S6D). Although both CdNS-MD- and CdNS-derived NSs differentiated into βIII-TUBULIN^+^ neurons at a similar frequency by day 30, the number of TH^+^ dopaminergic neurons was increased among the CdNS-MD-derived cells (Figures 7B and 7C). Before the pathological analysis, we confirmed that the iPSC lines used for the analysis showed similar differentiation efficiency in NS formation and mDAN induction by CdNS-MD (Figures S6E and S6F).

To detect PARK2-specific mitochondrial phenotypes, we treated mDANs derived via CTraS with carbonyl cyanide m-chlorophenylhydrazone (CCCP), which triggers mitophagy by disrupting the mitochondrial membrane potential (Fujimori et al., 2016, Imaizumi et al., 2012, Matsumoto et al., 2016). Although PARK2 neurons at day 30 did not show an accumulation of impaired mitochondria in neurons without CTraS (Figures 7D and 7E), the mDANs induced via CTraS clearly demonstrated PARK2-specific phenotype by day 30 (Figures 7D and 7E) accompanied by a significant decrease of the number of mDANs (Figure 7F). The observed increase in reactive oxygen species production (Fujimori et al., 2016, Matsumoto et al., 2016) only manifested in PARK2 mDANs induced via CTraS (Figures 7G and 7H). Immunocytochemical analysis demonstrated pα-synuclein aggregation (Athauda and Foltynie, 2015) in the neuronal cytoplasm of PARK2-PA cells at day 40 that were derived from the CdNS-MD method (Figures 7I and 7J) with the breakdown of established neurites (Figure 7K). Cell viability analysis using MTT showed a significant decrease of viable PARK2 neurons derived from CTraS after 40 days (Figure 7L). We also observed a significant increase in condensed nuclei that expressed cleaved caspase-3 in iPSC neurons from the PD patient compared with those from a healthy donor, especially among TH^+^ dopaminergic neurons (Figure 7M), indicating that PARK2 dopaminergic neurons are more prone to activating cell death programming upon CTraS. Such promotion of *in vitro* pathology via CTraS was also confirmed in familial amyotrophic lateral sclerosis (ALS) models carrying *TARDBP* mutations; the neurite swellings and the reduction of cell viability were reproduced with a shorter culture period by CTraS induction (Figure S7). These data clearly indicate that CTraS induction efficiently accelerates *in vitro* aging to detect phenotypes from early to terminal stage in neurological disease models.

## Discussion

In the present study, we have shown that treatment with three small molecules, SB, DM, and CHIR, effectively enhanced the differentiation of hPSCs and changed their state toward a chemically transitional EB-like state, which we have designated CTraS. Interestingly, hPSC-derived cells with CTraS induction differentiated into their respective progenies significantly faster than those without CTraS induction. Using this approach, we demonstrate two advantages of CTraS induction in hiPSC generation and disease modeling. First, stringent colony selection of newly generated PSC clones is not required to eliminate clones that are resistant to differentiation using conventional protocols. Second, CTraS induction accelerated *in vitro* neural maturation and progressive cellular phenotypes in models of neurodegenerative diseases, including PD and ALS. In a previous study, more than 100 days of monolayer differentiation were required to detect an apparent phenotype among neurons derived from disease-specific hiPSCs (Imaizumi et al., 2012, Ohta et al., 2015). By contrast, our method recapitulates several aspects of PD from early to terminal stage after only 30–40 days of culture (Figures 7D–7M). These results suggest that CTraS induction recapitulates aging phenotypes within a shorter period without requiring exogenous factors such as progerin expression and RanBP17 knockdown (Mertens et al., 2015, Miller et al., 2013).

The small molecules we used for CTraS induction have been used in various differentiation protocols (Li et al., 2013). Some researchers have reported the efficacy of the combination of SB, DM, CHIR and/or the other chemicals with the same target pathways to achieve lineage-specific differentiation from PSCs (Chambers et al., 2012, Kriks et al., 2011, Li et al., 2011, Lian et al., 2012). However, these reports referred to their effects on particular lineages, especially in the efficiency of lineage commitment, and there have been few observations regarding the characteristics of the differentiated cells and the other lineages. The outcomes of the SB + DM + CHIR treatment observed in our study are significantly different from these reports. Induced differentiation of hPSCs in a 2D culture using SB + DM + CHIR gives rise to cells at the transitional differentiation state that are committed to all three germ layers, which minimizes the differentiation bias. Based on a global gene expression analysis, the top ten pathways upregulated by CTraS included “cell cycle,” “apoptosis modulation and signaling,” and “senescence and autophagy,” indicating that CTraS augments cellular aging in addition to promoting unbiased differentiation. At present, we cannot exclude the possibility that the enrichment of these pathways indicates the increase of apoptosis due to the addition of exogenous factors. In addition to this concern, progress in the study regarding the mechanism of aging acceleration by CTraS is expected in the future. As one of the molecular biological characteristics of CTraS PSCs, enhanced cholesterol biosynthesis and the mevalonate pathway have been identified. In recent studies, Okamoto-Uchida and colleagues showed that the mevalonate pathway is essential in primitive streak formation using mouse ESCs (Okamoto-Uchida et al., 2016), suggesting the association with the effect of CTraS in terms of differentiation promotion in the PSC stage. From the accumulation of such findings, it is expected that future studies will uncover the whole mechanism of CTraS.

Wnt/β-catenin signaling is known to maintain the balance of self-renewal and differentiation in PSCs in a context-dependent fashion. An imbalance of this activity, such as dysfunction of the transcriptional network underlying pluripotency, promotes differentiation (Abu-Remaileh et al., 2010, Davidson et al., 2012). In addition, several recent studies have reported that Wnt signaling regulates aging in various tissues and stem cells (Chen and Do, 2012, Fujimaki et al., 2015, Naito et al., 2012). In our culture protocol described here, CHIR, which functions as a Wnt activator via GSK-3β inhibition, was used during both the CTraS and NS induction periods. It is possible that activated Wnt signaling influences both terminal differentiation and aging, which may accelerate pathological expression *in vitro*, in the differentiated cells.

Although the detailed molecular mechanisms of CTraS induction via SB + DM + CHIR remain unclear, our results suggest the usefulness of CTraS induction as a general technology for hPSC differentiation. On the basis of our findings, the expansion of CTraS application to the feeder-free culture system without serum replacement in the future is expected to further increase its usefulness. This system may thus contribute to new insights in disease modeling, drug screening, and regenerative medicine using PSCs.

## Experimental Procedures

### Culture of Undifferentiated ESCs and iPSCs

The human ESC lines KhES1, KhES2, KhES3, KhES4, and KhES5, the control human iPSC lines 201B7, WD39, KA11, KA23, and eKA3, and the PARK2 iPSC lines PB2, PB18, and PB20 were cultured on mitomycin C-treated SNL murine fibroblast feeder cells in standard hESC medium (DMEM/F12, Sigma-Aldrich) containing 20% KnockOut serum replacement (KSR) (Life Technologies), 0.1 mM non-essential amino acids (Sigma-Aldrich), 0.1 mM 2-mercaptoethanol (Sigma-Aldrich), and 4 ng/mL FGF-2 (PeproTech) in an atmosphere containing 3% CO_2_. hESCs were used in accordance with the guidelines regarding the utilization of hESCs with approval from the Ministry of Education, Culture, Sports, Science, and Technology (MEXT) of Japan and the Keio University School of Medicine Ethics Committee. All experimental procedures involving iPSCs derived from patients were approved by the Keio University School of Medicine Ethics Committee (approval no. 20080016).

### Isolation of Human T Cells and Generation of TiPSCs

Peripheral blood mononuclear cells (PBMCs) were obtained from a healthy donor (race, Japanese; sex, male; age, 26 years) by centrifuging heparinized blood over a Ficoll-Paque PREMIUM gradient (GE Healthcare) according to the manufacturer's instructions. CD3-positive cells were selected using a fluorescently conjugated anti-CD3 mAb (BD Pharmingen). PBMCs and cells subjected to fluorescence-activated cell sorting were seeded on a plate coated with an anti-CD3 mAb and cultured at 37°C in 5% CO_2_ in GT-T502 medium (KOHJIN BIO) containing 175 JRU/mL rIL-2. After 5 days of culture, activated PBMCs and activated T cells were transferred to a 96-well plate coated with an anti-CD3 mAb at a density of 1.5 × 10^3^ cells/well and incubated for an additional 24 hr. Thereafter, a solution containing SeV vectors (CytoTune-iPS; ID pharma) was added to the wells. At 24 hr post infection, the medium was replaced with fresh GT-T502 medium. At 48 hr post infection, the cells were collected and transferred to a 96-well plate containing mitomycin C-inactivated SNL feeder cells. After an additional 24 hr, the medium was replaced with hiPSC medium, which was changed every other day until colonies were selected. The generated hiPSCs were maintained on mitomycin C-inactivated SNL feeder cells in hiPSC medium. Healthy donor TiPSC lines (total 30 lines; SeV-TiPSC 1–30) were cultured, and cells at low passage numbers (between 2 and 5) were used for analysis.

## Author Contributions

K.F., T.M., N.H., H.O., and W.A. conceived and designed the experiments. K.F., T.M., F.K., and W.A. performed the experiments and analyzed data. K.F., H.O., and W.A. wrote and edited the manuscript. N.H. contributed reagents, materials, and analysis tools. All authors read and approved the final manuscript.

## Accession Numbers

The data reported in this publication have been deposited in the NCBI Gene Expression Omnibus database and are accessible under accession number GEO: GSE95271.

## Supplemental Information

Document S1. Supplemental Experimental Procedures, Figures S1–S7, and Table S1–S3

Document S2. Article plus Supplemental Information
