# Supplementary material for: Escape from Pluripotency via Inhibition of TGF-β/BMP and Activation of Wnt Signaling Accelerates Differentiation and Aging in hPSC Progeny Cells
Source: Stem Cell Reports. 2017 Oct 26;9(5):1675–91. doi: 10.1016/j.stemcr.2017.09.024 (PMC5831048; doi:10.1016/j.stemcr.2017.09.024)
Supplement: Document S1. Supplemental Experimental Procedures, Figures S1–S7, and Table S1–S3 [file mmc1.pdf]

Stem Cell Reports, Volume *9*

Supplemental Information

Escape from Pluripotency via Inhibition of TGF-β/BMP and Activation of Wnt Signaling Accelerates Differentiation and Aging in hPSC Progeny Cells

Koki Fujimori, Takuya Matsumoto, Fumihiko Kisa, Nobutaka Hattori, Hideyuki Okano, and Wado Akamatsu

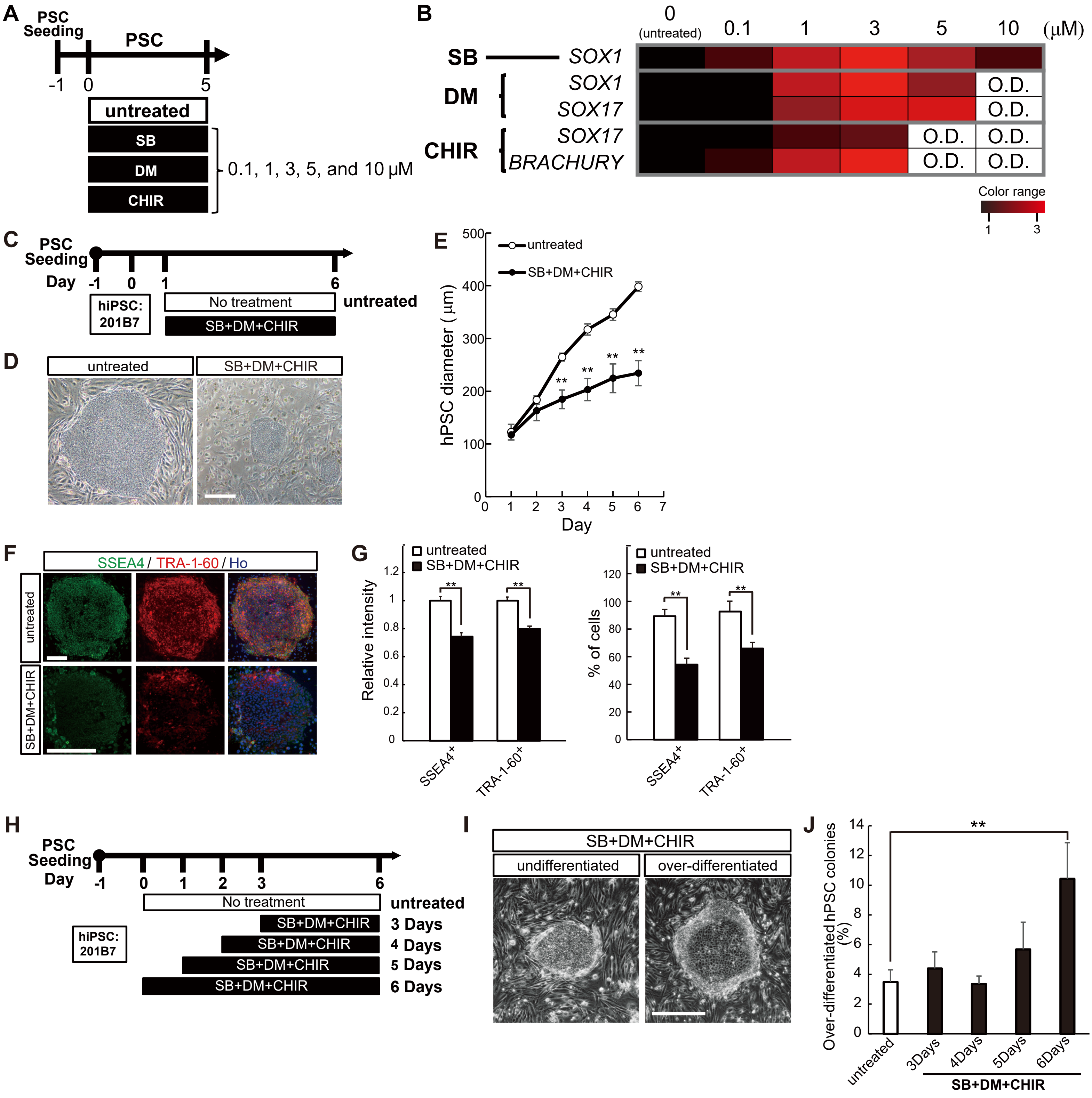

Figure S1 Effects of SB, DM, and/or CHIR Treatment on the Lineage-Specific Differentiation, Morphology, and Pluripotency of hPSC. (related to Figure 1)

(A) Overview of the culture protocol in this experiment. (B) qPCR analysis of three-germ layers markers in PSC treated SB, DM, or CHIR with indicated dose for 5 days (n=3 independent experiments). "O.D." indicates Over Differentiated PSC colony showing doughnut-like morphology. (C) Schematic of the experiments regarding the time-course of treatment with the three small molecules. (D) Representative images of untreated hPSCs and SB-, DM-, and CHIR-treated hPSCs. Scale bars = 200 μm. (E) Differences in the colony size of untreated hPSCs and SB+DM+CHIR treated-hPSCs (n = 3 independent experiments; mean ± SEM; $**P < 0.01$; Student's *t*-test). (F) Immunostaining of untreated hPSCs and SB+DM+CHIR-treated hPSCs for the indicated pluripotent markers. (G) Intensity and frequency of the indicated pluripotent markers in untreated hPSCs and SB+DM+CHIR treated-hPSC (n = 3 independent experiments; mean ± SEM; $**P < 0.01$; Student's *t*-test). (H) Schematic of experiments for the time-course of three small molecules treatment. (I) Representative morphology of undifferentiated hPSCs (untreated hPSCs) and over-differentiated hPSCs (6 Days SB+DM+CHIR-treated hPSCs) on day 6 after hPSC seeding. Scale bars = 200 μm. (J) Percentage of over-differentiated hPSC colonies (n = 3 independent experiments; mean ± SEM; $**P < 0.01$; Dunnett's test). hPSC lines used: 201B7

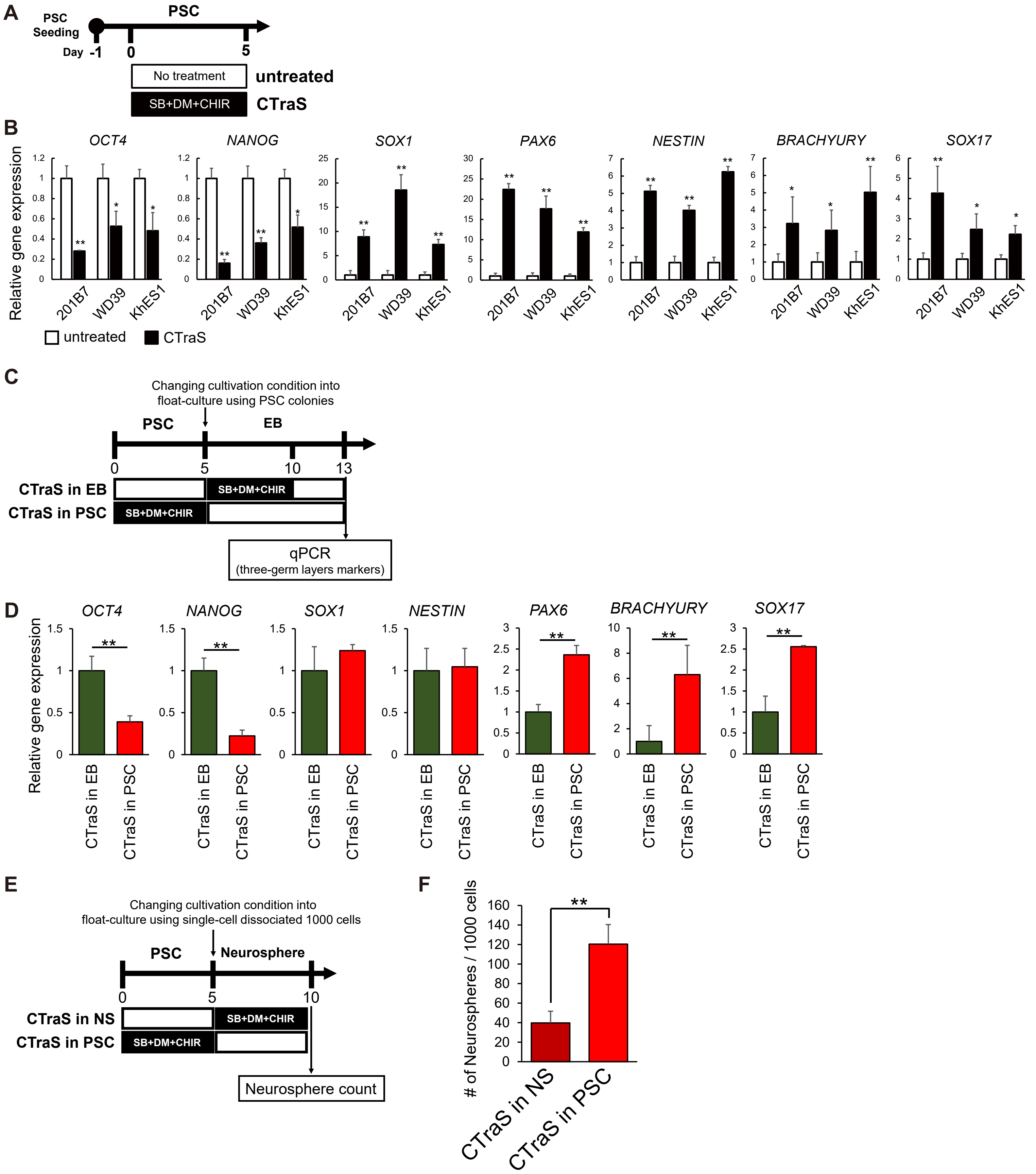

Figure S2 Enhancement of the Three-Germ Layers Differentiation via CTraS in PSC-Stage. (related to Figure 1 and 2)

(A) Overview of the culture protocol used in this experiment. (B) qPCR analysis for the indicated genes in three hPSC lines cultured with SB, DM, and CHIR for 5 days (n = 3 independent experiments; mean ± SEM; *$P < 0.05$, **$P < 0.01$; Student's *t*-test). (C) Overview of the culture protocol in this experiment. (D) qPCR analysis of pluripotent markers and three-germ layers markers in EBs with CTraS induction at two different stages of differentiation (n=3 independent experiments; mean ± SEM; **$P < 0.01$; Student's *t*-test). (E) Overview of the culture protocol in this experiment. (F) Sphere formation analysis of neurospheres (n=3 independent experiments; mean ± SEM; **$P < 0.01$; Student's *t*-test). hPSC lines used: 201B7, WD39, and KhES1

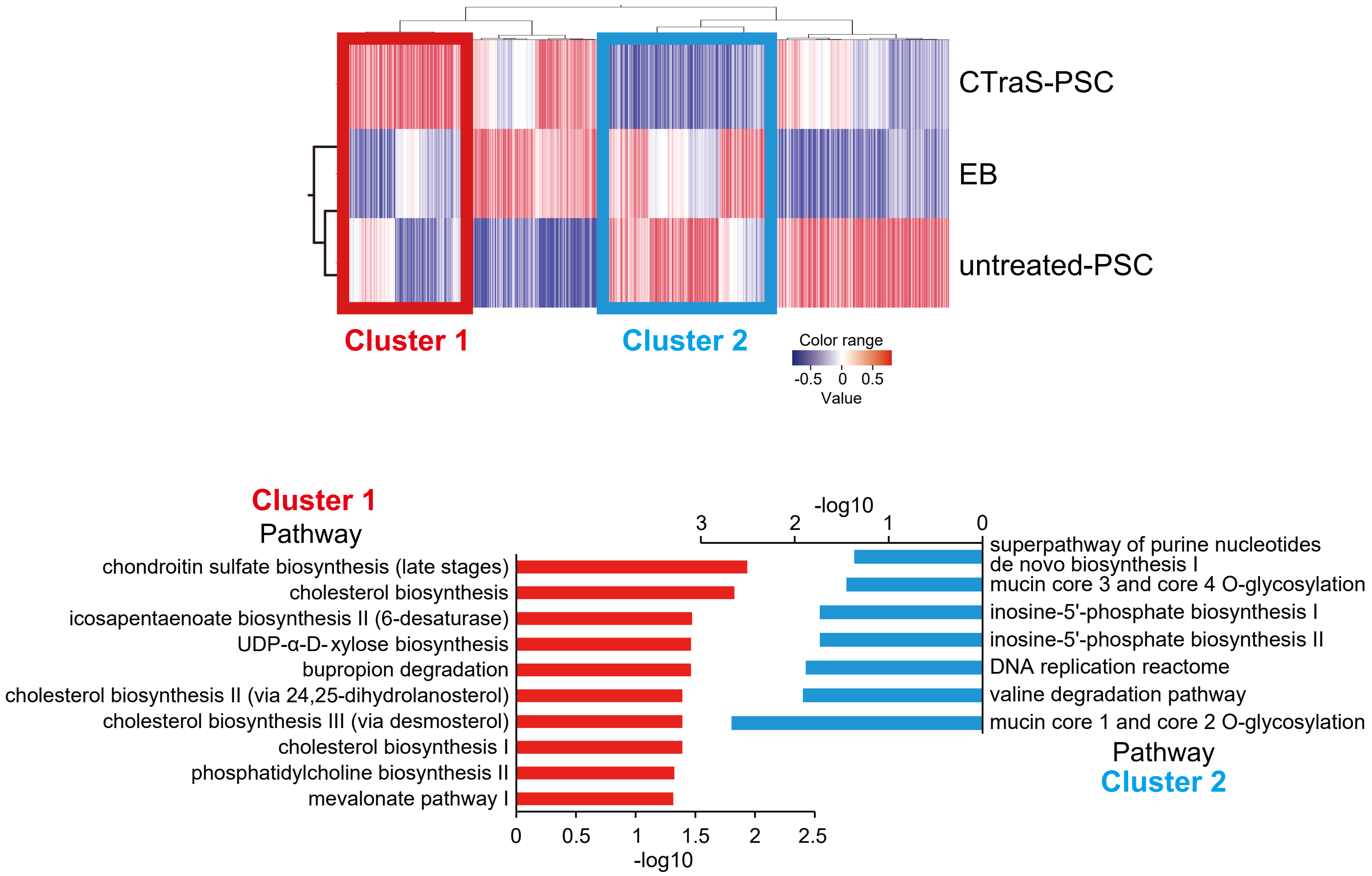

Figure S3 Pathway Analysis of the Gene-Set Showing the CTraS-Specific Expression Pattern. (related to Figure 2)

Pathway analysis of the extracted gene-set with different expression pattern in CTraS-PSC compared with EB and untreated-PSC. The data reported in this publication have been deposited in the NCBI Gene Expression Omnibus (GEO, http://www.ncbi.nlm.nih.gov/geo/) database and are accessible using GEO Series accession number GSE95271. hPSC line used; KA11 and eKA3

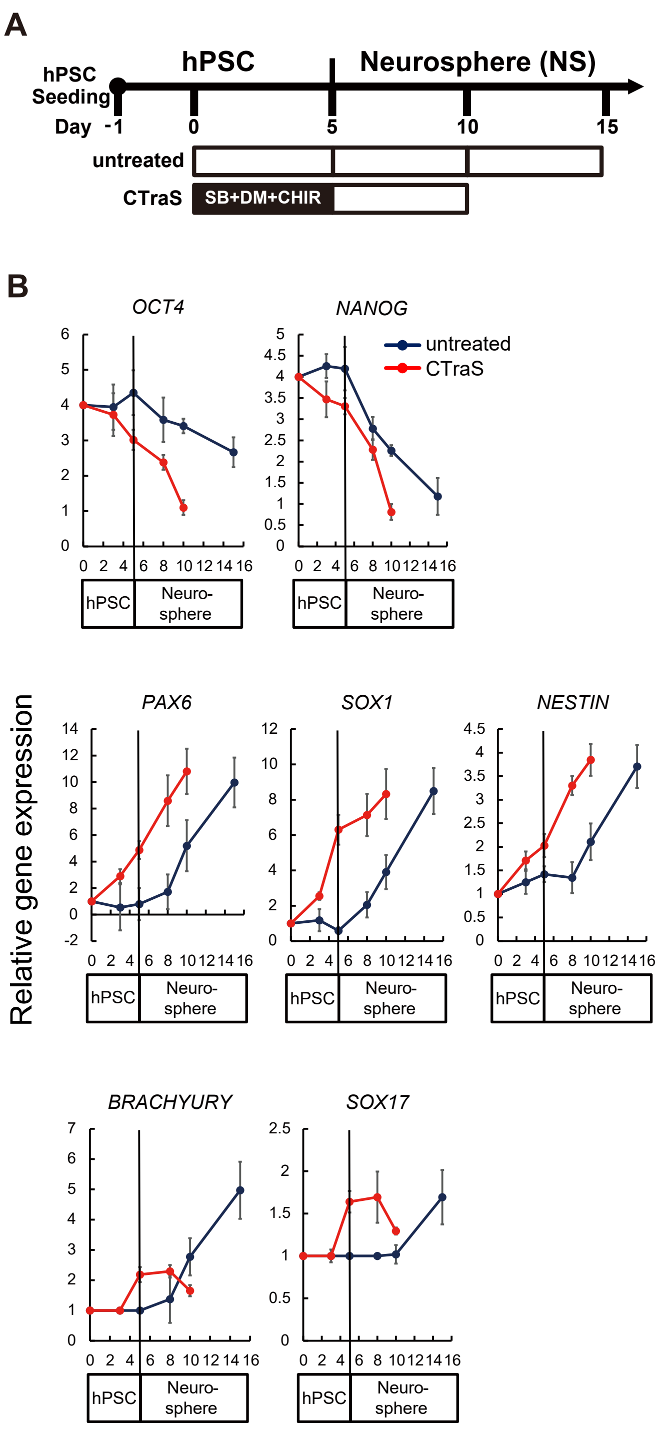

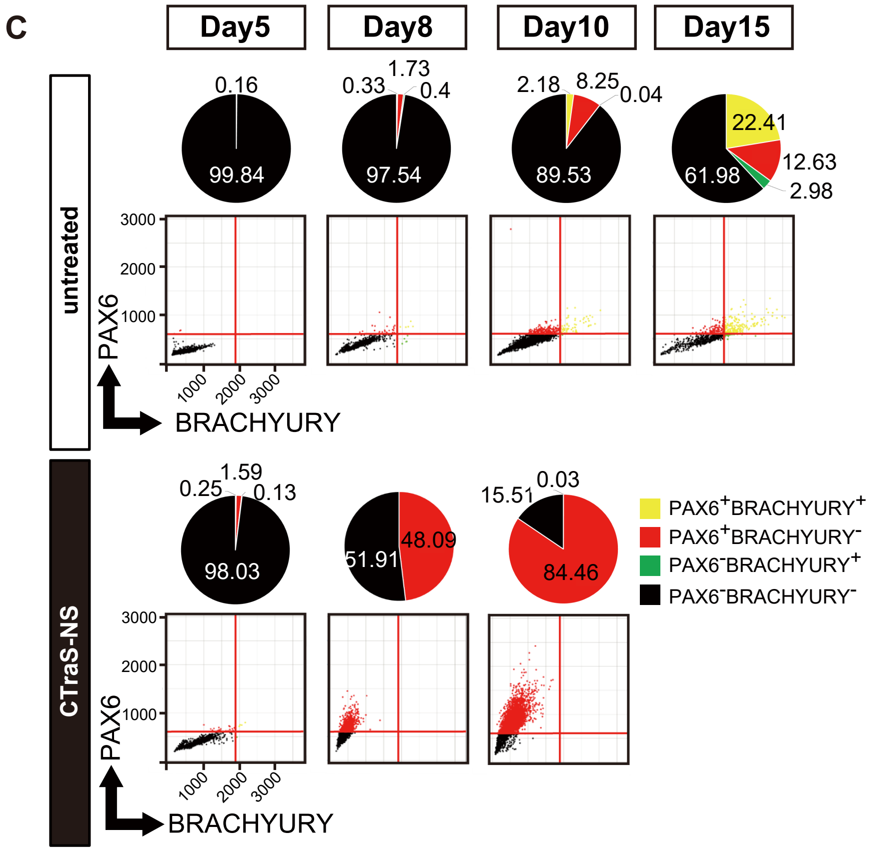

D

CTraS-NS < untreated-NS (812 genes) **Developmental Process** and **Differentiation**

GO ACCESSION	GO Term	p-value
GO:0009790|GO:0009795	embryo development	8.63E-24
GO:0007275	multicellular organism development	5.80E-23
GO:0032502	developmental process	2.08E-22
GO:0048856	anatomical structure development	1.04E-21
GO:0044767	single-organism developmental process	1.07E-21
GO:0044707	single-multicellular organism process	3.20E-20
GO:0048568	embryonic organ development	9.58E-20
GO:0048731	system development	1.30E-19
GO:0048598|GO:0048828	embryonic morphogenesis	1.70E-19
GO:0048706	embryonic skeletal system development	1.78E-19
GO:0009952	anterior/posterior pattern specification	1.90E-19
GO:0072358	cardiovascular system development	1.92E-19
GO:0072359	circulatory system development	1.92E-19
GO:0048513	animal organ development	3.14E-18
GO:0007389	pattern specification process	4.28E-18
GO:0043009	chordate embryonic development	6.61E-18
GO:0009653	anatomical structure morphogenesis	8.92E-18
GO:0001501	skeletal system development	1.01E-17
GO:0009792	embryo development ending in birth or egg hatching	1.31E-17
GO:0032501|GO:0050874	multicellular organismal process	3.03E-17

Figure S4 CTraS-hPSCs Exhibited Accelerated Lineage-Specific Differentiation toward the Ectoderm during NS Formation. (related to Figure 3)
(A) An overview of the culture protocol used in this experiment. (B) Time-dependent changes in gene expression from hPSCs to NSs via CTraS or traditional differentiation methods (n = 3 independent experiments; mean ± SEM). hPSC lines used: 201B7, WD39, and KhES1 (C) Cell population analysis of the NSs induced from untreated hPSCs and CTraS-hPSCs. NSs were single-cell dissociated and stained for the indicated markers. (D) The gene ontology analysis of the transcripts down-regulated in CTraS-NSs vs untreated NSs. hPSC line used; KA11 and eKA3

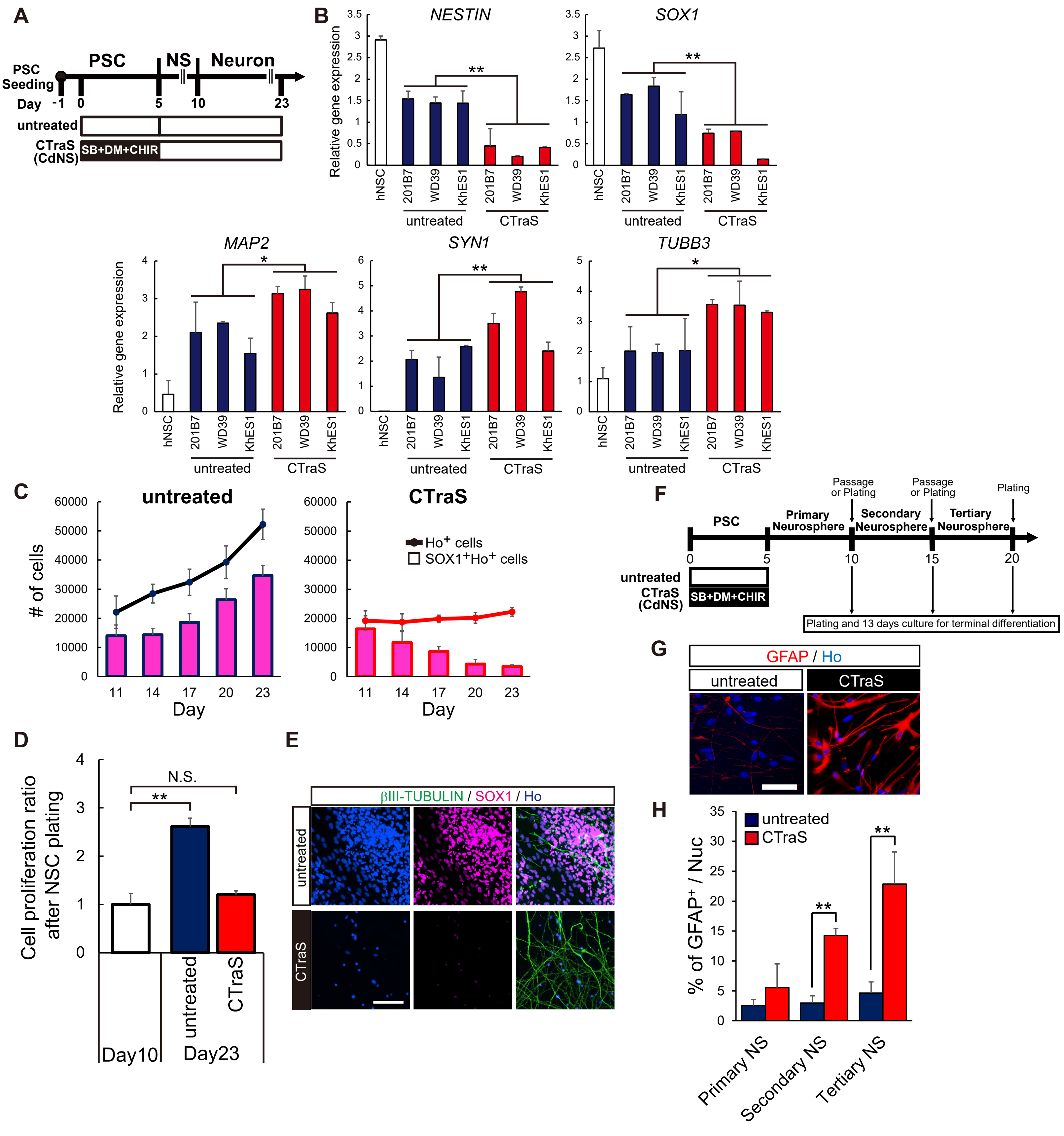

Figure S5 CdNS Accelerated the Generation of Neurons and Astrocytes almost without Residual Stem Cells. (related to Figure 4)

(A) An overview of the culture protocol used in this experiment. (B) qPCR analysis for indicated genes on three hPSC lines-derived neurons via CTraS or not (n = 3 independent experiments; mean ± SEM; *$p < 0.05$, **$p < 0.01$; Dunnett's test). (C) Residual NSC analysis, quantifying SOX1$^+$ cell ratio (n = 3 independent experiments; mean ± SEM). (D) Cell proliferation analysis after plating single-cell dissociated NSs via CTraS or not (n = 3 independent experiments; mean ± SEM; **$p < 0.01$; Dunnett's test). (E) Immunostaining of untreated-hPSC- and CTraS-hPSC-derived neurons and NSCs with antibodies raised against the indicated markers. Scale bar = 100 µm. (F) An overview of this experiment. (G) Representative images of cultures with or without terminal differentiation of tertiary neurospheres via CTraS. (H) Quatitative analysis of GFAP$^+$ cells in terminal differentiations via CTraS or not (n=3 independent experiments; mean ± SEM; **$P < 0.01$; Student's t-test). Scale bar =200 µm. hPSC lines used: 201B7, WD39, and KhES1

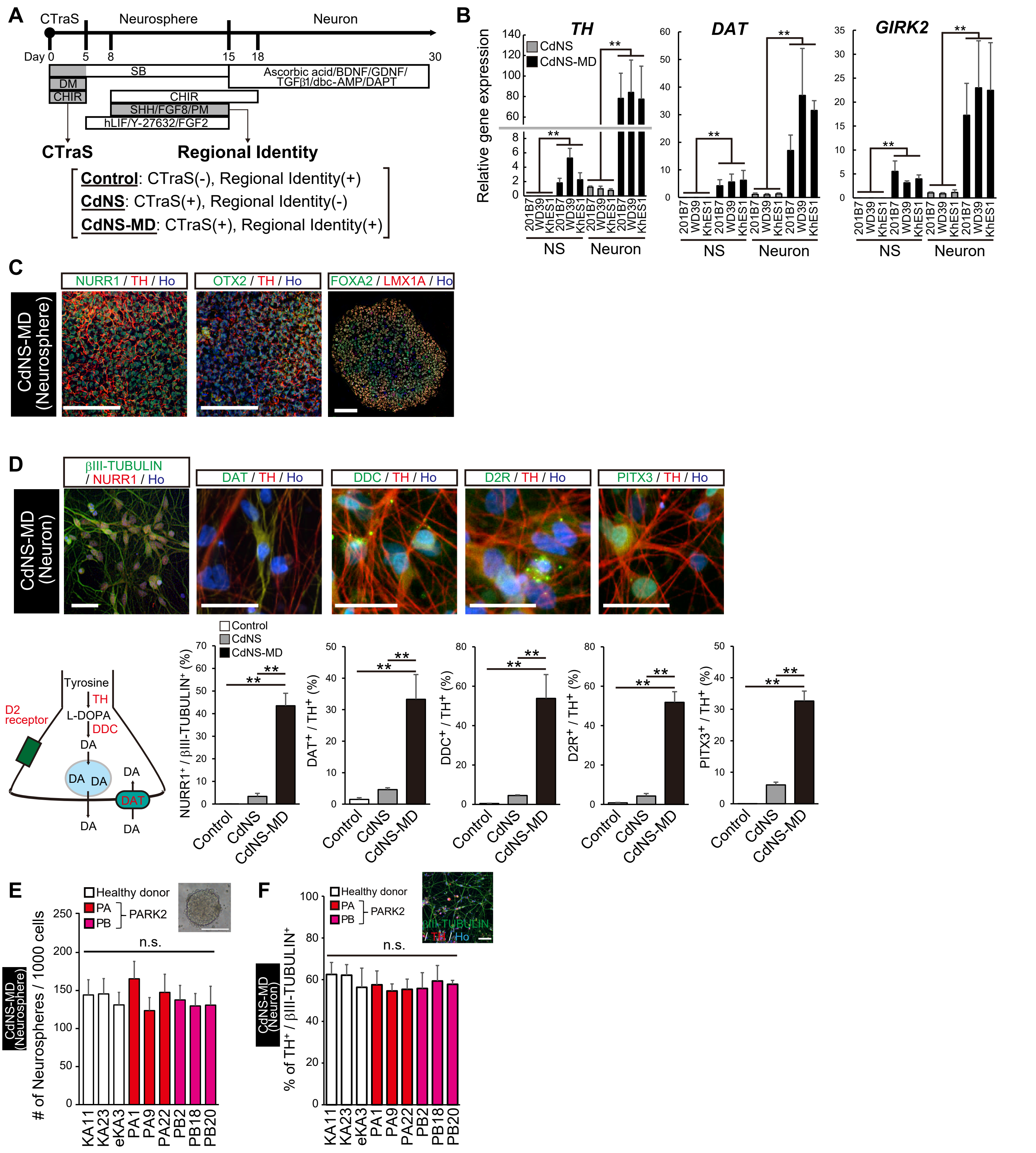

Figure S6 Differentiation of Dopaminergic Neuron via CTraS. (related to Figure 7)
(A) Overview of the culture protocol for region-specific differentiation based on CdNS. (B) qPCR analysis for the indicated expression of dopaminergic neuronal genes in NSs and neurons derived from hPSCs with the indicated protocol (n = 3 independent experiments; mean ± SEM; $^{**}P < 0.01$; Student's *t*-test). (C) Immunostaining and cell population analysis of hPSC-derived NSs at day 15 using CdNS-MD. Scale bars = 100 μm. (D) Immunostaining and cell population analysis of hPSC-derived Neurons at day 30 using indicated protocols (n = 3 independent experiments; mean ± SEM; $^{**}P < 0.01$; Dunnett's test). Scale bars = 30 μm. hPSC lines used: 201B7, WD39, and KhES1. (E) Sphere formation analysis of NSs at day 15 by using CdNS-MD (n = 3 independent experiments; mean ± SEM; n.s., not significant; ANOVA). Scale bar = 200 μm. (F) Immunostaining and cell population analysis of dopaminergic neurons at day30 differentiated from healthy donor-derived iPSCs and PARK2-iPSCs (n = 3 independent experiments; mean ± SEM; n.s., not significant; ANOVA). Scale bar = 70 μm. hPSC lines used: KA11, KA23, and eKA3 (healthy donor); PA1, PA9, and PA22 (PARK2-PA); PB2, PB18, and PB20 (PARK2-PB).

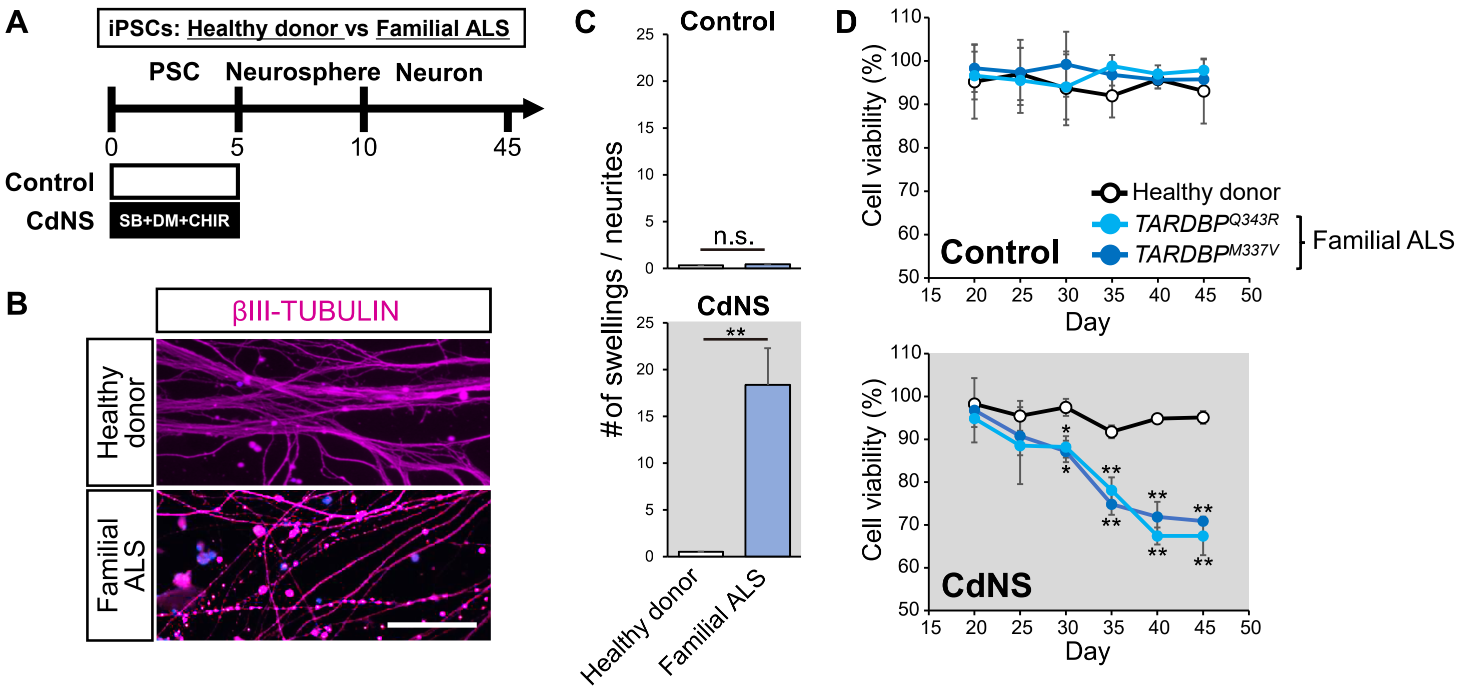

Figure S7 Acceleration of the *In Vitro* ALS Phenotype Expression by CTraS. (related to Figure 7)
(A) Overview of the culture protocol in the experiment using familial ALS patients-iPSCs. (B) Representative images of neurites. Familial ALS-iPSC derived neurons exhibited swollen neurites at day 25. Scale bar = 100 µm. (C) Quantitative data of the number of swellings in neurons at day 25 (n=3 independent experiments; mean ± SEM; ***P* < 0.01; Student's *t*-test). (D) Cell viability analysis of iPSC-derived neurons from familial ALS patients compared with those from healthy donors. hPSC lines used: 201B7, Wd39, and KA11 (healthy donor); A21412 and A21428 (Familial ALS carrying *TARDBP*^{Q343R} mutation); A3411 and A3416 (Familial ALS carrying *TARDBP*^{M337V} mutation).

Table S1 List of GO Terms
(CTraS-PSC vs untreated-PSC; a fold change differences of ± 2.0; related to Figure 2)

CTraS > untreated (1039 genes) **Biological Process** and **Molecular Function**

GO ACCESSION	GO Term	*p*-value
GO:0010033	response to organic substance	1.13E-14
GO:0071840| GO:0071841	cellular component organization or biogenesis	3.40E-14
GO:0006695	cholesterol biosynthetic process	3.69E-13
GO:1902653	secondary alcohol biosynthetic process	6.38E-13
GO:0016126	sterol biosynthetic process	7.30E-12
GO:0042221	response to chemical	7.54E-12
GO:0008203	cholesterol metabolic process	8.83E-12
GO:1902652	secondary alcohol metabolic process	2.06E-11
GO:0009987| GO:0008151| GO:0050875	cellular process	5.42E-11
GO:0048522| GO:0051242	positive regulation of cellular process	5.24E-11
GO:0032502	developmental process	6.44E-11
GO:0044767	single-organism developmental process	6.44E-11
GO:0009719	response to endogenous stimulus	9.73E-11
GO:0046165	alcohol biosynthetic process	1.11E-10
GO:0016125	sterol metabolic process	1.44E-10
GO:0048518| GO:0043119	positive regulation of biological process	1.94E-10
GO:0048856	anatomical structure development	2.88E-10
GO:0008150| GO:0000004| GO:0007582	biological process	3.14E-10
GO:0007275	multicellular organism development	3.89E-10
GO:0009966| GO:0035466	regulation of signal transduction	1.15E-09

untreated > CTraS (1054 genes) **Biological Process** and **Molecular Function**

GO ACCESSION	GO Term	*p*-value
GO:0060968	regulation of gene silencing	2.97E-10
GO:0004556	alpha-amylase activity	4.31E-09
GO:0071294	cellular response to zinc ion	1.86E-08
GO:0016160	amylase activity	2.91E-08
GO:0060255	regulation of macromolecule metabolic process	1.83E-05
GO:0009611| GO:0002245	response to wounding	1.97E-05

Table S2 List of Primers
(related to Figure 1, 3, 4, 7, S1, S2, S4, S5, and S6)

Gene	Forward	Reverse
OCT4	TTGGGCTCGAGAAGGATGTGGT	TGCATAGTCGCTGCTTGATCGC
NANOG	TGAACCTCAGCTACAAACAG	TGGTGGTAGGAAGAGTAAAG
PAX6	ACCACACCGGTTTCCTCCTTCACA	TTGCCATGGTGAAGCTGGGCAT
NESTIN	TTCCCTCAGCTTTCAGGACCCCAA	AAGGCTGGCACAGGTGTCTCAA
SOX1	GATCAGCAAGCGCCTGGGGG	AGCAGCGTCTTGGTCTTGCGG
BRACHYURY	TGCTTCCCTGAGACCCAGTT	GATCACTTCTTTCCTTTGCATCAAG
SOX17	TGTCCCAAAACAGCTTCCTC	TCACCCTTTTCGAGGATGAG
TUBB3	ATTTCATCTTTGGTCAGAGTGGGGC	TGCAGGCAGTCGCAGTTTTCAC
MAP2	GGATCAACGGAGAGCTGAC	TCAGGACTGCTACAGCCTCA
MAPT-Tau	CCTCTCCCGTCCTCGCCTCTG	GGGTCAGCCATCCTGGTTCA
NCAM	ATGGAAACTCTATTAAAGTGAACCTG	TAGACCTCATACTCAGCATTCCAGT
NSE	GGAGAACAGTGAAGCCTTGG	GGTCAAATGGGTCCTCAATG
SYN1	TTGCCCAGATGGTTCGACTG	TGTCAACCTTGACCTTGCCC
PSD95-DLG4	TCACAACCTCTTATTCCCAGCA	CATGGCTGTGGGGTAGTCG
SATB2	TCTCCCCCTCAGTTATGTGAC	AGGCAAGTCTTCCAACTTTGAA
TBR1	GCCTTTCTCCTTCTATCATGCTC	GTCAGTGGTCGAGATAATGGGA
CTIP2	TGGGTGCCTGCTATGACAAG	GGCTCGGACACTTTCCTGAG
VGLUT1	TACACGGCTCCTTTTTCTGG	CTGAGGGGATCAGCATGTTT
GRIA1	GGGGAGGTGATTCCAAGGAC	CCAGTTACAATCCCGTGGCT
GRIA2	TGGTACGACAAAGGAGAGTGC	ACCAGCATTGCCAAACCAAG
GRIN1	AGGAACCCCTCGGACAAGTT	CCGCACTCTCGTAGTTGTG
GRIN2A	CTTGCTTCAGTTTGTGGGTG	AGCCAGCATGTAGAATACGC
GRIN2B	TCCGTCTAGAGGTTTGGCTTC	ACACCAACCAGAACTTGGGAG
GAD65	CTGCTCCAGTCTCCAAAGCC	CCGTGAACTTCTGAGCCACT
GAD67	CGAGGACTCTGGACAGTAGAGG	GATCTTGAGCCCCAGTTTTCTG
TH	TCATCACCTGGTCACCAAGTT	GGTCGCCGTGCCTGTACT
DAT	TGAGCTCTTCACGCTCTTCA	CACCATAGAACCAGGCCACT
ISLET1	AGCAGCCCAATGACAAAACT	CTGAAAAATTGACCAGTTGCTG
HB9	GTCCACCGCGGGCATGATCC	TCTTCACCTGGGTCTCGGTGAGC
GFAP	CTGCTCAATGTCAAGCTGG	AATGGTGATCCGGTTCTCC
GIRK2	CTGGAAATTGTGGTCATC	GGTCTCATAGGTCTCATG
ACTB	TGAAGTGTGACGTGGACATC	GGAGGAGCAATGATCTTGAT

Table S3 List of Antibodies and Dilutions
(related to Figure 2, 3, 4, 5, 6, 7, S1, S5, S6, and S7)

Antibody	Dilution	Source	Catalogue number	Location
TRA-1-60	1:1000	Millipore	MAB4360	Billerica, MA, USA
SSEA4	1:1000	Abcam	ab16287	Cambridge, MA, USA
PAX6	1:500	BioLegend	901301	San Diego, CA, USA
NESTIN	1:500	described previously, refs. (Iwanami et al., 2005; Kobayashi et al., 2012; Nori et al., 2011)		
SOX1	1:500	R&D Systems	AF3369	Minneapolis, MN, USA
BRACHYURY	1:200	R&D Systems	AF2085	Minneapolis, MN, USA
SOX17	1:500	R&D Systems	AF1924	Minneapolis, MN, USA
AFP	1:200	R&D Systems	MAB1368	Minneapolis, MN, USA
αSMA	1:500	Sigma-Aldrich	A2547	St Louis, MI, USA
βIII-tubulin	1:1000	Sigma-Aldrich	T8660	St Louis, MI, USA
MAP2	1:500	Sigma-Aldrich	M4403	St Louis, MI, USA
Synapsin-1	1:500	Synaptic Systems	106103	Goettingen, Germany
TH	1:500	Millipore	AB152	Billerica, MA, USA
Complex III-Core I	1:200	Thermo Fisher Scientific	459140	Waltham, MA, USA
Phosphorylated α-synuclein	1:1000	Wako	014-20281	Osaka, Japan
Cleaved caspase-3	1:500	Cell Signaling Technology	9661	Danvers, MA, USA
DAT	1:200	Abcam	ab5990	Cambridge, MA, USA
FOXA2	1:500	R&D Systems	AF2400	Minneapolis, MN, USA
LMX1A	1:100	Abcam	Ab139726	Cambridge, MA, USA
NURR1	1:200	Santa Cruz Biotechnology	sc-991	Dallas, TX, USA
PITX3	1:200	Millipore	AB5722	Billerica, MA, USA
DDC	1:100	R&D Systems	AF3564	Minneapolis, MN, USA
D2R	1:1000	Abcam	ab21218	Cambridge, MA, USA
GFAP	1:4000	DAKO	Z033401	Glostrup, DK, USA
FOXG1	1:500	Abcam	MAB1585	Cambridge, MA, USA
OTX2	1:100	R&D Systems	AF1979	Minneapolis, MN, USA
EN1	1:100	Abcam	ab117549	Cambridge, MA, USA
HOXB4	1:100	DSHB	I12	Iowa City, IA, USA
PAX7	1:100	Abcam	ab34360	Cambridge, MA, USA
NKX2.2	1:100	DSHB	74.5A5	Iowa City, IA, USA

SUPPLEMENTAL EXPERIMENTAL PROCEDURES

Neural Induction *In Vitro*

For *in vitro* differentiation, hPSCs were cultured in standard hESC medium either alone or supplemented with 3 μM SB431542 (Tocris Bioscience), 3 μM dorsomorphin (Sigma-Aldrich) and 3 μM CHIR99021 (Stemgent) for 5 days. The medium was changed daily. On day 5, hPSC colonies were detached from the feeder layers using a dissociation solution (0.25% trypsin, 100 μg/ml collagenase IV (Invitrogen), 1 mM $CaCl_2$, and 20% KSR) and were enzymatically dissociated into single cells using TrypLE Select (Life Technologies). The dissociated cells were cultured in suspension at a density of 1×10^5 cells/ml in Ultra-Low attachment culture dishes (Greiner) in proliferation medium consisting of serum-free medium (media hormone mix; MHM) supplemented with 2% B27 supplement (Invitrogen), 20 ng/ml FGF, 10 ng/ml hLIF (Millipore), 2 μM SB431542 and 3 μM CHIR99021 in a hypoxic and humidified atmosphere (4% O_2, 5% CO_2). The medium was changed every 2-3 days for approximately 3-7 days to induce the formation of NSs.

To differentiate neuronal cells, dissociated NSs were plated onto either a 96-well plate or coverslips 10 mm in diameter coated with poly-L-ornithine (PO) and growth-factor-reduced Matrigel (50x dilution, thin coated; Invitrogen). These cells were cultured in differentiation medium consisting of MHM supplemented with 2% B27 supplement (Thermo Fisher), 10 ng/ml rhBDNF (R&D Systems), 10 ng/ml rhGDNF (R&D Systems), 0.2 mM ascorbic acid (Sigma-Aldrich) and 2 μM DAPT (Sigma-Aldrich) for 10-15 days in a humidified atmosphere containing 5% CO_2. Half of the medium was changed every 2–3 days.

Immunocytochemistry

Cells were fixed in phosphate-buffered saline (PBS) containing 4% paraformaldehyde (PFA) for 30 min at room temperature. Thereafter, all the cells were blocked with 5% FBS and Triton X-100 and incubated with the primary antibodies described in Supplementary Table 1. The cells were then rinsed with PBS and incubated with species-specific Alexa Fluor 488-, Alexa Fluor 555-, or Alexa Fluor 647-conjugated secondary antibodies (1:500; Invitrogen), which was followed by the addition of Hoechst 33258 (0.5 μg/mL; Sigma-Aldrich) to counterstain the nuclei. The images were obtained using an Axioplan2 (Carl Zeiss AG), LSM-710 (Carl Zeiss AG), BZ9000 (Keyence), or IN Cell Analyzer 6000 (GE Healthcare).

High-Content Analysis

For the cell population assays, fluorescence intensity analysis, neural maturation analysis, and ROS production assays, stained plates were imaged on an IN Cell Analyzer 6000 high-content cellular analysis system (GE Healthcare), and a set of 5 x 5 fields were collected from each well using the 20x objective, resulting in the scoring of over 10,000 cells per well. For the mitochondrial function assay, stained plates were imaged on an IN Cell Analyzer 6000, and a set of 6 x 6 fields were collected from each well using the 60x objective, resulting in the scoring of over 9,000 cells per well. Analysis (IN Cell Developer Toolbox v1.9; GE Healthcare) was conducted by identifying intact nuclei stained with Hoechst; these

nuclei were defined as traced nuclei that were larger than 50 μm^2 in surface area and with typical intensity levels that were lower than the threshold brightness of pyknotic cells. Each traced nuclear region was then expanded by 50% and cross-referenced with endodermal markers (SOX17 and AFP), mesodermal markers (BRACHYURY and αSMA), ectodermal markers (PAX6, NESTIN, and βIII-TUBULIN), an NS/PC marker (SOX1), neuronal markers (MAP2 and βIII-TUBULIN), and pluripotent markers (SSEA4 and TRA-1-60) to identify the cell type; from these images, the percentages and fluorescence intensities of these cells were calculated.

Using the above described traced images of each cell, the cell population CTraS-PSCs, CTraS-NSs, and CTraS-Neurons; the fluorescence intensity of the markers of all three germ layers in the CTraS-PSC and EB differentiation cultures; the CellROX fluorescence intensity of the neurons; the cleaved caspase-3 intensity and the area of the TH intensities in neurons were analyzed. By setting the areas on βIII-TUBULIN$^+$ cells, the number of synapsin1$^+$ puncta in neurons was analyzed.

Neurosphere Formation Assay

Neurosphere (NS) cultures were fixed and immunostained by SOX1 1 hour after plating the dispersed NS cultures, which were cultivated for 12 hours (Day 5), 3 days (Day 8), 5 days (Day 10), or 10 days (Day 15) after NS induction.

The analysis (IN Cell Developer Toolbox v1.9; GE Healthcare) began by identifying intact nuclei stained with Hoechst (Ho), which were defined as traced nuclei that were larger than 50 μm^2 in surface area and with typical intensity levels that were lower than the threshold brightness of pyknotic cells. Up to 1000 of these Ho$^+$ cells were counted, and the NS formation assay was performed using this cell population. The NS size was assessed by measuring the NS diameter (> 50 μm) and surface area (> 1000 μm^2) using an IN Cell Analyzer 6000 System (GE Healthcare), and only SOX1$^+$ NSs were calculated.

Microarray Analysis

Total RNA was extracted using an RNeasy Kit (QIAGEN), and the RNA quality was assessed using an Agilent 2100 Bioanalyzer (Agilent Technologies). Total RNA (100 ng) was reverse-transcribed, labeled with biotin using a 3'IVT Express Kit (Affymetrix) and hybridized to a GeneChip® Human Genome U133 plus 2.0 Array (Affymetrix). The arrays were washed and stained using a GeneChip Fluidics Station 450 (Affymetrix) and then scanned with a GeneChip Scanner 3000 7G System (Affymetrix) according to the manufacturer's instructions. The raw probe intensity files were normalized to MAS5 and log (base2) transformed using GeneSpring GX 13.1 software (Agilent Technologies). The gene set was filtered based on the expression levels to remove genes that were not expressed in all the samples. PCA was performed using the normalized data. For the hierarchical clustering, the normalized data were calculated based on Euclidean correlations with average linkages.

The data discussed in this publication have been deposited into the NCBI Gene Expression Omnibus (GEO http://www.ncbi.nlm.nih.gov/geo/) database and are accessible using GEO Series accession number

GSE95271.

Quantitative RT-PCR

Total RNA was isolated with a RNeasy mini kit (QIAGEN) and treated with DNase I, and cDNA was prepared using a ReverTraAce qPCR RT kit (Toyobo). Quantitative RT-PCR was performed using SYBR Premix Ex Taq II (Takara Bio) on a ViiA 7 Real-Time PCR System (Life Technologies) (Supplementary Table 2).

Microelectrode Array Recording

Microelectrode array (MEA) recording was performed using a Maestro system (Axion Biosystems). To prepare the neurons for array recording, single-cell dissociated NSs were subsequently plated onto the electrode area in a Matrigel (Corning)-coated MEA plate at a density of 1.5×10^5 cells/cm^2 per well. Once the cells were attached, MHM supplemented with 2% B27 supplement (Thermo Fisher), 10 ng/ml rhBDNF (R&D Systems), 10 ng/ml rhGDNF (R&D Systems), 0.2 mM ascorbic acid (Sigma-Aldrich) and 2 μM DAPT (Sigma-Aldrich) was added to the well. The medium was subsequently changed every 2-3 days. The data were acquired using a sampling rate of 12.5 kHz and filtered using a 200-3000 Hz Butterworth bandpass filter. A detection threshold was set to +6.0 times the SD of the baseline electrode noise. The spike count files generated from the recordings were used to calculate the number of active electrodes (defined as an electrode having an average of more than 5 spikes/min) in each well, the average per-active electrode mean firing rate (MFR; spikes/min) and the standard deviation of the average per-active electrode MFR. The data from the initial 3 min in each data file were omitted to enable the activity to stabilize in the Maestro, and 10–15 min of activity was subsequently recorded.

Cell Viability Analysis

An MTT Cell Viability Assay Kit (BioAssay Systems) was used to evaluate the cell viability of hiPSC-derived neurons. In all the experiments, single-cell dissociated NSs were seeded into 96-well plates at a density of 1.5×10^5 cells/cm^2 per well. At five days after NS seeding, the MTT assay was performed every fifth day according to the manufacturer's protocol. MTT (15 μL/well) was added to the wells and incubated at 37°C for 4 h. The supernatant was removed, and 100 μL of solubilizer per well was added to dissolve the produced formazan. After shaking the plates for 10 min, the absorbance values of the wells were recorded with a microplate reader at a wavelength of 570 nm.

SUPPLEMENTAL REFERENCES

Iwanami, A., Kaneko, S., Nakamura, M., Kanemura, Y., Mori, H., Kobayashi, S., Yamasaki, M., Momoshima, S., Ishii, H., Ando, K.*, et al.* (2005). Transplantation of human neural stem cells for spinal cord injury in primates. Journal of neuroscience research *80*, 182-190.

Kobayashi, Y., Okada, Y., Itakura, G., Iwai, H., Nishimura, S., Yasuda, A., Nori, S., Hikishima, K., Konomi, T., Fujiyoshi, K.*, et al.* (2012). Pre-evaluated safe human iPSC-derived neural stem cells promote functional recovery after spinal cord injury in common marmoset without tumorigenicity. PloS one *7*, e52787.

Nori, S., Okada, Y., Yasuda, A., Tsuji, O., Takahashi, Y., Kobayashi, Y., Fujiyoshi, K., Koike, M., Uchiyama, Y., Ikeda, E.*, et al.* (2011). Grafted human-induced pluripotent stem-cell-derived neurospheres promote motor functional recovery after spinal cord injury in mice. Proceedings of the National Academy of Sciences of the United States of America *108*, 16825-16830.
