# Supplementary material for: Escape from Pluripotency via Inhibition of TGF-β/BMP and Activation of Wnt Signaling Accelerates Differentiation and Aging in hPSC Progeny Cells
Source: Stem Cell Reports. 2017 Oct 26;9(5):1675–91. doi: 10.1016/j.stemcr.2017.09.024 (PMC5831048; doi:10.1016/j.stemcr.2017.09.024)
Supplement: Document S2. Article plus Supplemental Information [file mmc2.pdf]

Stem Cell Reports
Article
OPEN ACCESS

Escape from Pluripotency via Inhibition of TGF-β/BMP and Activation of Wnt Signaling Accelerates Differentiation and Aging in hPSC Progeny Cells

Koki Fujimori,[1,2] Takuya Matsumoto,[1] Fumihiko Kisa,[1] Nobutaka Hattori,[3] Hideyuki Okano,[1,*] and Wado Akamatsu[1,4,*]
[1]Department of Physiology, Keio University School of Medicine, Shinjuku-ku, Tokyo 160-8582, Japan
[2]Research Fellow of Japan Society for the Promotion of Science, Chiyoda-ku, Tokyo 102-0083, Japan
[3]Department of Neurology, Juntendo University School of Medicine, Bunkyo-ku, Tokyo 113-8431, Japan
[4]Center for Genomic and Regenerative Medicine, Juntendo University School of Medicine, Bunkyo-ku, Tokyo 113-8431, Japan
*Correspondence: hidokano@a2.keio.jp (H.O.), awado@juntendo.ac.jp (W.A.)
https://doi.org/10.1016/j.stemcr.2017.09.024

SUMMARY

Human pluripotent stem cells (hPSCs) represent a potentially valuable cell source for applications in cell replacement therapy, drug development, and disease modeling. For all these uses, it is necessary to develop reproducible and robust protocols for differentiation into desired cell types. However, differentiation protocols remain unstable and inefficient, which makes minimizing the differentiation variance among hPSC lines and obtaining purified terminally differentiated cells extremely time consuming. Here, we report a simple treatment with three small molecules—SB431542, dorsomorphine, and CHIR99021—that enhanced hPSC differentiation into three germ layers with a chemically transitional embryoid-body-like state (CTraS). Induction of CTraS reduced the innate differentiation propensities of hPSCs (even unfavorably differentiated hPSCs) and shifted their differentiation into terminally differentiated cells, particularly neurons. In addition, CTraS induction accelerated *in vitro* pathological expression concurrently with neural maturation. Thus, CTraS can promote the latent potential of hPSCs for differentiation and potentially expand the utility and applicability of hPSCs.

INTRODUCTION

Human pluripotent stem cells (hPSCs) have been in development for applications in cell replacement therapy (Okano et al., 2013; Tabar and Studer, 2014), drug discovery (Imamura et al., 2017), and hPSC disease modeling (Ichiyanagi et al., 2016; Imaizumi et al., 2015; Matsumoto et al., 2016) using patient-derived induced pluripotent stem cells (iPSCs). Although the development of reproducible and robust protocols for the differentiation into desired cell types will accelerate progress in these fields, differentiation protocols for several types of neural cells remain unstable and inefficient to obtain terminally differentiated cells without any specialized purification techniques (Matsumoto et al., 2016). In addition, individual hPSC lines are predisposed to differentiating into specific cell lineages, which may be influenced by the source cell type, donor, and reprogramming method (Kim et al., 2010, 2011; Osafune et al., 2008; Polo et al., 2010). To overcome this concern in neural disease modeling, we recently developed the direct neurosphere (dNS) conversion method (Fujimori et al., 2016; Matsumoto et al., 2016). Previous studies of early *Xenopus* development showed that dissociation of animal cap cells results in neuralization, presumably due to the loss of extracellular factors (Wilson and Hemmati-Brivanlou, 1995). BMP-4 and other extracellular signaling molecules present in the animal cap are known to repress neural development (Piccolo et al., 1996). Relevant to this, in our dNS method, PSCs are placed in a low-density floating culture to exclude all exogenous signals, including BMPs, to achieve efficient neural differentiation consistent with the default mechanism in neural fate specification exhibited by mouse embryonic stem cells (ESCs) (Nori et al., 2011), *Xenopus*, and iPSCs derived from T cells (Fujimori et al., 2016; Matsumoto et al., 2016).

Although we used a neural differentiation protocol involving embryoid body (EB) formation to prepare neural cells from hPSCs for use in pre-clinical studies in spinal cord injury(Kobayashi et al., 2012; Nori et al., 2011), the EB-based protocol was time consuming (~2 months to induce neurospheres) and was unable to efficiently differentiate T cell-derived iPSCs (TiPSCs) (Matsumoto et al., 2016). Upon implementing EB-based neural differentiation protocols, several differentiation-resistant hPSC lines exhibited low-level expression of ectodermal markers at the EB stage and poor neurosphere (NS) formation from the dissociated EBs (Matsumoto et al., 2016). However, during the process of lineage-specific differentiation from PSCs, EB formation has been widely used to initiate spontaneous differentiation toward the three germ lineages, as it is a relatively simple method for obtaining lineage-committed cells in the mesodermal and endodermal lineages (Ng et al., 2005; Ogawa et al., 2013; Yang et al., 2008). During the EB formation process in high-density floating cultures in the presence of serum, non-committed PSCs receive various signals under 3D-culture conditions. We hypothesized that the presence of diverse extracellular signals interferes with efficient neural differentiation and that the various differentiation propensities of the PSC clones leads to differences in the cell distribution within

This is an open access article under the CC BY license (http://creativecommons.org/licenses/by/4.0/).
CrossMark

the EBs. However, EBs provide a highly suitable environment for the maturation of committed cells.

Compared with cells cultured in 3D *in vitro* conditions, monolayer 2D-cultured cells are directly and homogeneously affected by exogenous factors in the culture medium. It has been reported that several small molecules can enhance and accelerate lineage-specific differentiation from hPSCs (Li et al., 2013). In the present study, we focused on the effects of SB431542 (SB), dorsomorphin (DM), and CHIR99021 (CHIR). SB has been implicated in efficient neural conversion of human ESCs (hESCs) and hiPSCs via inhibition of SMAD signaling in combination with Noggin activity (Chambers et al., 2012). Noggin, an inhibitor of BMP signaling, can be replaced by DM, which only enhances neural induction (Di-Gregorio et al., 2007). CHIR is an inhibitor of glycogen synthase kinase 3 (GSK3) and activates the canonical Wnt signaling pathway (Ring et al., 2003). Although the precise mechanism of Wnt signaling remains controversial, CHIR is often used to drive the induction of endodermal and/or mesendodermal specification, especially during early development (Clevers et al., 2014). Since these small molecules enhance different forms of germ-layer-specific differentiation through their effects on each pathway, we hypothesized that induced differentiation in a 2D culture environment by a defined combination of chemicals could give rise to cells at the transitional differentiation state that would be committed to all three germ layers in an unbiased manner.

In this study, we evaluated the effect of three small molecules on 2D cultures of undifferentiated hPSCs to induce intermediate progenitor cells. In addition, by differentiating these chemically induced cells (chemically transitional EB-like state [CTraS]) using conventional differentiation protocols, we demonstrated the potential of CTraS cells as core precursor cells for lineage-specific differentiation and as models of disease, particularly neurological disorders. In addition, CTraS induction is applicable to a wide range of hPSCs in that nearly all types of hPSCs can be induced to differentiate into neuronal cells without hPSC colony selection. Thus, CTraS could serve as a core intermediate progenitor to induce the differentiation of hPSCs irrespective of their innate differentiation propensities.

RESULTS

Evaluation of Small Molecules to Accelerate the Differentiation of All Three Germ Layers from hPSCs

Optimal concentrations for each SB, DM, and CHIR treatment to hPSCs were determined based on the results of PSC colony morphologies and the expression of each germ-layer marker (Figures S1A and S1B). Undifferentiated hPSCs were treated with SB, DM, CHIR or a combination of the three compounds for 5 days on a feeder in the presence of fibroblast growth factor 2 (FGF-2) as shown in Figure 1A. These small molecules clearly affected the morphology of the hPSC colonies, and the diameter of colonies was significantly smaller than that of the untreated group, especially the SB + DM + CHIR group (Figures 1B and S1C–S1E). Combined treatment with all three inhibitors induced a significant decrease of the expression of pluripotent markers and an increase in the expression of germ-layer markers as well as the number of floating EBs (Figures 1C and 1D). These results indicate that treating hPSCs with SB, DM, and CHIR can cause the differentiation of these PSC colonies into EB-like 2D colonies on feeder layers.

To determine the optimal treatment duration with these small molecules, we next evaluated the hPSC morphology and the changes in the expression of pluripotent markers and germ-layer markers (Figures 1E–1G and S1H–S1J) and concluded that a 5-day treatment with the small molecules was best suited for inducting the differentiated state of hPSCs. The other two hPSC lines also showed similar changes after a 5-day treatment (Figures S2A and S2B). In addition, the differentiation-promoting effect of these three agents was more significant at the PSC stage compared with the later differentiation stage (Figures S2C–S2F). These results indicate that 5-day administration of SB, DM, and CHIR efficiently converts undifferentiated hPSCs into an EB-like state of differentiation, hereafter referred to as CTraS.

Synergistic Inhibition of the GSK3, TGF-β, and BMP Signaling Pathways Enhanced the Endodermal, Mesodermal, and Ectodermal Differentiation of hPSCs

To explore differences in the signaling pathways affected by CTraS induction, we evaluated the global gene expression profiles in CTraS PSCs, untreated PSCs, and EBs. Hierarchical clustering analysis revealed that CTraS PSCs were grouped more closely with EBs than with untreated hPSCs (Figure 2A). Based on the hierarchical clustering, we extracted the gene set with different expression pattern in CTraS PSCs compared with EB and untreated PSCs. Pathway analysis using these gene sets demonstrated that cholesterol biosynthesis and its related pathways were remarkably promoted in CTraS induction (Figure S3). Next, we prepared a list of CTraS-regulated genes by selecting gene expression with fold changes >2.0. We analyzed the Gene Ontology (GO) terms of the genes (Table S1) and grouped them into three major classes; Biological Process, Molecular Function, and Cellular Component. We focused on the "Developmental Process and Differentiation" terms within in the "Biological Process" and "Molecular Function" groups to evaluate the effects of CTraS induction. Although there were no terms related to

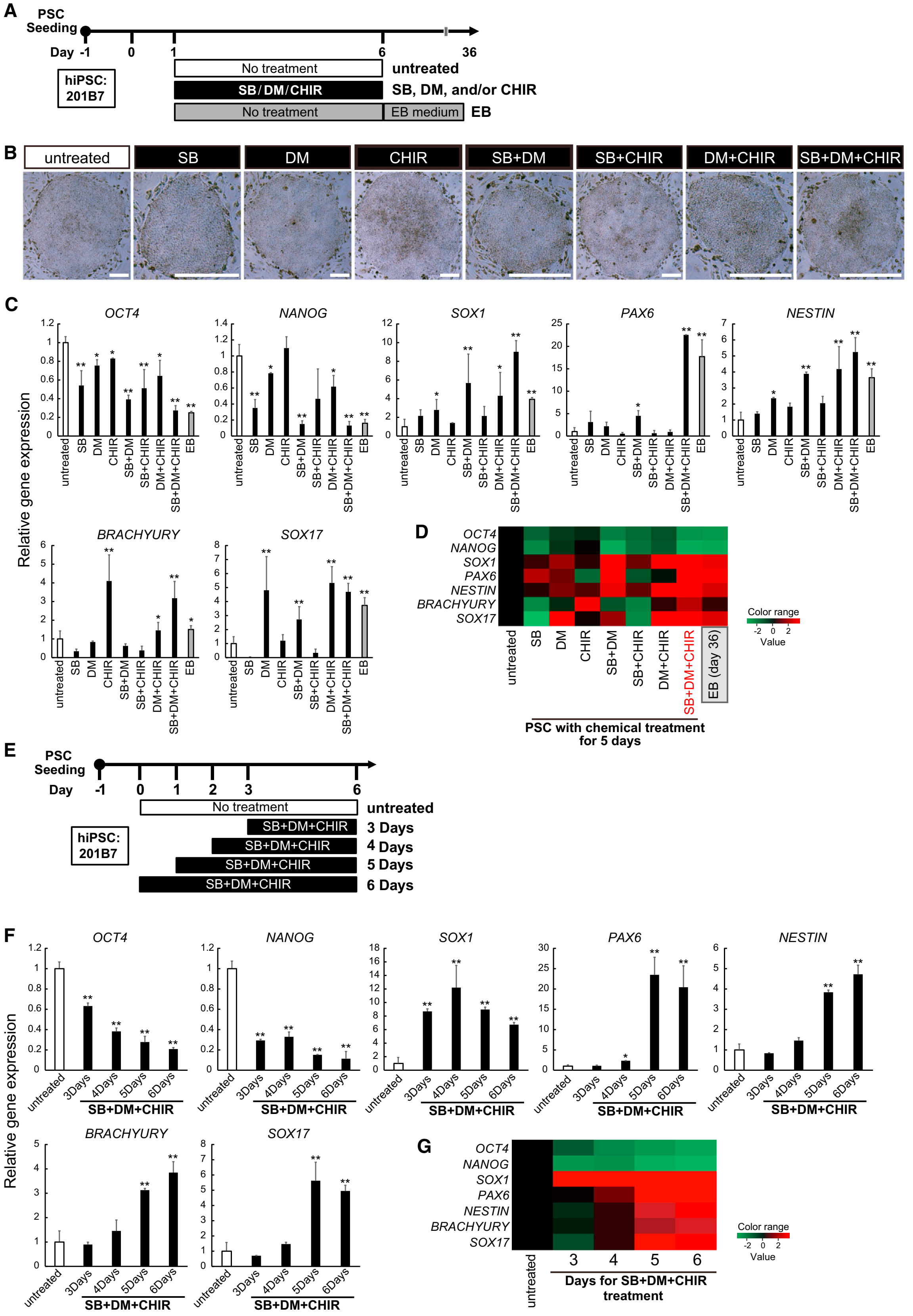

(legend on next page)

“Developmental Process and Differentiation” in the downregulated group, 15.2% of genes in the upregulated group contained development-related terms (Figure 2B). In addition, most terms in the upregulated group were not related to lineage specification (Table S1). These results also suggest that CTraS induction differentiates hPSCs into three germ layers. Pathway analysis identified multiple signaling pathways related to CTraS induction, including “cell cycle,” “apoptosis modulation and signaling,” and “senescence and autophagy,” indicating the enhancing effects on aging-related signaling at least in certain cell subpopulations (Figure 2C).

To evaluate how individual cells within CTraS-PSC colonies were altered by inhibitor treatment, we stained either colonies or dissociated single cells from untreated PSCs and CTraS PSCs for pluripotent markers and markers for all three germ layers. The intensities and frequencies of the pluripotent markers TRA-1-60 and SSEA4 were significantly decreased in CTraS colonies (Figures S1F and S1G). In addition, CTraS PSCs showed significant expression of markers representing all three germ layers (Figure 2D). Cell population analysis also revealed that CTraS induction decreased the number of undifferentiated cells and increased the number of differentiated cells in all the germ layers (Figures 2E and 2F). Next, to evaluate the effect of CTraS on differentiation ability, we formed EBs from CTraS PSCs and untreated PSCs. Although the morphologies seemed similar in both CTraS EBs and untreated EBs, the frequencies of the differentiation markers were significantly increased in CTraS-EB-derived cells (Figures 2G and 2H). These data indicate that the addition of SB, DM, and CHIR to hPSCs strongly enhances lineage-specific differentiation, resulting in the generation of cell clusters containing endodermal, mesodermal, and ectodermal cells on the feeder layers.

CTraS Induction Accelerated Subsequent Differentiation with Lineage Specificity

Next, we evaluated the differentiation propensity of CTraS-derived cells using dNS, which can differentiate most hiPSC clones (even those derived from blood cells) (Matsumoto et al., 2016). To adjust the induction periods, control cells were cultured for an additional 5 days in the NS formation (Figure 3A). We observed that the number of SOX1^{+} NS were markedly increased in the CTraS group at day 10 after NS induction in all the fractions compared with the control cells at day 15 (Figures 3B–3D).

PSC-derived NSs typically contain heterogeneous cell progeny containing original neural stem cells (NSCs), including NSCs themselves and their progeny (Hawes and Pera, 2006). To evaluate the effects of CTraS on the cell population in NSs, we quantified the expression of pluripotent markers and markers for all three germ layers. Although CTraS induction represented the time-dependent upregulation of the markers for all three germ layers, the expression of mesoderm and endoderm markers were significantly decreased during NS formation (Figures 3F–3G, S4A, and S4B). In addition, NSs from CTraS PSCs at day 10 significantly increased the expression of the neural marker *TUBB3*, and also expressed βIII-TUBULIN in the protein level (Figures 3E and 3F). A cell population analysis also clarified that the ratio of cells committed to the ectodermal lineage was apparently increased in CTraS-derived NSs at all time points measured (Figure S4C). By immunocytochemical analysis of formed NSs using representative markers in the anteroposterior (A-P) axis and dorsoventral (D-V) axis, we clarified that the cell population constituting CTraS NSs retained the region specificity on the dorsal side around the midbrain/hindbrain without significant difference from untreated NSs (Figure 3H).

To explore the detailed differences in neural differentiation between CTraS PSCs and untreated PSCs, we evaluated the global transcriptional profiles in NSs derived from these cells. Principal component analysis demonstrated that the gene expression patterns of NSs were significantly affected by CTraS induction (Figure 3I). GO analysis showed that in the category of “Developmental Process and Differentiation,” most of the extracted GO terms in the CTraS upregulated group were related to neural lineage, while those in the CTraS downregulated group were related to other lineages (Figures 3J, 3K, and S4D). In addition, in the “Biological Process and Molecular Function” category, the top

Figure 1. Evaluation of Small Molecules for Enhancing hPSC Differentiation
(A) Schematic representation of experiments for screening combinations of hPSC differentiation enhancers.
(B) Representative images of SB-, DM-, and/or CHIR-treated hPSCs. Scale bars, 200 μm.
(C) qPCR analysis of the indicated genes in hiPSCs cultured under the indicated conditions for 5 days compared with 36-day EB cultures (n = 3 independent experiments; mean ± SEM; *p < 0.05, **p < 0.01; versus untreated; Dunnett’s test).
(D) Heatmap summary of the qPCR analysis shown in (C).
(E) Schematic of experiments for the time course of the treatment with the three small molecules (SB, DM, and CHIR).
(F) qPCR analysis for the indicated genes in hiPSCs cultured with SB, DM, and CHIR for the indicated days (n = 3 independent experiments; mean ± SEM; *p < 0.05, **p < 0.01; versus untreated; Dunnett’s test).
(G) Heatmap summary of the qPCR analysis shown in (F).
hPSC line used: 201B7. See also Figures S1 and S2.

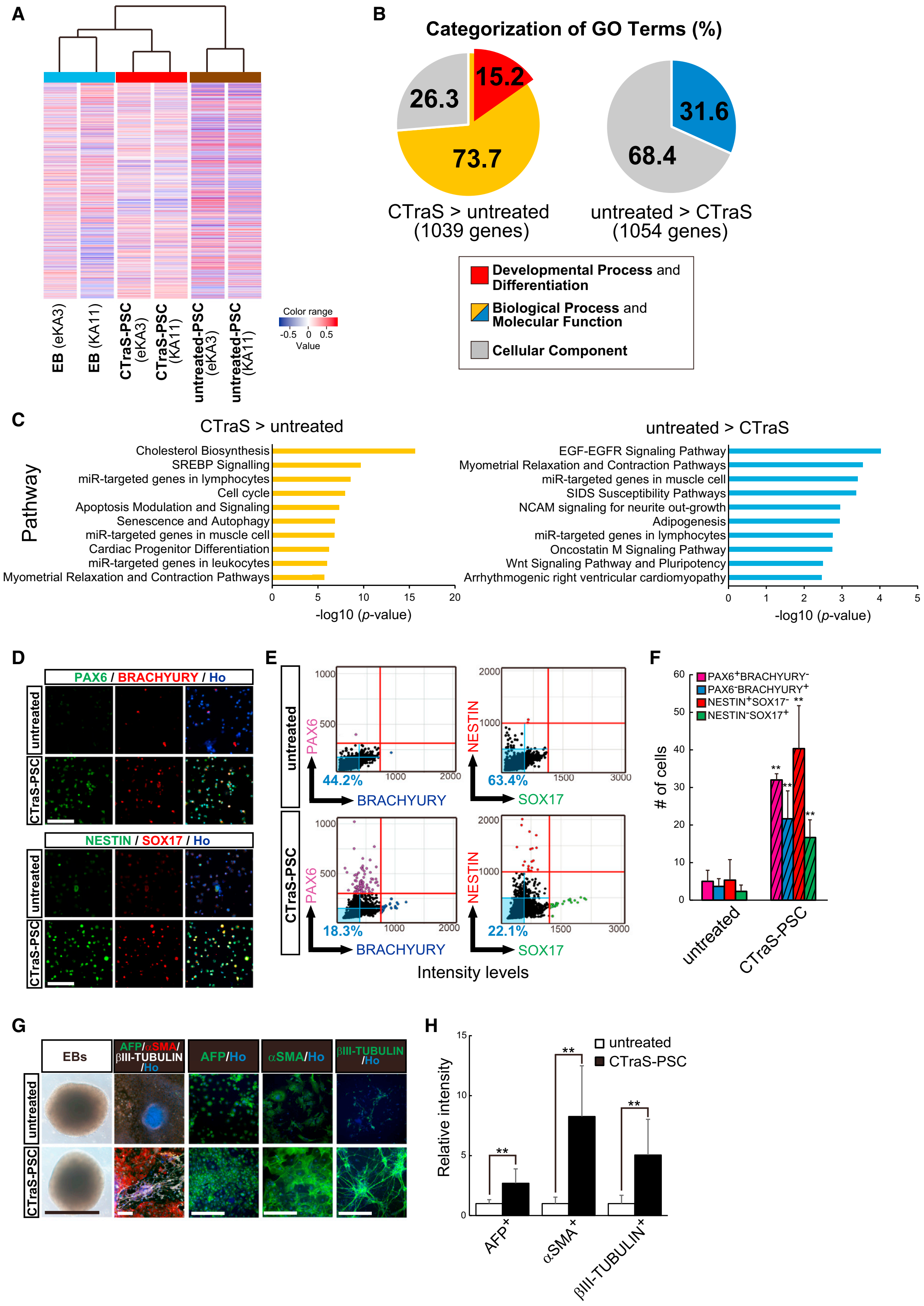

(legend on next page)

20 GO terms associated with CTraS upregulation contained 18 development-related terms, one-third of which were related to neural development (Figure 3K). These data support our cell population analysis data and strongly suggest that escape from pluripotency via CTraS induction not only accelerates subsequent differentiation but also enhances the lineage-specific differentiation depending on the surrounding environment.

Efficient Generation of Functional Neurons Using Direct Neurosphere Conversion via CTraS

We next examined whether CTraS NSs enhanced the terminal differentiation of neural cells (Figure 4A). After 13 days, differentiated cells from CTraS NSs showed increased expression of various neuronal and astrocyte markers and downregulated levels of NSC markers, pluripotent markers, mesoderm markers, and endoderm markers, while the expression of cortical neuron markers showed no significant difference between CTraS neurons and untreated neurons (Figures 4B, S5A, and S5B). Immunocytochemical analysis also revealed that differentiated CTraS NSs were mostly terminal differentiated cells, meanwhile those of untreated NSs were mostly NSCs with high proliferation potency (Figures 4C–4F and S5C–S5E). In addition, differentiation efficiency into astrocytes, which required differentiation and maturation at the NS stage, was also promoted by CTraS induction (Figures S5F–S5H). These data indicate that NSs derived from CTraS PSCs were rapidly differentiated into neuron and glia, resembling those found in the relatively posterior region between the midbrain and hindbrain and that the differentiated cell population had few residual stem cells.

We next sought to evaluate maturation of neural cells using the mature neuronal marker SYNAPSIN1 (Valtorta et al., 2011). Immunocytochemical analysis revealed that the number of SYNAPSIN1$^+$ puncta on neurons was significantly increased in CTraS-NS-derived neurons (Figures 4G and 4H). Moreover, electrophysiological analysis using a microelectrode array (MEA) recording system demonstrated that CTraS-NS-derived neurons showed frequent spontaneous firing at 20 per day (Figures 4I–4K) while control cells never exhibited this activity. These data indicated that dNS-based neuronal differentiation protocols via CTraS (CTraS-dNS; CdNS) can efficiently accelerate neuronal differentiation into mature functional neurons.

Neural Induction from Differentiation-Resistant ESC Lines and 30 Newly Established TiPSC Lines via CTraS

We sought to determine whether CTraS induction would improve the efficiency of neural differentiation of differentiation-resistant PSC clones. We evaluated four ESC lines (KhES 2–5), which exhibit insufficient neural differentiation via dNS. Using the CTraS induction protocol shown in Figure 5A, all four KhESC lines presented significantly increased expression of the markers of all three germ layers (Figure 5B). Although their differentiation patterns were slightly maintained in EBs even after CTraS induction, this treatment clearly reduced the differences among all four KhESC lines (Figures 5C and 5D) and accelerated their differentiation into all three germ layers (Figures 5C and 5E).

Then, to evaluate the effect of CTraS on NS formation and the subsequent neural differentiation of each KhESC line, we applied CdNS. Although all four KhESC lines tested showed poor NS formation and neural differentiation, CdNS significantly increased the number of NSs and showed highly efficient neural differentiation in 23 cultures from hPSCs (Figures 5F–5J). Immunocytochemical analysis revealed that the number of SYNAPSIN1$^+$ puncta was also significantly increased in neurons derived from CTraS NSs in all four KhESC lines (Figure 5K).

Next, we examined whether CTraS induction could improve the propensity for diverse differentiation of iPSC clones. Using Sendai virus, we established TiPSC clones

Figure 2. Synergistic Inhibition of the GSK3, TGF-β, and BMP Signaling Pathways Enhanced the Differentiation State of the hPSCs
(A) Hierarchical clustering analysis of the global gene expression profiles of untreated PSCs, CTraS PSCs, and EBs.
(B) Gene Ontology analysis of transcripts that were up- and downregulated in CTraS PSCs compared with the transcript levels in untreated PSCs (a fold change difference of ±2.0).
(C) Top 20 pathways associated with the genes that were differentially expressed in CTraS PSCs compared with those in untreated PSCs (a fold change difference of ±2.0). hPSC lines used, KA11 and eKA3.
(D) Immunostaining of single-cell dissociated untreated PSCs and SB + DM + CHIR-treated PSCs for the indicated tridermal lineage markers. Scale bars, 100 μm.
(E and F) Cell population and analysis using single-cell dissociated untreated PSCs and CTraS PSCs stained for the indicated markers (n = 3 independent experiments; mean ± SEM; $**p < 0.01$; Student's t test). Intensity of markers (E) and numbers of cells (F) are shown.
(G) Immunocytochemistry for the *in vitro* germ-layer assay and a representative image of EBs derived from untreated and CTraS PSCs. Scale bars, 200 μm.
(H) Relative intensity of the indicated tridermal lineage markers in EBs induced from untreated or CTraS PSCs (n = 3 independent experiments; mean ± SEM; $**p < 0.01$; Student's t test). hPSC lines used: 201B7, WD39, and KhES1.
See also Figures S2 and S3.

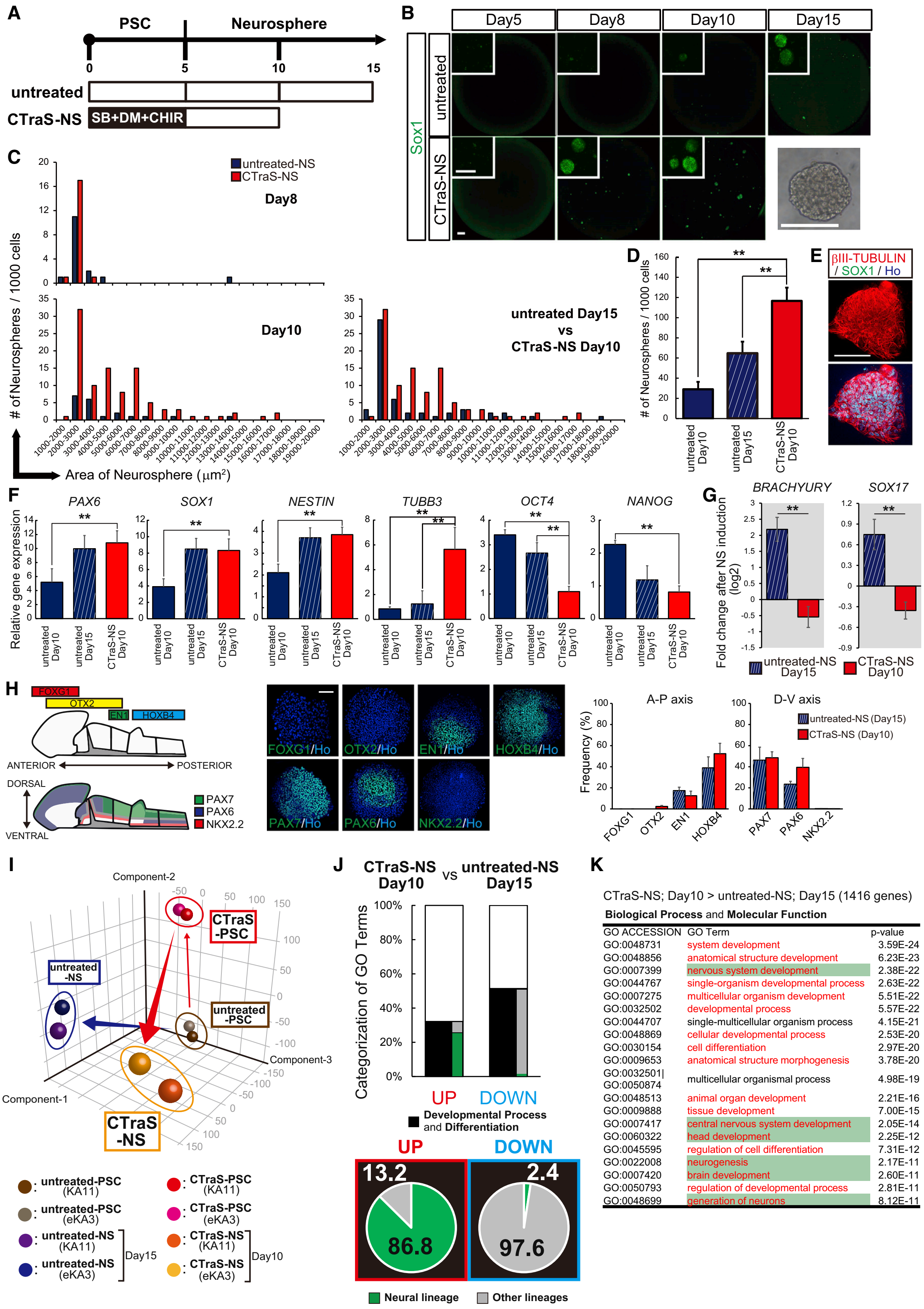

(legend on next page)

from T cells obtained from a healthy donor. We randomly picked 30 independent colonies with typical hiPSC morphologies (SeV-TiPSC 1–30; 30 cell lines total). These clones were expanded and differentiated into neurons via NSCs using the CdNS protocol as shown in Figure 6A. Twenty-three days after neural differentiation, βIII-TUBULIN⁺ neurons were observed in nine SeV-TiPSC clones differentiated without CTraS induction. In contrast, 28 clones gave rise to βIII-TUBULIN⁺ neurons via CTraS induction over the same period (Figures 6B and 6C). The percentage of βIII-TUBULIN⁺ neurons apparently increased because of CTraS induction (Figure 6C). The number of SYNAPSIN1⁺ puncta was significantly higher in neurons derived from CTraS NSs in all the SeV-TiPSC lines (Figure 6D). These data indicate that most of the hiPSC clones efficiently differentiated into neurons following treatment by CdNS even without stringent clone selection.

CTraS Induction Accelerated Disease-Specific Phenotypes in a Model of Neurodegenerative Disease

We examined whether CTraS induction could accelerate *in vitro* aging to efficiently detect phenotypes associated with a late-onset neurodegenerative disease model. We first used iPSC clones derived from a patient with autosomal recessive juvenile Parkinson's disease (PD) due to the loss of PARK2 activity. Since midbrain dopaminergic neurons (mDANs) are selectively damaged in PD, we first modified the CdNS protocol to provide the regional identity around the midbrain (CdNS-MD) (Figure 7A) using Sonic Hedgehog (SHH) and FGF-8 (Gale and Li, 2008). qPCR and immunocytochemical analysis showed increased expression of mDAN markers/populations in both NSs and neurons induced by CdNS-MD compared with the original unbiased CdNS protocol (Figures S6A–S6D). Although both CdNS-MD- and CdNS-derived NSs differentiated into βIII-TUBULIN⁺ neurons at a similar frequency by day 30, the number of TH⁺ dopaminergic neurons was increased among the CdNS-MD-derived cells (Figures 7B and 7C). Before the pathological analysis, we confirmed that the iPSC lines used for the analysis showed similar differentiation efficiency in NS formation and mDAN induction by CdNS-MD (Figures S6E and S6F).

To detect PARK2-specific mitochondrial phenotypes, we treated mDANs derived via CTraS with carbonyl cyanide m-chlorophenylhydrazone (CCCP), which triggers mitophagy by disrupting the mitochondrial membrane potential (Fujimori et al., 2016; Imaizumi et al., 2012; Matsumoto et al., 2016). Although PARK2 neurons at day 30 did not show an accumulation of impaired mitochondria in neurons without CTraS (Figures 7D and 7E), the mDANs induced via CTraS clearly demonstrated PARK2-specific phenotype by day 30 (Figures 7D and 7E) accompanied by a significant decrease of the number of mDANs (Figure 7F). The observed increase in reactive oxygen species production (Fujimori et al., 2016; Matsumoto et al., 2016) only manifested in PARK2 mDANs induced via CTraS (Figures 7G and 7H). Immunocytochemical analysis demonstrated pα-synuclein aggregation (Athauda and Foltynie, 2015) in the neuronal cytoplasm of PARK2-PA cells at day 40 that were derived from the CdNS-MD method (Figures 7I and 7J) with the breakdown of established neurites (Figure 7K). Cell viability analysis using MTT showed a significant decrease of viable PARK2 neurons derived from CTraS after 40 days (Figure 7L). We also observed a significant increase in condensed nuclei that expressed cleaved caspase-3 in iPSC neurons from the PD patient compared

Figure 3. hPSCs Were Rapidly Differentiated toward the Neural Cell Lineage via CTraS Induction
(A) Overview of the culture protocol in this experiment.
(B–D) Sphere formation analysis of NSs derived from untreated PSCs and CTraS PSCs; SOX1 expression (B), relationship between the number and size (C), and total number (D) of the NSs were analyzed on the indicated day (n = 3 independent experiments; mean ± SEM; $^{**}p < 0.01$; Dunnett's test). Scale bar, 400 μm.
(E) Representative images of NSs at day 10 derived from CTraS PSCs with antibodies targeting the indicated markers. Scale bar, 200 μm.
(F) qPCR analysis of the indicated markers in untreated-PSC- and CTraS-PSC-derived NSs (n = 3 independent experiments; mean ± SEM; $^{**}p < 0.01$; Dunnett's test).
(G) Fold change in the endoderm and mesoderm gene expression levels of untreated-PSC- and CTraS-PSC-derived NSs (n = 3 independent experiments; mean ± SEM; $^{**}p < 0.01$; Student's t test).
(H) Immunocytochemical analysis of NSs for A-P and D-V markers. The frequency of NSs containing immunopositive cells is shown as the percentage of total neurospheres (n = 3 independent experiments, mean ± SEM). Scale bar, 100 μm. hPSC lines used: 201B7, WD39, and KhES1.
(I) Comparison of global gene expression profiles of untreated PSCs, CTraS PSCs, untreated NSs, and CTraS NSs. Principal component analysis of the gene expression data. Brown, untreated PSCs; red, CTraS PSCs; blue, untreated NSs; orange, CTraS NSs.
(J) GO analysis of transcripts that were up- and downregulated in CTraS NSs compared with the transcript levels in untreated NSs (a fold change difference of ±2.0).
(K) Top 20 GO terms associated with the upregulated genes in CTraS NSs compared with the levels in untreated NSs (a fold change difference of ±2.0). Red, developmental process and differentiation; green, neural lineage differentiation. hPSC lines used: KA11 and eKA3.
See also Figure S4.

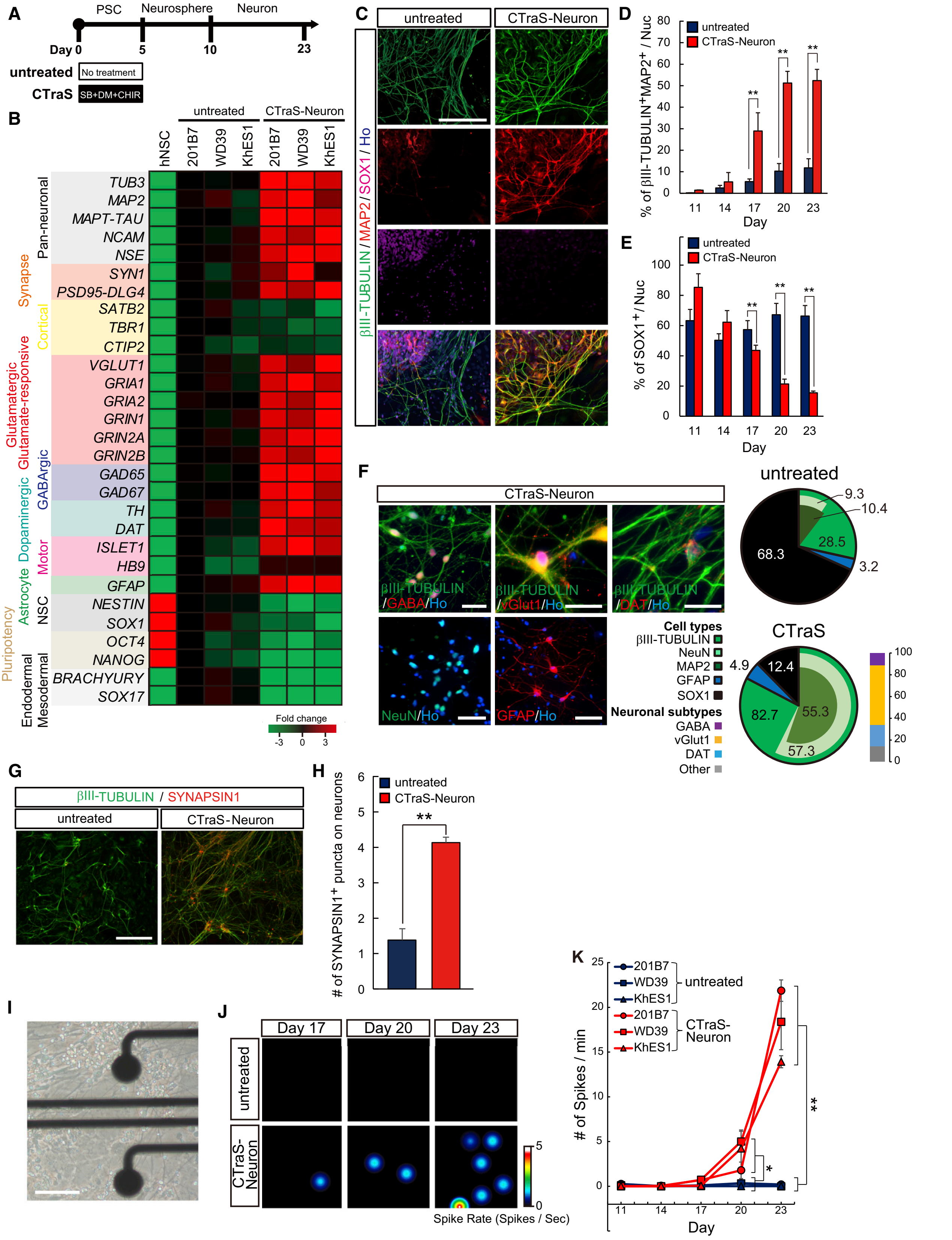

(legend on next page)

with those from a healthy donor, especially among TH^+ dopaminergic neurons (Figure 7M), indicating that PARK2 dopaminergic neurons are more prone to activating cell death programming upon CTraS. Such promotion of *in vitro* pathology via CTraS was also confirmed in familial amyotrophic lateral sclerosis (ALS) models carrying *TARDBP* mutations; the neurite swellings and the reduction of cell viability were reproduced with a shorter culture period by CTraS induction (Figure S7). These data clearly indicate that CTraS induction efficiently accelerates *in vitro* aging to detect phenotypes from early to terminal stage in neurological disease models.

DISCUSSION

In the present study, we have shown that treatment with three small molecules, SB, DM, and CHIR, effectively enhanced the differentiation of hPSCs and changed their state toward a chemically transitional EB-like state, which we have designated CTraS. Interestingly, hPSC-derived cells with CTraS induction differentiated into their respective progenies significantly faster than those without CTraS induction. Using this approach, we demonstrate two advantages of CTraS induction in hiPSC generation and disease modeling. First, stringent colony selection of newly generated PSC clones is not required to eliminate clones that are resistant to differentiation using conventional protocols. Second, CTraS induction accelerated *in vitro* neural maturation and progressive cellular phenotypes in models of neurodegenerative diseases, including PD and ALS. In a previous study, more than 100 days of monolayer differentiation were required to detect an apparent phenotype among neurons derived from disease-specific hiPSCs (Imaizumi et al., 2012; Ohta et al., 2015). By contrast, our method recapitulates several aspects of PD from early to terminal stage after only 30–40 days of culture (Figures 7D–7M). These results suggest that CTraS induction recapitulates aging phenotypes within a shorter period without requiring exogenous factors such as progerin expression and RanBP17 knockdown (Mertens et al., 2015; Miller et al., 2013).

The small molecules we used for CTraS induction have been used in various differentiation protocols (Li et al., 2013). Some researchers have reported the efficacy of the combination of SB, DM, CHIR and/or the other chemicals with the same target pathways to achieve lineage-specific differentiation from PSCs (Chambers et al., 2012; Kriks et al., 2011; Li et al., 2011; Lian et al., 2012). However, these reports referred to their effects on particular lineages, especially in the efficiency of lineage commitment, and there have been few observations regarding the characteristics of the differentiated cells and the other lineages. The outcomes of the SB + DM + CHIR treatment observed in our study are significantly different from these reports. Induced differentiation of hPSCs in a 2D culture using SB + DM + CHIR gives rise to cells at the transitional differentiation state that are committed to all three germ layers, which minimizes the differentiation bias. Based on a global gene expression analysis, the top ten pathways upregulated by CTraS included “cell cycle,” “apoptosis modulation and signaling,” and “senescence and autophagy,” indicating that CTraS augments cellular aging in addition to promoting unbiased differentiation. At present, we cannot exclude the possibility that the enrichment of these pathways indicates the increase of apoptosis due to the addition of

Figure 4. Efficient Generation of Functional Neurons Using Direct Neurosphere Conversion via CTraS
(A) Overview of the culture protocol in this experiment.
(B) Heatmap results derived from the qPCR analysis depicting the relative gene expression levels of the indicated markers. hNSC, hiPSC (201B7)-derived NSCs as described previously (Nori et al., 2011).
(C) Representative images of terminally differentiated derivatives of NSs via CTraS PSCs or not with antibodies targeting the indicated markers. Scale bar, 100 μm.
(D) Neural differentiation analysis quantifying the percentage of βIII-TUBULIN$^+$MAP2$^+$ cells (n = 3 independent experiments; mean ± SEM; $^{**}p < 0.01$; Student’s t test).
(E) Residual neural stem cell analysis quantifying the percentage of SOX1$^+$ cells (n = 3 independent experiments; mean ± SEM; $^{**}p < 0.01$; Student’s t test).
(F) Cell population analysis of terminally differentiated derivatives of untreated PSCs and CTraS PSCs using dNS-based protocols. Scale bar, 50 μm.
(G) SYNAPSIN1 expressions in CTraS and untreated neurons. Scale bar, 100 μm.
(H) Neuronal maturation analysis indicated by the number of SYNAPSIN1$^+$ puncta within βIII-TUBULIN$^+$ neuronal cells at day 23 (n = 3 independent experiments; mean ± SEM; $^{**}p < 0.01$; Student’s t test).
(I) Representative image of cultured neurons on the 64-electrode array on day 23. Scale bar, 100 μm.
(J and K) Electrophysiological analysis of neurons derived from derived from NSs via CTraS-PSC or not using a microelectrode array (MEA) recording system (n = 3 independent experiments; mean ± SEM; $^*p < 0.05$, $^{**}p < 0.01$; Student’s t test).
hPSC lines used: 201B7, WD39, and KhES1. See also Figure S5.

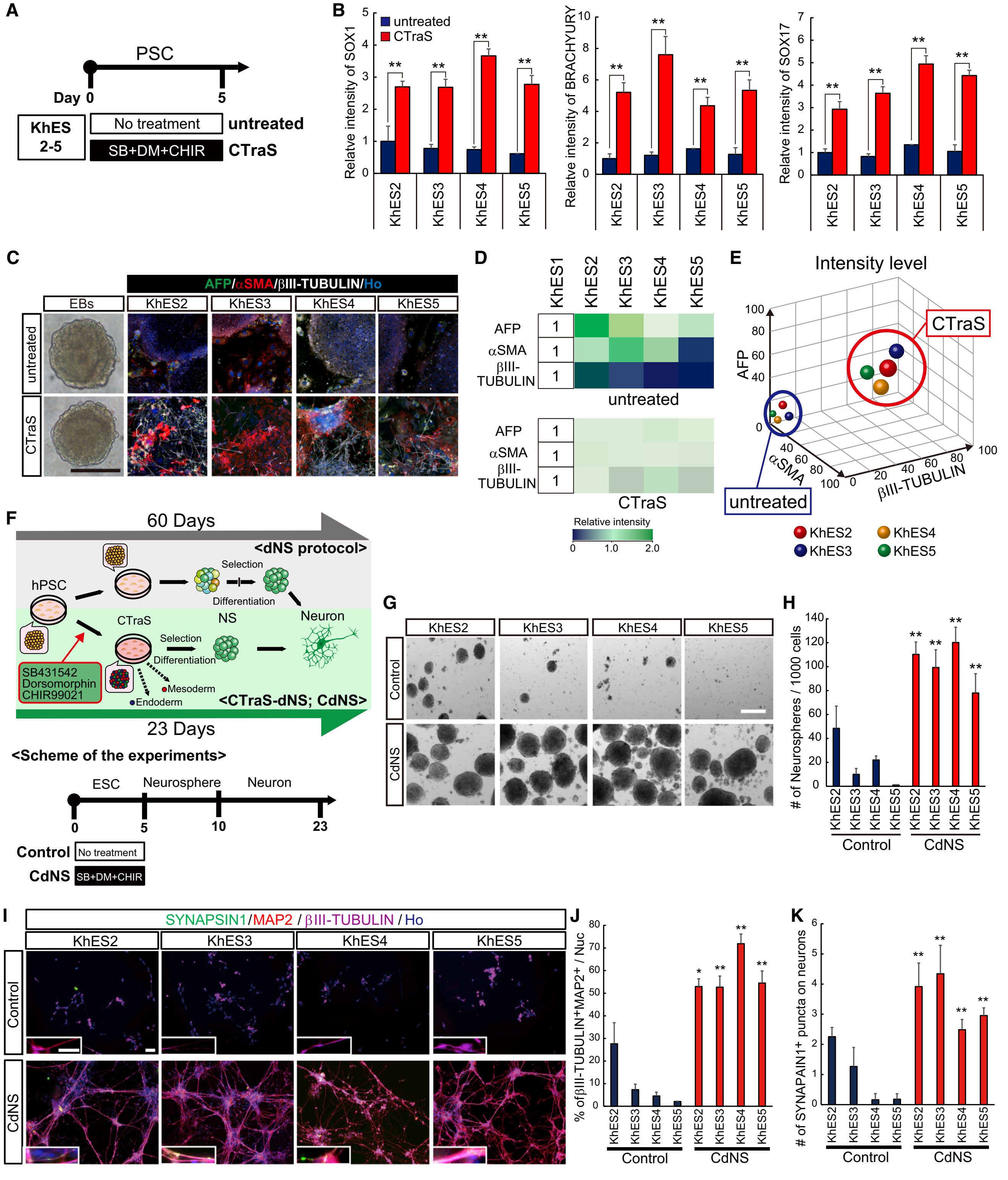

Figure 5. Neuronal Differentiation of Differentiation-Resistant hESC Lines via CTraS

(A) Overview of the culture protocol for CTraS induction using KhESC lines.

(B) Fluorescence intensities of the indicated tridermal lineage markers in untreated PSCs and CTraS PSCs (n = 3 independent experiments; mean ± SEM; $^{**}p < 0.01$; Student's t test).

(legend continued on next page)

exogenous factors. In addition to this concern, progress in the study regarding the mechanism of aging acceleration by CTraS is expected in the future. As one of the molecular biological characteristics of CTraS PSCs, enhanced cholesterol biosynthesis and the mevalonate pathway have been identified. In recent studies, Okamoto-Uchida and colleagues showed that the mevalonate pathway is essential in primitive streak formation using mouse ESCs (Okamoto-Uchida et al., 2016), suggesting the association with the effect of CTraS in terms of differentiation promotion in the PSC stage. From the accumulation of such findings, it is expected that future studies will uncover the whole mechanism of CTraS.

Wnt/β-catenin signaling is known to maintain the balance of self-renewal and differentiation in PSCs in a context-dependent fashion. An imbalance of this activity, such as dysfunction of the transcriptional network underlying pluripotency, promotes differentiation (Abu-Remaileh et al., 2010; Davidson et al., 2012). In addition, several recent studies have reported that Wnt signaling regulates aging in various tissues and stem cells (Chen and Do, 2012; Fujimaki et al., 2015; Naito et al., 2012). In our culture protocol described here, CHIR, which functions as a Wnt activator via GSK-3β inhibition, was used during both the CTraS and NS induction periods. It is possible that activated Wnt signaling influences both terminal differentiation and aging, which may accelerate pathological expression *in vitro*, in the differentiated cells.

Although the detailed molecular mechanisms of CTraS induction via SB + DM + CHIR remain unclear, our results suggest the usefulness of CTraS induction as a general technology for hPSC differentiation. On the basis of our findings, the expansion of CTraS application to the feeder-free culture system without serum replacement in the future is expected to further increase its usefulness. This system may thus contribute to new insights in disease modeling, drug screening, and regenerative medicine using PSCs.

EXPERIMENTAL PROCEDURES

Culture of Undifferentiated ESCs and iPSCs

The human ESC lines KhES1, KhES2, KhES3, KhES4, and KhES5, the control human iPSC lines 201B7, WD39, KA11, KA23, and eKA3, and the PARK2 iPSC lines PB2, PB18, and PB20 were cultured on mitomycin C-treated SNL murine fibroblast feeder cells in standard hESC medium (DMEM/F12, Sigma-Aldrich) containing 20% KnockOut serum replacement (KSR) (Life Technologies), 0.1 mM non-essential amino acids (Sigma-Aldrich), 0.1 mM 2-mercaptoethanol (Sigma-Aldrich), and 4 ng/mL FGF-2 (PeproTech) in an atmosphere containing 3% CO_2. hESCs were used in accordance with the guidelines regarding the utilization of hESCs with approval from the Ministry of Education, Culture, Sports, Science, and Technology (MEXT) of Japan and the Keio University School of Medicine Ethics Committee. All experimental procedures involving iPSCs derived from patients were approved by the Keio University School of Medicine Ethics Committee (approval no. 20080016).

Isolation of Human T Cells and Generation of TiPSCs

Peripheral blood mononuclear cells (PBMCs) were obtained from a healthy donor (race, Japanese; sex, male; age, 26 years) by centrifuging heparinized blood over a Ficoll-Paque PREMIUM gradient (GE Healthcare) according to the manufacturer's instructions. CD3-positive cells were selected using a fluorescently conjugated anti-CD3 mAb (BD Pharmingen). PBMCs and cells subjected to fluorescence-activated cell sorting were seeded on a plate coated with an anti-CD3 mAb and cultured at 37°C in 5% CO_2 in GT-T502 medium (KOHJIN BIO) containing 175 JRU/mL rIL-2. After 5 days of culture, activated PBMCs and activated T cells were transferred to a 96-well plate coated with an anti-CD3 mAb at a density of 1.5×10^3 cells/well and incubated for an additional 24 hr. Thereafter, a solution containing SeV vectors (CytoTune-iPS; ID pharma) was added to the wells. At 24 hr post infection, the medium was replaced with fresh GT-T502 medium. At 48 hr post infection, the cells were collected and transferred to a 96-well plate containing mitomycin C-inactivated SNL feeder cells. After an additional 24 hr, the medium was replaced with hiPSC medium, which was changed every other day until colonies were selected. The generated hiPSCs were maintained on mitomycin

(C) Representative image of EBs and immunocytochemistry based on the *in vitro* three germ-layer assay using KhESC lines. Scale bar, 200 μm.
(D) Heatmap results derived from the fluorescence intensity analysis depicting the relative protein expression levels of the indicated markers. The fluorescence intensity levels were normalized to the mean level of each marker in KhES1 cells.
(E) Fluorescence intensities of the indicated tridermal lineage markers in differentiated EBs induced from untreated PSCs and CTraS PSCs.
(F) Schematic representation of dNS-based neuronal differentiation protocols via CTraS (CTraS-dNS; CdNS) and experimental scheme.
(G) SYNAPSIN1 and MAP2 expressions in CTraS and untreated human ES-derived neurons. Scale bar, 200 μm.
(H) Sphere formation analysis of NSs derived from KhESC lines as reflected by the quantification of the number and size of the NSs using the indicated methods (n = 3 independent experiments; mean ± SEM; **p < 0.01; Student's t test).
(I) Immunostaining of KhESC-derived neurons with antibodies targeting the indicated markers. Scale bar, 100 μm.
(J and K) Neuronal differentiation and maturation analysis as quantified by the percentage of βIII-TUBULIN$^+$MAP2$^+$ cells (J) and the number of SYNAPSIN1$^+$ puncta in βIII-TUBULIN$^+$ neuronal cells (K) (*n* = 3 independent experiments; mean ± SEM; *p < 0.05, **p < 0.01; Student's t test).
hPSC cells used: KhES1, KhES2, KhES3, KhES4, and KhES5.

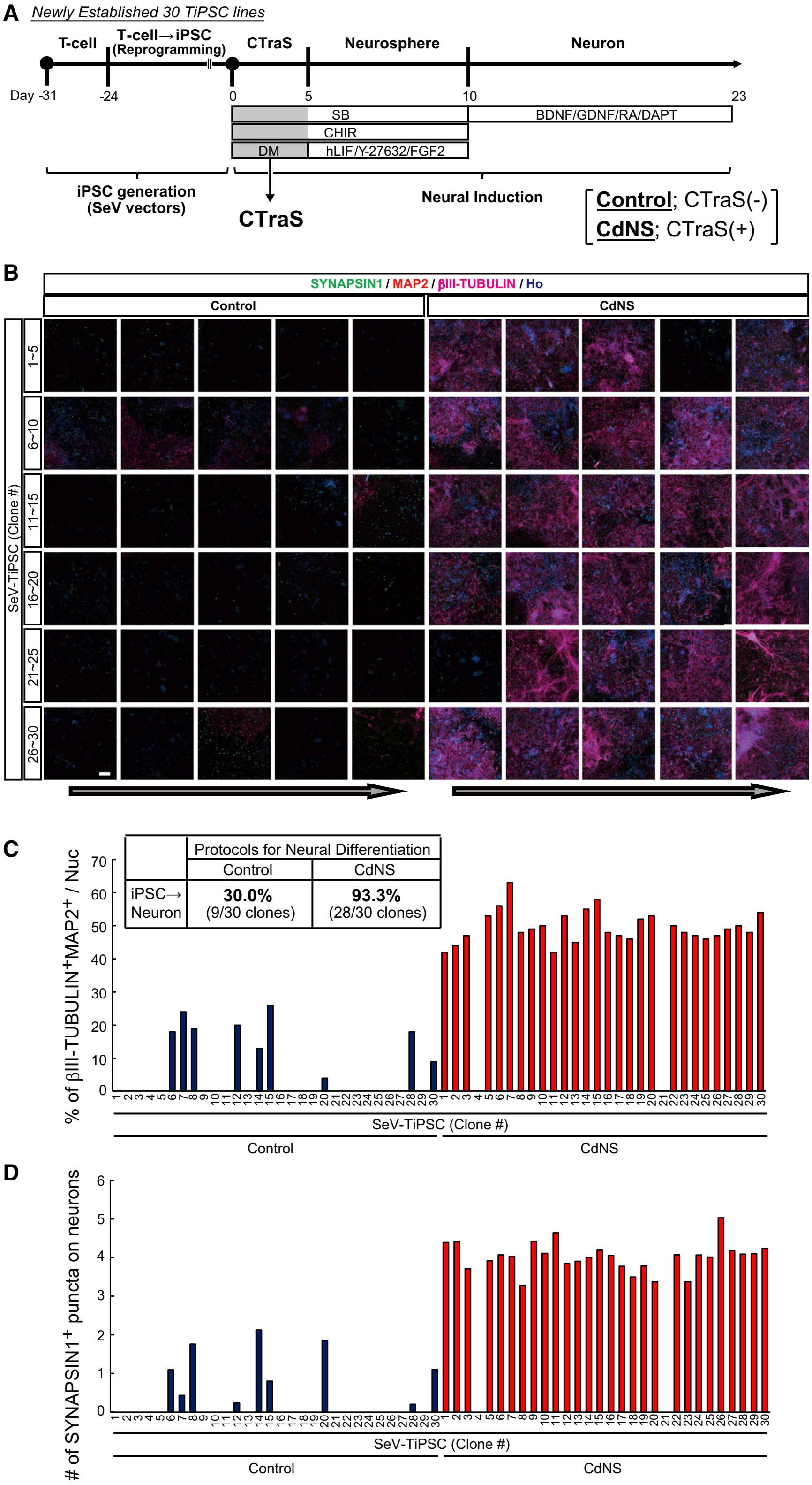

Figure 6. Efficient and Rapid Neuronal Differentiation of Newly Established TiPSCs without Colony Selection

(A) An overview of the culture protocol in this experiment.

(B) Immunostaining with antibodies targeting the indicated markers of neurons derived from 30 newly established TiPSC. Scale bar, 100 μm.

(C) Neuronal differentiation analysis, quantification of the number of neuronal differentiated SeV-TiPSC lines, and their βIII-TUBULIN$^+$MAP2$^+$ cell ratios at day 23 (n = 1).

(D) Neuronal maturation analysis of SYNAPSIN1$^+$ puncta in βIII-TUBULIN$^+$ neuronal cells at day 23 (n = 1).

hPSC lines used: SeV-TiPSC (#1–#30, total 30 lines).

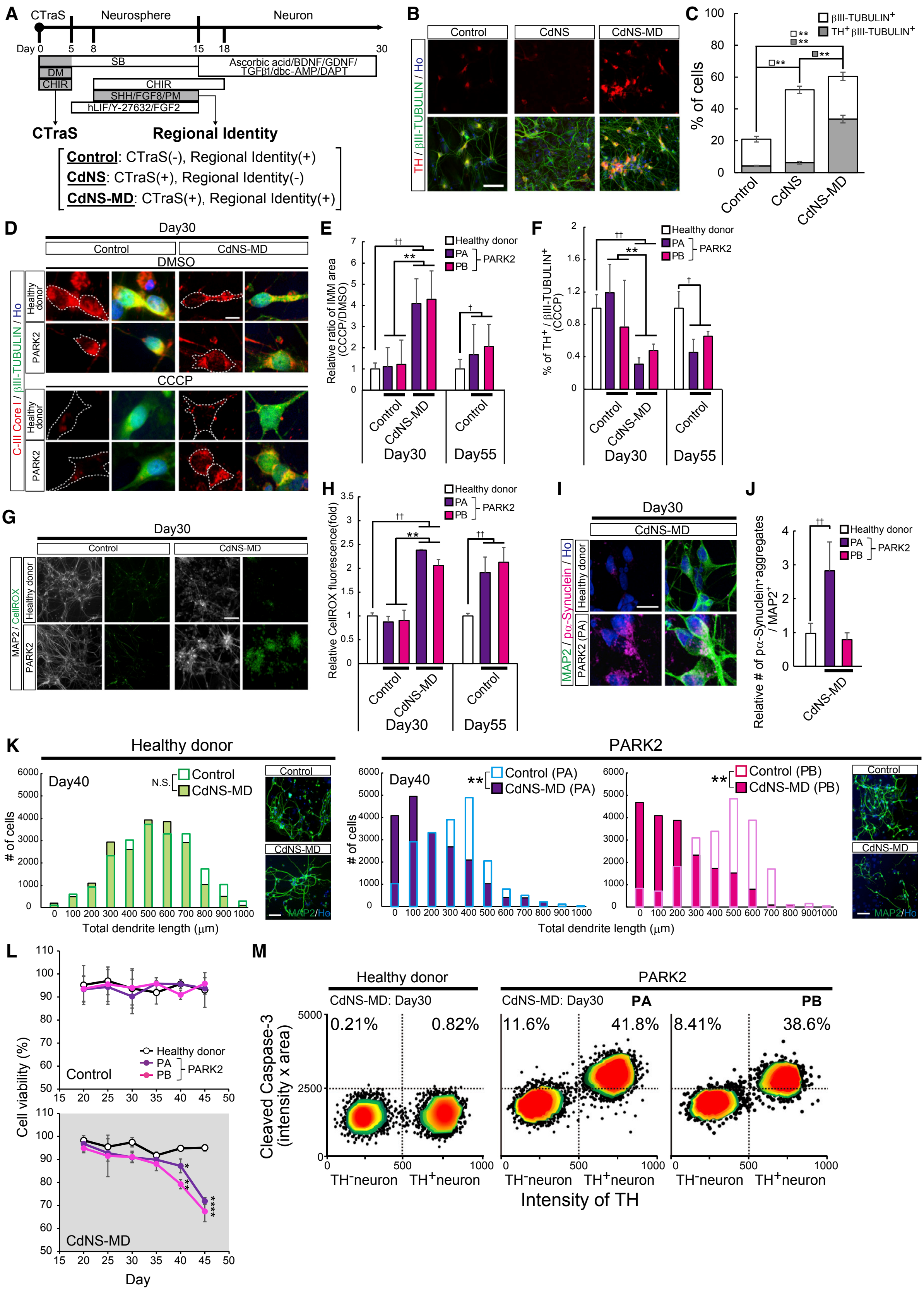

(legend on next page)

C-inactivated SNL feeder cells in hiPSC medium. Healthy donor TiPSC lines (total 30 lines; SeV-TiPSC 1–30) were cultured, and cells at low passage numbers (between 2 and 5) were used for analysis.

ACCESSION NUMBERS

The data reported in this publication have been deposited in the NCBI Gene Expression Omnibus database and are accessible under accession number GEO: GSE95271.

SUPPLEMENTAL INFORMATION

Supplemental Information includes Supplemental Experimental Procedures, seven figures, and three tables and can be found with this article online at https://doi.org/10.1016/j.stemcr.2017.09.024.

AUTHOR CONTRIBUTIONS

K.F., T.M., N.H., H.O., and W.A. conceived and designed the experiments. K.F., T.M., F.K., and W.A. performed the experiments and analyzed data. K.F., H.O., and W.A. wrote and edited the manuscript. N.H. contributed reagents, materials, and analysis tools. All authors read and approved the final manuscript.

ACKNOWLEDGMENTS

We are grateful to Prof. Douglass Sipp (Keio University) for providing invaluable comments on the manuscript and to all the members of the H.O. laboratory for their encouragement and kind support. We also would like to thank N. Nakatsuji and H. Suemori (Kyoto University) for providing hESC clones and S. Yamanaka and M. Nakagawa (Kyoto University) for donating hiPSC clones (201B7). This work was supported by funding from the Research Project for Practical Applications of Regenerative Medicine from Japan Agency for Medical Research and Development (AMED) (grant nos. 15bk0104027h0003, 16bk0104016h0004 to H.O.), the Research Center Network for Realization Research Centers/Projects of Regenerative Medicine (the Program for Intractable Disease Research utilizing disease-specific iPSCs) from the Japan Science and Technology Agency (JST) and AMED (grant nos. 12033011, 15bm0609003h0004, 16bm0609003h0005 to W.A. and H.O.), the New Energy and Industrial Technology Development Organization (NEDO) (grant no. 17be0204436h0004 to W.A. and H.O.), the Japan Society for the Promotion of Science (JSPS) (grant no. JP16K09677 to W.A.), Research Fellowships of Japan Society for the Promotion of Science for Young Scientists (grant no. JP16J06437 to K.F.), the Keio University Grant-in-Aid for the Encouragement of Young Medical Scientists (to K.F.), the Keio University Doctorate Student Grant-in-Aid Program (to K.F.) and a Grant-in-Aid for the Global COE Program from MEXT to Keio University. H.O. is a paid Scientific Advisory Board Member at SanBio Co., Ltd. and K Pharma Inc.

Received: September 16, 2017
Revised: September 26, 2017
Accepted: September 26, 2017
Published: October 26, 2017

Figure 7. CTraS Induction Accelerates Age-Associated Changes and Disease-Specific Phenotypes in a Neurodegenerative Disease Model

(A) Overview of the culture protocol in this experiment.
(B) TH and βIII-TUBULIN expression in neurons from CdNS-MD, CdNS, and control NS. Scale bar, 100 μm.
(C) Neuron and dopaminergic neuron differentiation analysis (n = 3 independent experiments; mean ± SEM; $^{**}p < 0.01$; Tukey's test).
(D) CIII-Core I and βIII-TUBULIN expressions in neurons from CdNS-MD and control NS. Scale bar, 50 μm.
(E) Mitophagy analysis of the CCCP/DMSO ratio of the CIII-Core I in βIII-TUBULIN$^+$ cells (n = 3 independent experiments; mean ± SEM; $^{**}p < 0.01$ control versus CdNS-MD; †p < 0.05, ††p < 0.01 healthy donor versus PARK2; Student's t test).
(F) Stress vulnerability analysis based on the ratio of TH$^+$ neurons after CCCP treatment (n = 3 independent experiments; mean ± SEM; $^{**}p < 0.01$ control versus CdNS-MD; †p < 0.05, ††p < 0.01 healthy donor versus PARK2; Student's t test).
(G) MAP2 immunostaining and CellROX fluorescence in neurons from CdNS-MD and control NS. Scale bars, 500 μm.
(H) Oxidative stress analysis of PARK2 neurons and neurons from a healthy donor (n = 3 independent experiments; mean ± SEM; $^{**}p < 0.01$ control versus CdNS-MD; ††p < 0.01 healthy donor versus PARK2; Student's t test).
(I) MAP2 and pα-syn expressions in neurons from CdNS-MD and control NS. Scale bar, 50 μm.
(J) Quantitative analysis of pα-syn accumulation in neurons differentiated from iPSCs from a healthy donor and PARK2 iPSCs (n = 3 independent experiments; mean ± SEM; ††p < 0.01 healthy donor versus PA; Student's t test).
(K) Dendrite length analysis of iPSC-derived neurons from a PARK2 patient compared with those from healthy donors (n = 3 independent experiments; mean ± SEM; $^{**}P < 0.01$; Kolmogorov-Smirnov test). Scale bar = 50 μm.
(L) Cell viability analysis of iPSC-derived neurons from a PARK2 patient compared with those from healthy donors (n = 3 independent experiments; mean ± SEM; $^{*}p < 0.05$, $^{**}p < 0.01$ healthy donor versus PARK2; Student's t test).
(M) Apoptotic cell population analysis of iPSC-derived neurons from a healthy donor and iPSC-derived neurons from PARK2 patients as gated on their TH intensities.
(D–H, L: n = 3 independent experiments; mean ± SEM; $^{**}p < 0.01$ Control vs CdNS-MD; †p < 0.05, ††p < 0.01 healthy donor vs PARK2; Student's t-test).
hPSC lines used: KA11, KA23, and eKA3 (healthy donor); PA1, PA9, and PA22 (PARK2-PA); PB2, PB18, and PB20 (PARK2-PB). See also Figures S6 and S7.

REFERENCES

Abu-Remaileh, M., Gerson, A., Farago, M., Nathan, G., Alkalay, I., Zins Rousso, S., Gur, M., Fainsod, A., and Bergman, Y. (2010). Oct-3/4 regulates stem cell identity and cell fate decisions by modulating Wnt/beta-catenin signalling. EMBO J. *29*, 3236–3248.

Athauda, D., and Foltynie, T. (2015). The ongoing pursuit of neuroprotective therapies in Parkinson disease. Nat. Rev. Neurol. *11*, 25–40.

Chambers, S.M., Qi, Y., Mica, Y., Lee, G., Zhang, X.J., Niu, L., Bilsland, J., Cao, L., Stevens, E., Whiting, P., et al. (2012). Combined small-molecule inhibition accelerates developmental timing and converts human pluripotent stem cells into nociceptors. Nat. Biotechnol. *30*, 715–720.

Chen, M., and Do, H. (2012). Wnt signaling in neurogenesis during aging and physical activity. Brain Sci. *2*, 745–768.

Clevers, H., Loh, K.M., and Nusse, R. (2014). Stem cell signaling. An integral program for tissue renewal and regeneration: Wnt signaling and stem cell control. Science *346*, 1248012.

Davidson, K.C., Adams, A.M., Goodson, J.M., McDonald, C.E., Potter, J.C., Berndt, J.D., Biechele, T.L., Taylor, R.J., and Moon, R.T. (2012). Wnt/beta-catenin signaling promotes differentiation, not self-renewal, of human embryonic stem cells and is repressed by Oct4. Proc. Natl. Acad. Sci. USA *109*, 4485–4490.

Di-Gregorio, A., Sancho, M., Stuckey, D.W., Crompton, L.A., Godwin, J., Mishina, Y., and Rodriguez, T.A. (2007). BMP signalling inhibits premature neural differentiation in the mouse embryo. Development *134*, 3359–3369.

Fujimaki, S., Wakabayashi, T., Takemasa, T., Asashima, M., and Kuwabara, T. (2015). The regulation of stem cell aging by Wnt signaling. Histol. Histopathol. *30*, 1411–1430.

Fujimori, K., Tezuka, T., Ishiura, H., Mitsui, J., Doi, K., Yoshimura, J., Tada, H., Matsumoto, T., Isoda, M., Hashimoto, R., et al. (2016). Modeling neurological diseases with induced pluripotent cells reprogrammed from immortalized lymphoblastoid cell lines. Mol. Brain *9*, 88.

Gale, E., and Li, M. (2008). Midbrain dopaminergic neuron fate specification: of mice and embryonic stem cells. Mol. Brain *1*, 8.

Hawes, S., and Pera, M.F. (2006). Identification and maintenance of cell lineage progenitors derived from human ES cells. In Essentials of Stem Cell Biology, R. Lanza and A. Atala, eds. (Elsevier), pp. 355–362.

Ichiyanagi, N., Fujimori, K., Yano, M., Ishihara-Fujisaki, C., Sone, T., Akiyama, T., Okada, Y., Akamatsu, W., Matsumoto, T., Ishikawa, M., et al. (2016). Establishment of in vitro FUS-associated familial amyotrophic lateral sclerosis model using human induced pluripotent stem cells. Stem Cell Reports *6*, 496–510.

Imaizumi, Y., Okada, Y., Akamatsu, W., Koike, M., Kuzumaki, N., Hayakawa, H., Nihira, T., Kobayashi, T., Ohyama, M., Sato, S., et al. (2012). Mitochondrial dysfunction associated with increased oxidative stress and alpha-synuclein accumulation in PARK2 iPSC-derived neurons and postmortem brain tissue. Mol. Brain *5*, 35.

Imaizumi, K., Sone, T., Ibata, K., Fujimori, K., Yuzaki, M., Akamatsu, W., and Okano, H. (2015). Controlling the regional identity of hPSC-derived neurons to uncover neuronal subtype specificity of neurological disease phenotypes. Stem Cell Reports *5*, 1010–1022.

Imamura, K., Izumi, Y., Watanabe, A., Tsukita, K., Woltjen, K., Yamamoto, T., Hotta, A., Kondo, T., Kitaoka, S., Ohta, A., et al. (2017). The Src/c-Abl pathway is a potential therapeutic target in amyotrophic lateral sclerosis. Sci. Transl. Med. *9*. https://doi.org/10.1126/scitranslmed.aaf3962.

Kim, D.S., Lee, J.S., Leem, J.W., Huh, Y.J., Kim, J.Y., Kim, H.S., Park, I.H., Daley, G.Q., Hwang, D.Y., and Kim, D.W. (2010). Robust enhancement of neural differentiation from human ES and iPS cells regardless of their innate difference in differentiation propensity. Stem Cell Rev. *6*, 270–281.

Kim, K., Zhao, R., Doi, A., Ng, K., Unternaehrer, J., Cahan, P., Huo, H., Loh, Y.H., Aryee, M.J., Lensch, M.W., et al. (2011). Donor cell type can influence the epigenome and differentiation potential of human induced pluripotent stem cells. Nat. Biotechnol. *29*, 1117–1119.

Kobayashi, Y., Okada, Y., Itakura, G., Iwai, H., Nishimura, S., Yasuda, A., Nori, S., Hikishima, K., Konomi, T., Fujiyoshi, K., et al. (2012). Pre-evaluated safe human iPSC-derived neural stem cells promote functional recovery after spinal cord injury in common marmoset without tumorigenicity. PLoS One *7*, e52787.

Kriks, S., Shim, J.W., Piao, J., Ganat, Y.M., Wakeman, D.R., Xie, Z., Carrillo-Reid, L., Auyeung, G., Antonacci, C., Buch, A., et al. (2011). Dopamine neurons derived from human ES cells efficiently engraft in animal models of Parkinson's disease. Nature *480*, 547–551.

Li, W., Sun, W., Zhang, Y., Wei, W., Ambasudhan, R., Xia, P., Talantova, M., Lin, T., Kim, J., Wang, X., et al. (2011). Rapid induction and long-term self-renewal of primitive neural precursors from human embryonic stem cells by small molecule inhibitors. Proc. Natl. Acad. Sci. USA *108*, 8299–8304.

Li, W., Li, K., Wei, W., and Ding, S. (2013). Chemical approaches to stem cell biology and therapeutics. Cell Stem Cell *13*, 270–283.

Lian, X., Hsiao, C., Wilson, G., Zhu, K., Hazeltine, L.B., Azarin, S.M., Raval, K.K., Zhang, J., Kamp, T.J., and Palecek, S.P. (2012). Robust cardiomyocyte differentiation from human pluripotent stem cells via temporal modulation of canonical Wnt signaling. Proc. Natl. Acad. Sci. USA *109*, E1848–E1857.

Matsumoto, T., Fujimori, K., Andoh-Noda, T., Ando, T., Kuzumaki, N., Toyoshima, M., Tada, H., Imaizumi, K., Ishikawa, M., Yamaguchi, R., et al. (2016). Functional neurons generated from T cell-derived induced pluripotent stem cells for neurological disease modeling. Stem Cell Reports *6*, 422–435.

Mertens, J., Paquola, A.C., Ku, M., Hatch, E., Bohnke, L., Ladjevardi, S., McGrath, S., Campbell, B., Lee, H., Herdy, J.R., et al. (2015). Directly reprogrammed human neurons retain aging-associated transcriptomic signatures and reveal age-related nucleocytoplasmic defects. Cell Stem Cell *17*, 705–718.

Miller, J.D., Ganat, Y.M., Kishinevsky, S., Bowman, R.L., Liu, B., Tu, E.Y., Mandal, P.K., Vera, E., Shim, J.W., Kriks, S., et al. (2013). Human iPSC-based modeling of late-onset disease via progerin-induced aging. Cell Stem Cell *13*, 691–705.

Naito, A.T., Sumida, T., Nomura, S., Liu, M.L., Higo, T., Nakagawa, A., Okada, K., Sakai, T., Hashimoto, A., Hara, Y., et al. (2012). Complement C1q activates canonical Wnt signaling and promotes aging-related phenotypes. Cell *149*, 1298–1313.

Ng, E.S., Davis, R.P., Azzola, L., Stanley, E.G., and Elefanty, A.G. (2005). Forced aggregation of defined numbers of human embryonic stem cells into embryoid bodies fosters robust, reproducible hematopoietic differentiation. Blood *106*, 1601–1603.

Nori, S., Okada, Y., Yasuda, A., Tsuji, O., Takahashi, Y., Kobayashi, Y., Fujiyoshi, K., Koike, M., Uchiyama, Y., Ikeda, E., et al. (2011). Grafted human-induced pluripotent stem-cell-derived neurospheres promote motor functional recovery after spinal cord injury in mice. Proc. Natl. Acad. Sci. USA *108*, 16825–16830.

Ogawa, S., Surapisitchat, J., Virtanen, C., Ogawa, M., Niapour, M., Sugamori, K.S., Wang, S., Tamblyn, L., Guillemette, C., Hoffmann, E., et al. (2013). Three-dimensional culture and cAMP signaling promote the maturation of human pluripotent stem cell-derived hepatocytes. Development *140*, 3285–3296.

Ohta, E., Nihira, T., Uchino, A., Imaizumi, Y., Okada, Y., Akamatsu, W., Takahashi, K., Hayakawa, H., Nagai, M., Ohyama, M., et al. (2015). I2020T mutant LRRK2 iPSC-derived neurons in the Sagamihara family exhibit increased Tau phosphorylation through the AKT/GSK-3beta signaling pathway. Hum. Mol. Genet. *24*, 4879–4900.

Okamoto-Uchida, Y., Yu, R., Miyamura, N., Arima, N., Ishigami-Yuasa, M., Kagechika, H., Yoshida, S., Hosoya, T., Nawa, M., Kasama, T., et al. (2016). The mevalonate pathway regulates primitive streak formation via protein farnesylation. Sci. Rep. *6*, 37697.

Okano, H., Nakamura, M., Yoshida, K., Okada, Y., Tsuji, O., Nori, S., Ikeda, E., Yamanaka, S., and Miura, K. (2013). Steps toward safe cell therapy using induced pluripotent stem cells. Circ. Res. *112*, 523–533.

Osafune, K., Caron, L., Borowiak, M., Martinez, R.J., Fitz-Gerald, C.S., Sato, Y., Cowan, C.A., Chien, K.R., and Melton, D.A. (2008). Marked differences in differentiation propensity among human embryonic stem cell lines. Nat. Biotechnol. *26*, 313–315.

Piccolo, S., Sasai, Y., Lu, B., and De Robertis, E.M. (1996). Dorsoventral patterning in *Xenopus*: inhibition of ventral signals by direct binding of chordin to BMP-4. Cell *86*, 589–598.

Polo, J.M., Liu, S., Figueroa, M.E., Kulalert, W., Eminli, S., Tan, K.Y., Apostolou, E., Stadtfeld, M., Li, Y., Shioda, T., et al. (2010). Cell type of origin influences the molecular and functional properties of mouse induced pluripotent stem cells. Nat. Biotechnol. *28*, 848–855.

Ring, D.B., Johnson, K.W., Henriksen, E.J., Nuss, J.M., Goff, D., Kinnick, T.R., Ma, S.T., Reeder, J.W., Samuels, I., Slabiak, T., et al. (2003). Selective glycogen synthase kinase 3 inhibitors potentiate insulin activation of glucose transport and utilization in vitro and in vivo. Diabetes *52*, 588–595.

Tabar, V., and Studer, L. (2014). Pluripotent stem cells in regenerative medicine: challenges and recent progress. Nat. Rev. Genet. *15*, 82–92.

Valtorta, F., Pozzi, D., Benfenati, F., and Fornasiero, E.F. (2011). The synapsins: multitask modulators of neuronal development. Semin. Cell Dev. Biol. *22*, 378–386.

Wilson, P.A., and Hemmati-Brivanlou, A. (1995). Induction of epidermis and inhibition of neural fate by Bmp-4. Nature *376*, 331–333.

Yang, L., Soonpaa, M.H., Adler, E.D., Roepke, T.K., Kattman, S.J., Kennedy, M., Henckaerts, E., Bonham, K., Abbott, G.W., Linden, R.M., et al. (2008). Human cardiovascular progenitor cells develop from a KDR+ embryonic-stem-cell-derived population. Nature *453*, 524–528.

Stem Cell Reports, Volume *9*

Supplemental Information

Escape from Pluripotency via Inhibition of TGF-β/BMP and Activation of Wnt Signaling Accelerates Differentiation and Aging in hPSC Progeny Cells

Koki Fujimori, Takuya Matsumoto, Fumihiko Kisa, Nobutaka Hattori, Hideyuki Okano, and Wado Akamatsu

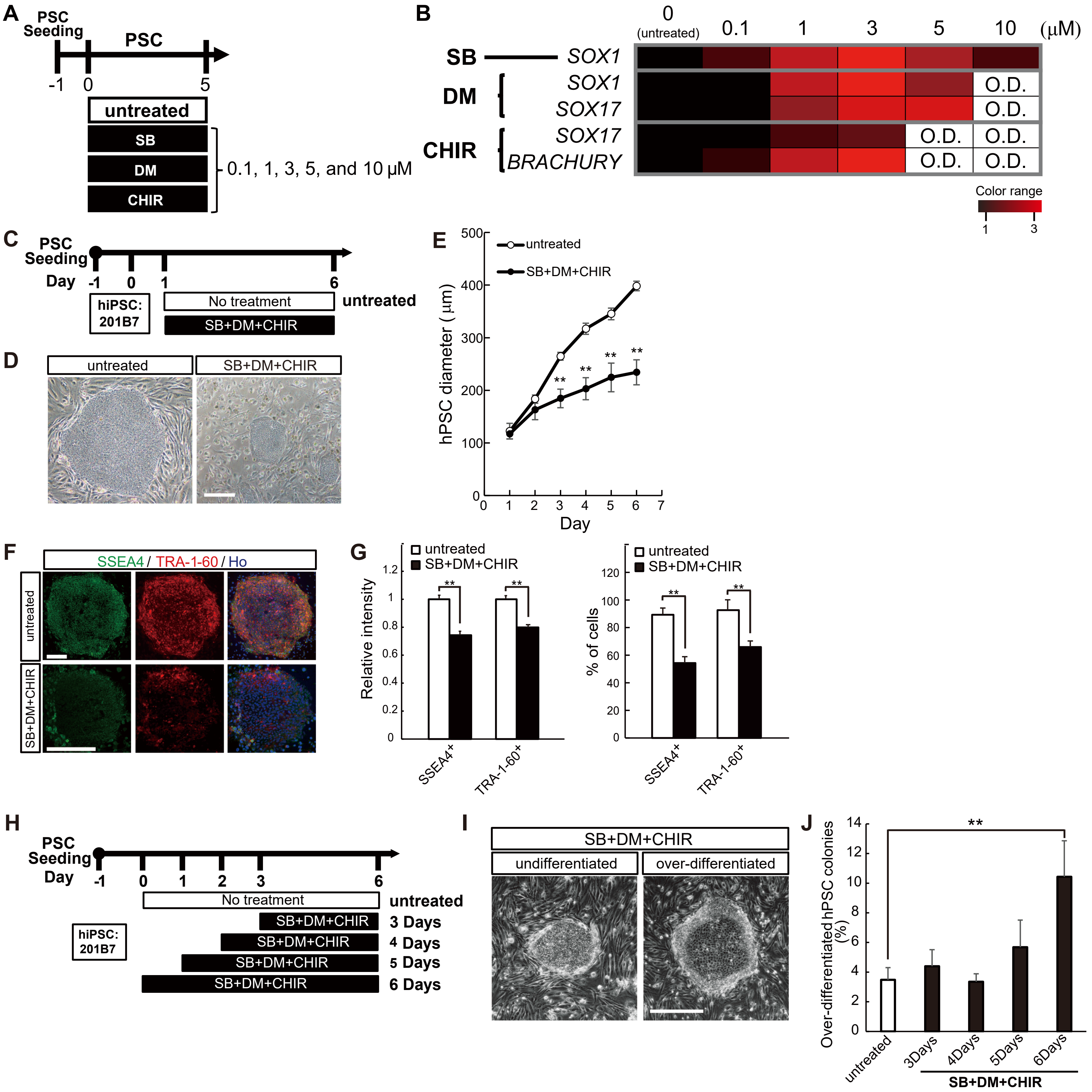

Figure S1 Effects of SB, DM, and/or CHIR Treatment on the Lineage-Specific Differentiation, Morphology, and Pluripotency of hPSC. (related to Figure 1)

(A) Overview of the culture protocol in this experiment. (B) qPCR analysis of three-germ layers markers in PSC treated SB, DM, or CHIR with indicated dose for 5 days (n=3 independent experiments). “O.D.” indicates Over Differentiated PSC colony showing doughnut-like morphology. (C) Schematic of the experiments regarding the time-course of treatment with the three small molecules. (D) Representative images of untreated hPSCs and SB-, DM-, and CHIR-treated hPSCs. Scale bars = 200 μm. (E) Differences in the colony size of untreated hPSCs and SB+DM+CHIR treated-hPSCs (n = 3 independent experiments; mean ± SEM; $**P < 0.01$; Student’s *t*-test). (F) Immunostaining of untreated hPSCs and SB+DM+CHIR-treated hPSCs for the indicated pluripotent markers. (G) Intensity and frequency of the indicated pluripotent markers in untreated hPSCs and SB+DM+CHIR treated-hPSC (n = 3 independent experiments; mean ± SEM; $**P < 0.01$; Student’s *t*-test). (H) Schematic of experiments for the time-course of three small molecules treatment. (I) Representative morphology of undifferentiated hPSCs (untreated hPSCs) and over-differentiated hPSCs (6 Days SB+DM+CHIR-treated hPSCs) on day 6 after hPSC seeding. Scale bars = 200 μm. (J) Percentage of over-differentiated hPSC colonies (n = 3 independent experiments; mean ± SEM; $**P < 0.01$; Dunnett’s test). hPSC lines used: 201B7

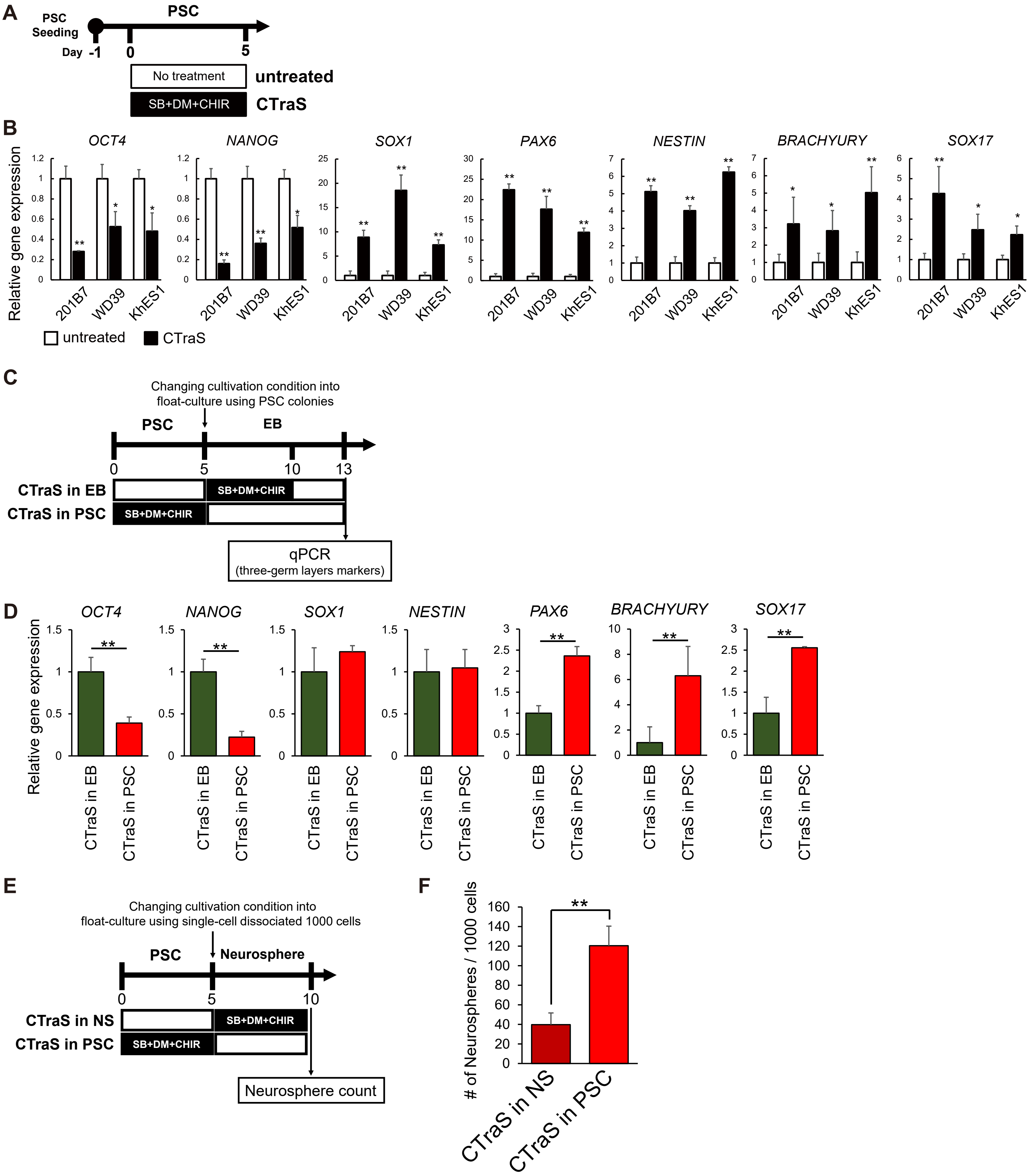

Figure S2 Enhancement of the Three-Germ Layers Differentiation via CTraS in PSC-Stage. (related to Figure 1 and 2)

(A) Overview of the culture protocol used in this experiment. (B) qPCR analysis for the indicated genes in three hPSC lines cultured with SB, DM, and CHIR for 5 days (n = 3 independent experiments; mean ± SEM; $*P < 0.05$, $**P < 0.01$; Student's *t*-test). (C) Overview of the culture protocol in this experiment. (D) qPCR analysis of pluripotent markers and three-germ layers markers in EBs with CTraS induction at two different stages of differentiation (n=3 independent experiments; mean ± SEM; $**P < 0.01$; Student's *t*-test). (E) Overview of the culture protocol in this experiment. (F) Sphere formation analysis of neurospheres (n=3 independent experiments; mean ± SEM; $**P < 0.01$; Student's *t*-test). hPSC lines used: 201B7, WD39, and KhES1

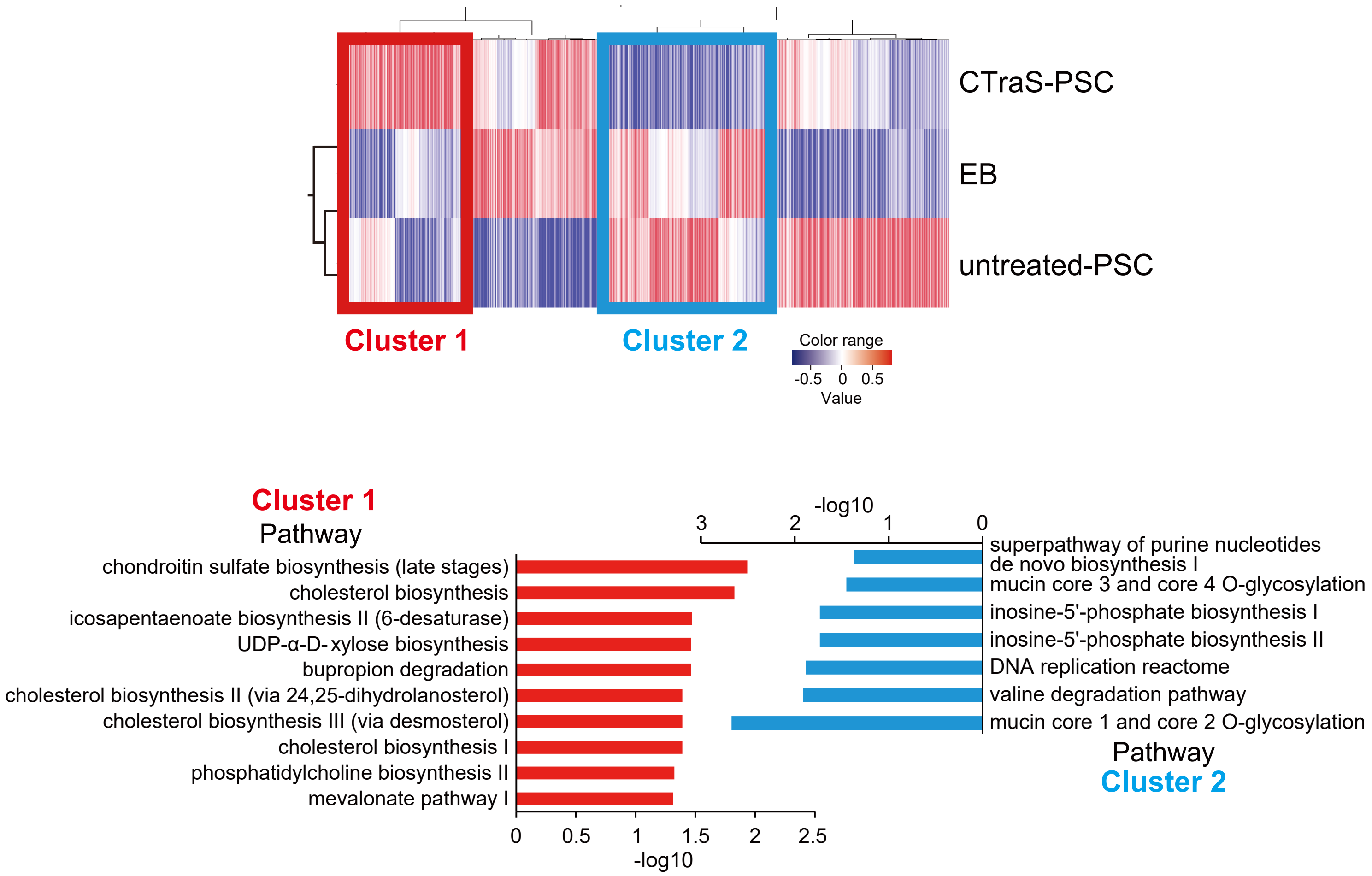

Figure S3 Pathway Analysis of the Gene-Set Showing the CTraS-Specific Expression Pattern. (related to Figure 2)

Pathway analysis of the extracted gene-set with different expression pattern in CTraS-PSC compared with EB and untreated-PSC. The data reported in this publication have been deposited in the NCBI Gene Expression Omnibus (GEO, http://www.ncbi.nlm.nih.gov/geo/) database and are accessible using GEO Series accession number GSE95271. hPSC line used; KA11 and eKA3

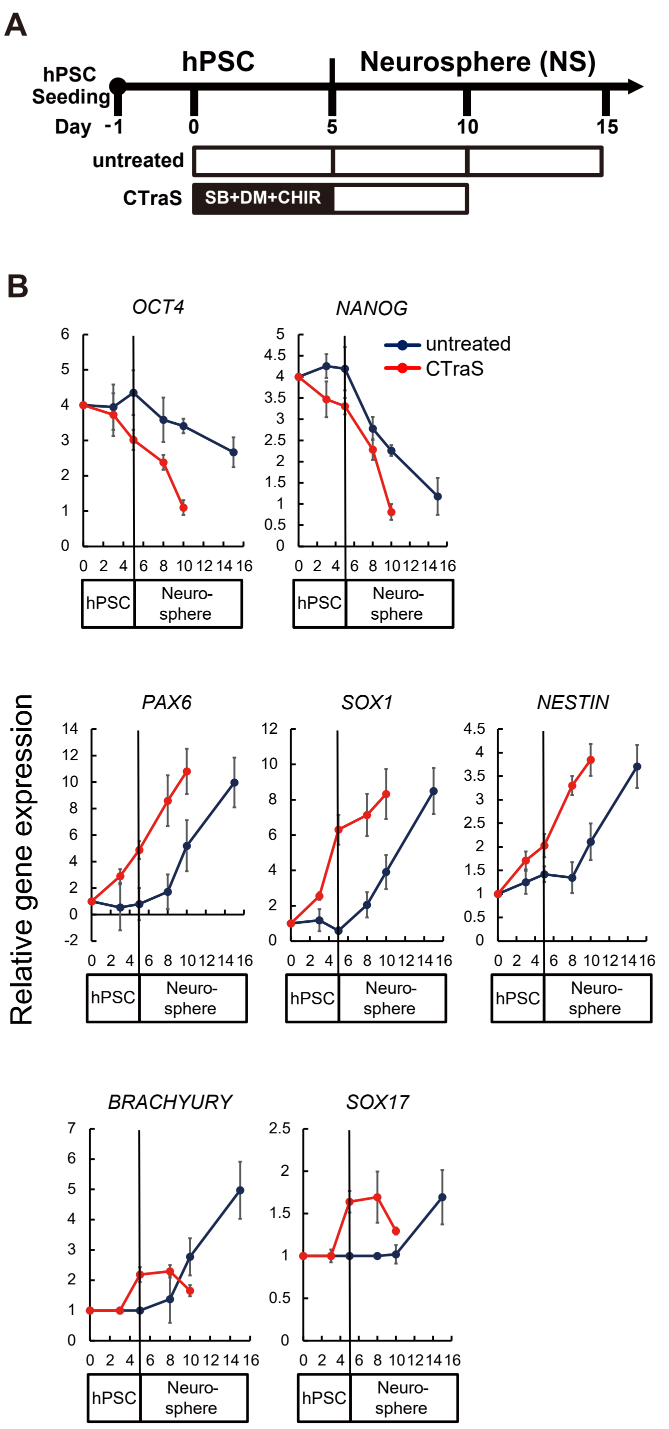

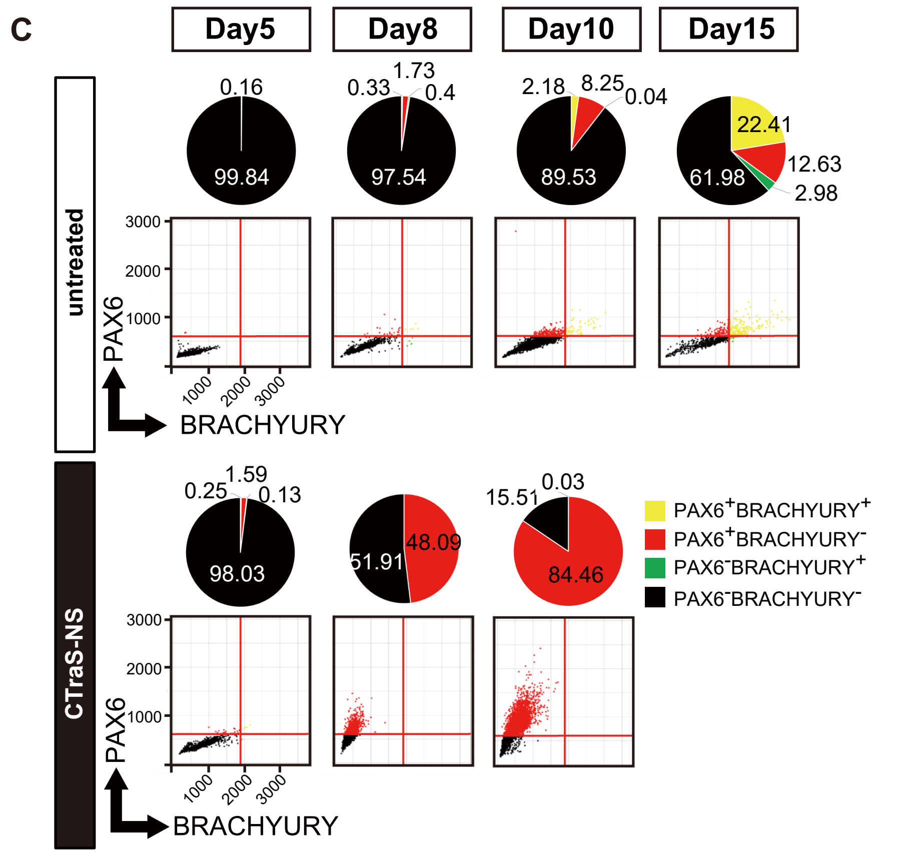

D

CTraS-NS < untreated-NS (812 genes) **Developmental Process** and **Differentiation**

GO ACCESSION	GO Term	p-value
GO:0009790|GO:0009795	embryo development	8.63E-24
GO:0007275	multicellular organism development	5.80E-23
GO:0032502	developmental process	2.08E-22
GO:0048856	anatomical structure development	1.04E-21
GO:0044767	single-organism developmental process	1.07E-21
GO:0044707	single-multicellular organism process	3.20E-20
GO:0048568	embryonic organ development	9.58E-20
GO:0048731	system development	1.30E-19
GO:0048598|GO:0048828	embryonic morphogenesis	1.70E-19
GO:0048706	embryonic skeletal system development	1.78E-19
GO:0009952	anterior/posterior pattern specification	1.90E-19
GO:0072358	cardiovascular system development	1.92E-19
GO:0072359	circulatory system development	1.92E-19
GO:0048513	animal organ development	3.14E-18
GO:0007389	pattern specification process	4.28E-18
GO:0043009	chordate embryonic development	6.61E-18
GO:0009653	anatomical structure morphogenesis	8.92E-18
GO:0001501	skeletal system development	1.01E-17
GO:0009792	embryo development ending in birth or egg hatching	1.31E-17
GO:0032501|GO:0050874	multicellular organismal process	3.03E-17

Figure S4 CTraS-hPSCs Exhibited Accelerated Lineage-Specific Differentiation toward the Ectoderm during NS Formation. (related to Figure 3)
(A) An overview of the culture protocol used in this experiment. (B) Time-dependent changes in gene expression from hPSCs to NSs via CTraS or traditional differentiation methods (n = 3 independent experiments; mean ± SEM). hPSC lines used: 201B7, WD39, and KhES1 (C) Cell population analysis of the NSs induced from untreated hPSCs and CTraS-hPSCs. NSs were single-cell dissociated and stained for the indicated markers. (D) The gene ontology analysis of the transcripts down-regulated in CTraS-NSs vs untreated NSs. hPSC line used; KA11 and eKA3

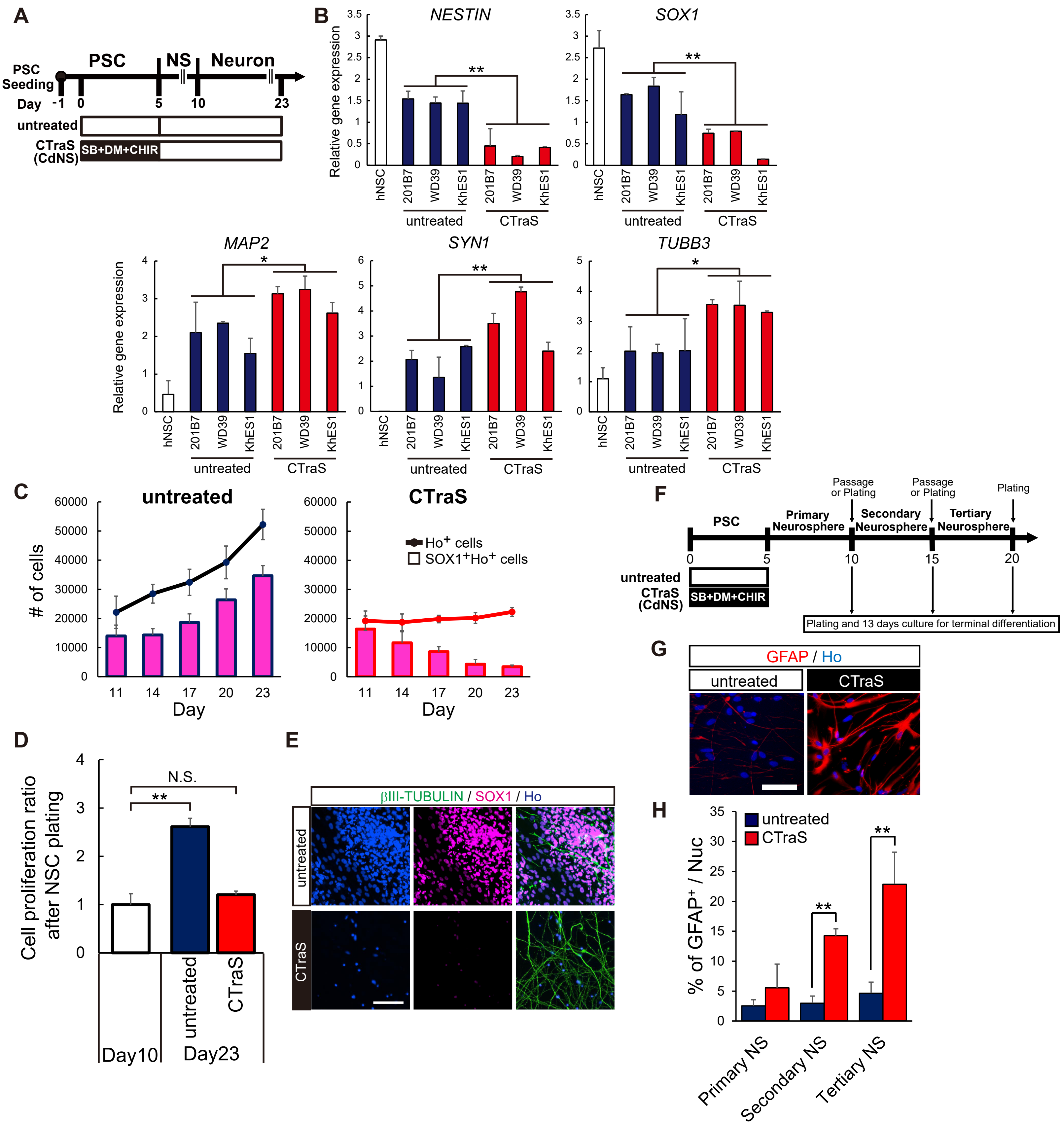

Figure S5 CdNS Accelerated the Generation of Neurons and Astrocytes almost without Residual Stem Cells. (related to Figure 4)

(A) An overview of the culture protocol used in this experiment. (B) qPCR analysis for indicated genes on three hPSC lines-derived neurons via CTraS or not (n = 3 independent experiments; mean ± SEM; **p* < 0.05, ***p* < 0.01; Dunnett's test). (C) Residual NSC analysis, quantifying SOX1^{+} cell ratio (n = 3 independent experiments; mean ± SEM). (D) Cell proliferation analysis after plating single-cell dissociated NSs via CTraS or not (n = 3 independent experiments; mean ± SEM; ***p* < 0.01; Dunnett's test). (E) Immunostaining of untreated-hPSC- and CTraS-hPSC-derived neurons and NSCs with antibodies raised against the indicated markers. Scale bar = 100 μm. (F) An overview of this experiment. (G) Representative images of cultures with or without terminal differentiation of tertiary neurospheres via CTraS. (H) Quatitative analysis of GFAP^{+} cells in terminal differentiations via CTraS or not (n=3 independent experiments; mean ± SEM; ***P* < 0.01; Student's *t*-test). Scale bar =200 μm. hPSC lines used: 201B7, WD39, and KhES1

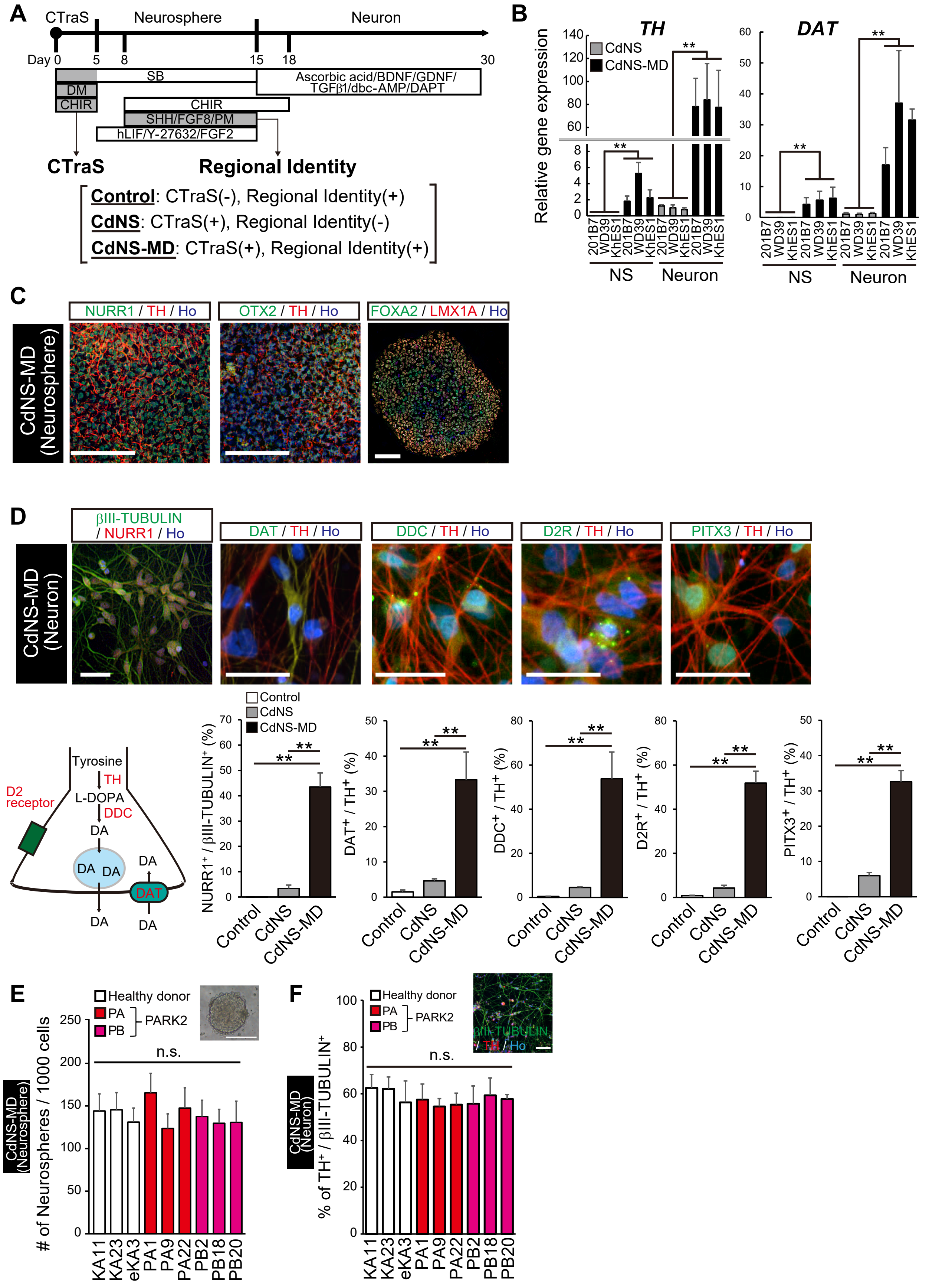

Figure S6 Differentiation of Dopaminergic Neuron via CTraS. (related to Figure 7)
(A) Overview of the culture protocol for region-specific differentiation based on CdNS. (B) qPCR analysis for the indicated expression of dopaminergic neuronal genes in NSs and neurons derived from hPSCs with the indicated protocol (n = 3 independent experiments; mean ± SEM; $^{**}P < 0.01$; Student's *t*-test). (C) Immunostaining and cell population analysis of hPSC-derived NSs at day 15 using CdNS-MD. Scale bars = 100 μm. (D) Immunostaining and cell population analysis of hPSC-derived Neurons at day 30 using indicated protocols (n = 3 independent experiments; mean ± SEM; $^{**}P < 0.01$; Dunnett's test). Scale bars = 30 μm. hPSC lines used: 201B7, WD39, and KhES1. (E) Sphere formation analysis of NSs at day 15 by using CdNS-MD (n = 3 independent experiments; mean ± SEM; n.s., not significant; ANOVA). Scale bar = 200 μm. (F) Immunostaining and cell population analysis of dopaminergic neurons at day30 differentiated from healthy donor-derived iPSCs and PARK2-iPSCs (n = 3 independent experiments; mean ± SEM; n.s., not significant; ANOVA). Scale bar = 70 μm. hPSC lines used: KA11, KA23, and eKA3 (healthy donor); PA1, PA9, and PA22 (PARK2-PA); PB2, PB18, and PB20 (PARK2-PB).

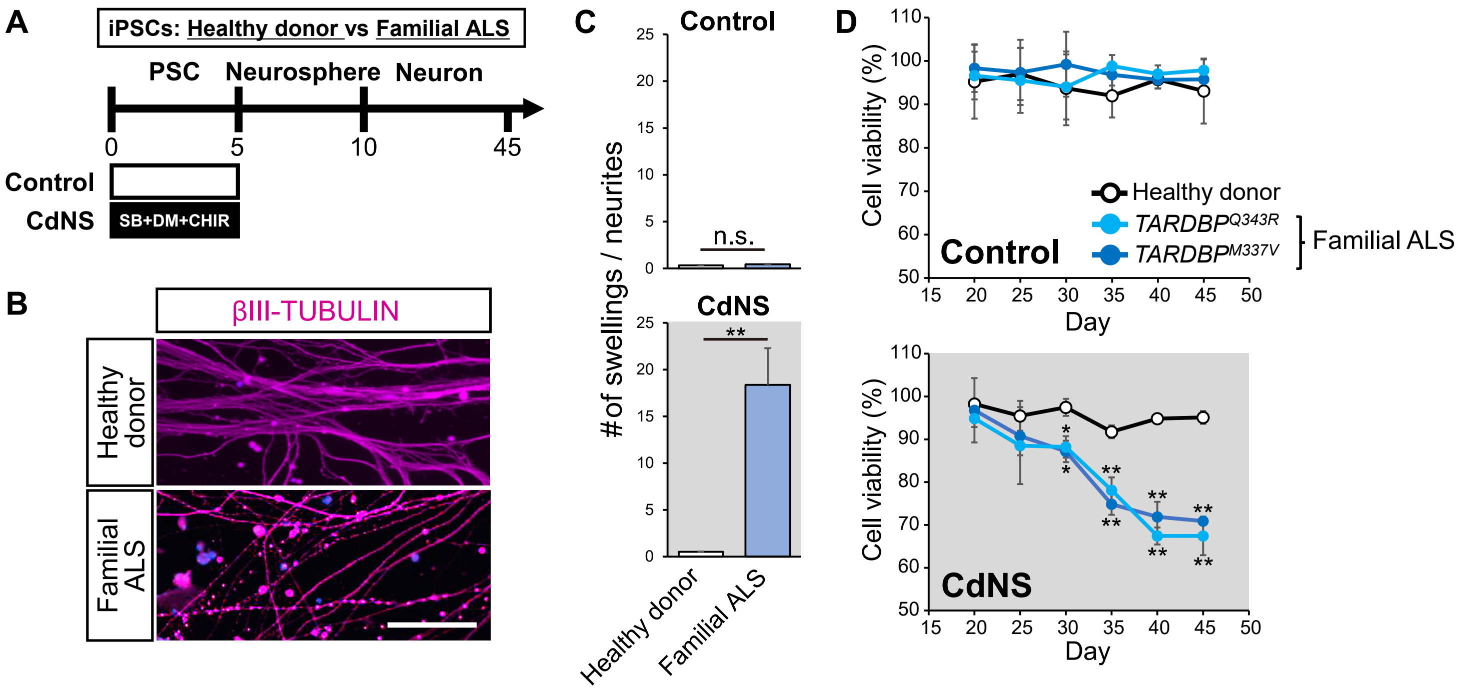

Figure S7 Acceleration of the *In Vitro* ALS Phenotype Expression by CTraS. (related to Figure 7)
(A) Overview of the culture protocol in the experiment using familial ALS patients-iPSCs. (B) Representative images of neurites. Familial ALS-iPSC derived neurons exhibited swollen neurites at day 25. Scale bar = 100 µm. (C) Quantitative data of the number of swellings in neurons at day 25 (n=3 independent experiments; mean ± SEM; $**P < 0.01$; Student's *t*-test). (D) Cell viability analysis of iPSC-derived neurons from familial ALS patients compared with those from healthy donors. hPSC lines used: 201B7, Wd39, and KA11 (healthy donor); A21412 and A21428 (Familial ALS carrying $TARDBP^{Q343R}$ mutation); A3411 and A3416 (Familial ALS carrying $TARDBP^{M337V}$ mutation).

Table S1 List of GO Terms
(CTraS-PSC vs untreated-PSC; a fold change differences of ± 2.0; related to Figure 2)

CTraS > untreated (1039 genes) **Biological Process** and **Molecular Function**

**GO ACCESSION**	**GO Term**	***p*-value**
GO:0010033	response to organic substance	1.13E-14
GO:0071840| GO:0071841	cellular component organization or biogenesis	3.40E-14
GO:0006695	cholesterol biosynthetic process	3.69E-13
GO:1902653	secondary alcohol biosynthetic process	6.38E-13
GO:0016126	sterol biosynthetic process	7.30E-12
GO:0042221	response to chemical	7.54E-12
GO:0008203	cholesterol metabolic process	8.83E-12
GO:1902652	secondary alcohol metabolic process	2.06E-11
GO:0009987| GO:0008151| GO:0050875	cellular process	5.42E-11
GO:0048522| GO:0051242	positive regulation of cellular process	5.24E-11
GO:0032502	developmental process	6.44E-11
GO:0044767	single-organism developmental process	6.44E-11
GO:0009719	response to endogenous stimulus	9.73E-11
GO:0046165	alcohol biosynthetic process	1.11E-10
GO:0016125	sterol metabolic process	1.44E-10
GO:0048518| GO:0043119	positive regulation of biological process	1.94E-10
GO:0048856	anatomical structure development	2.88E-10
GO:0008150| GO:0000004| GO:0007582	biological process	3.14E-10
GO:0007275	multicellular organism development	3.89E-10
GO:0009966| GO:0035466	regulation of signal transduction	1.15E-09

untreated > CTraS (1054 genes) **Biological Process** and **Molecular Function**

**GO ACCESSION**	**GO Term**	***p*-value**
GO:0060968	regulation of gene silencing	2.97E-10
GO:0004556	alpha-amylase activity	4.31E-09
GO:0071294	cellular response to zinc ion	1.86E-08
GO:0016160	amylase activity	2.91E-08
GO:0060255	regulation of macromolecule metabolic process	1.83E-05
GO:0009611| GO:0002245	response to wounding	1.97E-05

Table S2 List of Primers
(related to Figure 1, 3, 4, 7, S1, S2, S4, S5, and S6)

Gene	Forward	Reverse
OCT4	TTGGGCTCGAGAAGGATGTGGT	TGCATAGTCGCTGCTTGATCGC
NANOG	TGAACCTCAGCTACAAACAG	TGGTGGTAGGAAGAGTAAAG
PAX6	ACCACACCGGTTTCCTCCTTCACA	TTGCCATGGTGAAGCTGGGCAT
NESTIN	TTCCCTCAGCTTTCAGGACCCCAA	AAGGCTGGCACAGGTGTCTCAA
SOX1	GATCAGCAAGCGCCTGGGGG	AGCAGCGTCTTGGTCTTGCGG
BRACHYURY	TGCTTCCCTGAGACCCAGTT	GATCACTTCTTTCCTTTGCATCAAG
SOX17	TGTCCCAAAACAGCTTCCTC	TCACCCTTTTCGAGGATGAG
TUBB3	ATTTCATCTTTGGTCAGAGTGGGGC	TGCAGGCAGTCGCAGTTTTCAC
MAP2	GGATCAACGGAGAGCTGAC	TCAGGACTGCTACAGCCTCA
MAPT-Tau	CCTCTCCCGTCCTCGCCTCTG	GGGTCAGCCATCCTGGTTCA
NCAM	ATGGAAACTCTATTAAAGTGAACCTG	TAGACCTCATACTCAGCATTCCAGT
NSE	GGAGAACAGTGAAGCCTTGG	GGTCAAATGGGTCCTCAATG
SYN1	TTGCCCAGATGGTTCGACTG	TGTCAACCTTGACCTTGCCC
PSD95-DLG4	TCACAACCTCTTATTCCCAGCA	CATGGCTGTGGGGTAGTCG
SATB2	TCTCCCCCTCAGTTATGTGAC	AGGCAAGTCTTCCAACTTTGAA
TBR1	GCCTTTCTCCTTCTATCATGCTC	GTCAGTGGTCGAGATAATGGGA
CTIP2	TGGGTGCCTGCTATGACAAG	GGCTCGGACACTTTCCTGAG
VGLUT1	TACACGGCTCCTTTTTCTGG	CTGAGGGGATCAGCATGTTT
GRIA1	GGGGAGGTGATTCCAAGGAC	CCAGTTACAATCCCGTGGCT
GRIA2	TGGTACGACAAAGGAGAGTGC	ACCAGCATTGCCAAACCAAG
GRIN1	AGGAACCCCTCGGACAAGTT	CCGCACTCTCGTAGTTGTG
GRIN2A	CTTGCTTCAGTTTGTGGGTG	AGCCAGCATGTAGAATACGC
GRIN2B	TCCGTCTAGAGGTTTGGCTTC	ACACCAACCAGAACTTGGGAG
GAD65	CTGCTCCAGTCTCCAAAGCC	CCGTGAACTTCTGAGCCACT
GAD67	CGAGGACTCTGGACAGTAGAGG	GATCTTGAGCCCCAGTTTTCTG
TH	TCATCACCTGGTCACCAAGTT	GGTCGCCGTGCCTGTACT
DAT	TGAGCTCTTCACGCTCTTCA	CACCATAGAACCAGGCCACT
ISLET1	AGCAGCCCAATGACAAAACT	CTGAAAAATTGACCAGTTGCTG
HB9	GTCCACCGCGGGCATGATCC	TCTTCACCTGGGTCTCGGTGAGC
GFAP	CTGCTCAATGTCAAGCTGG	AATGGTGATCCGGTTCTCC
GIRK2	CTGGAAATTGTGGTCATC	GGTCTCATAGGTCTCATG
ACTB	TGAAGTGTGACGTGGACATC	GGAGGAGCAATGATCTTGAT

Table S3 List of Antibodies and Dilutions
(related to Figure 2, 3, 4, 5, 6, 7, S1, S5, S6, and S7)

Antibody	Dilution	Source	Catalogue number	Location
TRA-1-60	1:1000	Millipore	MAB4360	Billerica, MA, USA
SSEA4	1:1000	Abcam	ab16287	Cambridge, MA, USA
PAX6	1:500	BioLegend	901301	San Diego, CA, USA
NESTIN	1:500	described previously, refs. (Iwanami et al., 2005; Kobayashi et al., 2012; Nori et al., 2011)		
SOX1	1:500	R&D Systems	AF3369	Minneapolis, MN, USA
BRACHYURY	1:200	R&D Systems	AF2085	Minneapolis, MN, USA
SOX17	1:500	R&D Systems	AF1924	Minneapolis, MN, USA
AFP	1:200	R&D Systems	MAB1368	Minneapolis, MN, USA
αSMA	1:500	Sigma-Aldrich	A2547	St Louis, MI, USA
βIII-tubulin	1:1000	Sigma-Aldrich	T8660	St Louis, MI, USA
MAP2	1:500	Sigma-Aldrich	M4403	St Louis, MI, USA
Synapsin-1	1:500	Synaptic Systems	106103	Goettingen, Germany
TH	1:500	Millipore	AB152	Billerica, MA, USA
Complex III-Core I	1:200	Thermo Fisher Scientific	459140	Waltham, MA, USA
Phosphorylated α-synuclein	1:1000	Wako	014-20281	Osaka, Japan
Cleaved caspase-3	1:500	Cell Signaling Technology	9661	Danvers, MA, USA
DAT	1:200	Abcam	ab5990	Cambridge, MA, USA
FOXA2	1:500	R&D Systems	AF2400	Minneapolis, MN, USA
LMX1A	1:100	Abcam	Ab139726	Cambridge, MA, USA
NURR1	1:200	Santa Cruz Biotechnology	sc-991	Dallas, TX, USA
PITX3	1:200	Millipore	AB5722	Billerica, MA, USA
DDC	1:100	R&D Systems	AF3564	Minneapolis, MN, USA
D2R	1:1000	Abcam	ab21218	Cambridge, MA, USA
GFAP	1:4000	DAKO	Z033401	Glostrup, DK, USA
FOXG1	1:500	Abcam	MAB1585	Cambridge, MA, USA
OTX2	1:100	R&D Systems	AF1979	Minneapolis, MN, USA
EN1	1:100	Abcam	ab117549	Cambridge, MA, USA
HOXB4	1:100	DSHB	I12	Iowa City, IA, USA
PAX7	1:100	Abcam	ab34360	Cambridge, MA, USA
NKX2.2	1:100	DSHB	74.5A5	Iowa City, IA, USA

SUPPLEMENTAL EXPERIMENTAL PROCEDURES

Neural Induction *In Vitro*

For *in vitro* differentiation, hPSCs were cultured in standard hESC medium either alone or supplemented with 3 μM SB431542 (Tocris Bioscience), 3 μM dorsomorphin (Sigma-Aldrich) and 3 μM CHIR99021 (Stemgent) for 5 days. The medium was changed daily. On day 5, hPSC colonies were detached from the feeder layers using a dissociation solution (0.25% trypsin, 100 μg/ml collagenase IV (Invitrogen), 1 mM $CaCl_2$, and 20% KSR) and were enzymatically dissociated into single cells using TrypLE Select (Life Technologies). The dissociated cells were cultured in suspension at a density of 1×10^5 cells/ml in Ultra-Low attachment culture dishes (Greiner) in proliferation medium consisting of serum-free medium (media hormone mix; MHM) supplemented with 2% B27 supplement (Invitrogen), 20 ng/ml FGF, 10 ng/ml hLIF (Millipore), 2 μM SB431542 and 3 μM CHIR99021 in a hypoxic and humidified atmosphere (4% O_2, 5% CO_2). The medium was changed every 2-3 days for approximately 3-7 days to induce the formation of NSs.

To differentiate neuronal cells, dissociated NSs were plated onto either a 96-well plate or coverslips 10 mm in diameter coated with poly-L-ornithine (PO) and growth-factor-reduced Matrigel (50x dilution, thin coated; Invitrogen). These cells were cultured in differentiation medium consisting of MHM supplemented with 2% B27 supplement (Thermo Fisher), 10 ng/ml rhBDNF (R&D Systems), 10 ng/ml rhGDNF (R&D Systems), 0.2 mM ascorbic acid (Sigma-Aldrich) and 2 μM DAPT (Sigma-Aldrich) for 10-15 days in a humidified atmosphere containing 5% CO_2. Half of the medium was changed every 2–3 days.

Immunocytochemistry

Cells were fixed in phosphate-buffered saline (PBS) containing 4% paraformaldehyde (PFA) for 30 min at room temperature. Thereafter, all the cells were blocked with 5% FBS and Triton X-100 and incubated with the primary antibodies described in Supplementary Table 1. The cells were then rinsed with PBS and incubated with species-specific Alexa Fluor 488-, Alexa Fluor 555-, or Alexa Fluor 647-conjugated secondary antibodies (1:500; Invitrogen), which was followed by the addition of Hoechst 33258 (0.5 μg/mL; Sigma-Aldrich) to counterstain the nuclei. The images were obtained using an Axioplan2 (Carl Zeiss AG), LSM-710 (Carl Zeiss AG), BZ9000 (Keyence), or IN Cell Analyzer 6000 (GE Healthcare).

High-Content Analysis

For the cell population assays, fluorescence intensity analysis, neural maturation analysis, and ROS production assays, stained plates were imaged on an IN Cell Analyzer 6000 high-content cellular analysis system (GE Healthcare), and a set of 5 x 5 fields were collected from each well using the 20x objective, resulting in the scoring of over 10,000 cells per well. For the mitochondrial function assay, stained plates were imaged on an IN Cell Analyzer 6000, and a set of 6 x 6 fields were collected from each well using the 60x objective, resulting in the scoring of over 9,000 cells per well. Analysis (IN Cell Developer Toolbox v1.9; GE Healthcare) was conducted by identifying intact nuclei stained with Hoechst; these

nuclei were defined as traced nuclei that were larger than 50 μm^2 in surface area and with typical intensity levels that were lower than the threshold brightness of pyknotic cells. Each traced nuclear region was then expanded by 50% and cross-referenced with endodermal markers (SOX17 and AFP), mesodermal markers (BRACHYURY and αSMA), ectodermal markers (PAX6, NESTIN, and βIII-TUBULIN), an NS/PC marker (SOX1), neuronal markers (MAP2 and βIII-TUBULIN), and pluripotent markers (SSEA4 and TRA-1-60) to identify the cell type; from these images, the percentages and fluorescence intensities of these cells were calculated.

Using the above described traced images of each cell, the cell population CTraS-PSCs, CTraS-NSs, and CTraS-Neurons; the fluorescence intensity of the markers of all three germ layers in the CTraS-PSC and EB differentiation cultures; the CellROX fluorescence intensity of the neurons; the cleaved caspase-3 intensity and the area of the TH intensities in neurons were analyzed. By setting the areas on βIII-TUBULIN$^+$ cells, the number of synapsin1$^+$ puncta in neurons was analyzed.

Neurosphere Formation Assay

Neurosphere (NS) cultures were fixed and immunostained by SOX1 1 hour after plating the dispersed NS cultures, which were cultivated for 12 hours (Day 5), 3 days (Day 8), 5 days (Day 10), or 10 days (Day 15) after NS induction.

The analysis (IN Cell Developer Toolbox v1.9; GE Healthcare) began by identifying intact nuclei stained with Hoechst (Ho), which were defined as traced nuclei that were larger than 50 μm^2 in surface area and with typical intensity levels that were lower than the threshold brightness of pyknotic cells. Up to 1000 of these Ho$^+$ cells were counted, and the NS formation assay was performed using this cell population. The NS size was assessed by measuring the NS diameter (> 50 μm) and surface area (> 1000 μm^2) using an IN Cell Analyzer 6000 System (GE Healthcare), and only SOX1$^+$ NSs were calculated.

Microarray Analysis

Total RNA was extracted using an RNeasy Kit (QIAGEN), and the RNA quality was assessed using an Agilent 2100 Bioanalyzer (Agilent Technologies). Total RNA (100 ng) was reverse-transcribed, labeled with biotin using a 3’IVT Express Kit (Affymetrix) and hybridized to a GeneChip® Human Genome U133 plus 2.0 Array (Affymetrix). The arrays were washed and stained using a GeneChip Fluidics Station 450 (Affymetrix) and then scanned with a GeneChip Scanner 3000 7G System (Affymetrix) according to the manufacturer’s instructions. The raw probe intensity files were normalized to MAS5 and log (base2) transformed using GeneSpring GX 13.1 software (Agilent Technologies). The gene set was filtered based on the expression levels to remove genes that were not expressed in all the samples. PCA was performed using the normalized data. For the hierarchical clustering, the normalized data were calculated based on Euclidean correlations with average linkages.

The data discussed in this publication have been deposited into the NCBI Gene Expression Omnibus (GEO http://www.ncbi.nlm.nih.gov/geo/) database and are accessible using GEO Series accession number

GSE95271.

Quantitative RT-PCR

Total RNA was isolated with a RNeasy mini kit (QIAGEN) and treated with DNase I, and cDNA was prepared using a ReverTraAce qPCR RT kit (Toyobo). Quantitative RT-PCR was performed using SYBR Premix Ex Taq II (Takara Bio) on a ViiA 7 Real-Time PCR System (Life Technologies) (Supplementary Table 2).

Microelectrode Array Recording

Microelectrode array (MEA) recording was performed using a Maestro system (Axion Biosystems). To prepare the neurons for array recording, single-cell dissociated NSs were subsequently plated onto the electrode area in a Matrigel (Corning)-coated MEA plate at a density of 1.5×10^5 cells/cm^2 per well. Once the cells were attached, MHM supplemented with 2% B27 supplement (Thermo Fisher), 10 ng/ml rhBDNF (R&D Systems), 10 ng/ml rhGDNF (R&D Systems), 0.2 mM ascorbic acid (Sigma-Aldrich) and 2 μM DAPT (Sigma-Aldrich) was added to the well. The medium was subsequently changed every 2-3 days. The data were acquired using a sampling rate of 12.5 kHz and filtered using a 200-3000 Hz Butterworth bandpass filter. A detection threshold was set to +6.0 times the SD of the baseline electrode noise. The spike count files generated from the recordings were used to calculate the number of active electrodes (defined as an electrode having an average of more than 5 spikes/min) in each well, the average per-active electrode mean firing rate (MFR; spikes/min) and the standard deviation of the average per-active electrode MFR. The data from the initial 3 min in each data file were omitted to enable the activity to stabilize in the Maestro, and 10–15 min of activity was subsequently recorded.

Cell Viability Analysis

An MTT Cell Viability Assay Kit (BioAssay Systems) was used to evaluate the cell viability of hiPSC-derived neurons. In all the experiments, single-cell dissociated NSs were seeded into 96-well plates at a density of 1.5×10^5 cells/cm^2 per well. At five days after NS seeding, the MTT assay was performed every fifth day according to the manufacturer's protocol. MTT (15 μL/well) was added to the wells and incubated at 37°C for 4 h. The supernatant was removed, and 100 μL of solubilizer per well was added to dissolve the produced formazan. After shaking the plates for 10 min, the absorbance values of the wells were recorded with a microplate reader at a wavelength of 570 nm.

SUPPLEMENTAL REFERENCES

Iwanami, A., Kaneko, S., Nakamura, M., Kanemura, Y., Mori, H., Kobayashi, S., Yamasaki, M., Momoshima, S., Ishii, H., Ando, K.*, et al.* (2005). Transplantation of human neural stem cells for spinal cord injury in primates. Journal of neuroscience research *80*, 182-190.

Kobayashi, Y., Okada, Y., Itakura, G., Iwai, H., Nishimura, S., Yasuda, A., Nori, S., Hikishima, K., Konomi, T., Fujiyoshi, K.*, et al.* (2012). Pre-evaluated safe human iPSC-derived neural stem cells promote functional recovery after spinal cord injury in common marmoset without tumorigenicity. PloS one *7*, e52787.

Nori, S., Okada, Y., Yasuda, A., Tsuji, O., Takahashi, Y., Kobayashi, Y., Fujiyoshi, K., Koike, M., Uchiyama, Y., Ikeda, E.*, et al.* (2011). Grafted human-induced pluripotent stem-cell-derived neurospheres promote motor functional recovery after spinal cord injury in mice. Proceedings of the National Academy of Sciences of the United States of America *108*, 16825-16830.
